# Social class and wise reasoning about interpersonal conflicts across regions, persons and situations

**DOI:** 10.1098/rspb.2017.1870

**Published:** 2017-12-20

**Authors:** Justin P. Brienza, Igor Grossmann

**Affiliations:** Department of Psychology, University of Waterloo, 200 University Avenue West, Waterloo, Ontario, Canada N2L 3G1

**Keywords:** social class, social status, reasoning, wisdom, conflict, ecology

## Abstract

We propose that class is inversely related to a propensity for using wise reasoning (recognizing limits of their knowledge, consider world in flux and change, acknowledges and integrate different perspectives) in interpersonal situations, contrary to established class advantage in abstract cognition. Two studies—an online survey from regions differing in economic affluence (*n* = 2 145) and a representative in-lab study with stratified sampling of adults from working and middle-class backgrounds (*n* = 299)—tested this proposition, indicating that higher social class consistently related to lower levels of wise reasoning across different levels of analysis, including regional and individual differences, and subjective construal of specific situations. The results held across personal and standardized hypothetical situations, across self-reported and observed wise reasoning, and when controlling for fluid and crystallized cognitive abilities. Consistent with an ecological framework, class differences in wise reasoning were specific to interpersonal (versus societal) conflicts. These findings suggest that higher social class weighs individuals down by providing the ecological constraints that undermine wise reasoning about interpersonal affairs.

## 1. Introduction

How do people of different social class vary in their reasoning style? For at least a century, this question has been at the core of scholarship on mental abilities [1,2]. Some research has suggested that people of higher social class exhibit a superior style of reasoning, with white-collars performing better on tasks measuring fluid and crystallized intelligence compared with blue-collars [2,3]. A dominant explanation for this observation has involved differences in ecological affordances, with lower-class environments defined by fewer resources, greater threat, and more uncertainty [4–9]—all factors that inhibit performance on abstract intelligence tests—suggesting that lower-class environments promote inferior reasoning. Here, we advance an alternative account, with a focus on wisdom-related pragmatic reasoning [10,11] rather than abstract reasoning such as propositional logic [12]. Central aspects of this reasoning style include intellectual humility, recognition that the world is in flux and changes, and the ability to take different contexts into account besides one's own—factors philosophers have long associated with handling situations wisely [13–16]. To address the question of class differences in wise reasoning, we use a multi-method approach, including a recently validated, psychometrically robust method for assessing wise reasoning style when reflecting on interpersonal experiences people encounter in their lives [17], and observer-rated judgements of performance on stream-of-thought reports on standardized interpersonal situations [14]. Contrary to findings concerning differences on standardized IQ tests, the present research indicates systematic regional, individual-difference and situational effects of wiser reasoning style in lower- versus higher-class contexts. The current insights qualify the complex relationship between socio-cultural environments and interpersonal reasoning style.

The concept of wise reasoning has recently emerged in behavioural sciences [13,14,18], highlighting the combined utility of certain metacognitive strategies when navigating uncertainties people face in their lives [15]. Such strategies include the appreciation of contexts broader than the immediate issue, sensitivity to the possibility of change in social relations, intellectual humility and search for a compromise between different points of view [14,19,20]. Individual differences in wise reasoning are only weakly related to dispositional empathy and perspective-taking [17], and promote prosocial tendencies in the process of deliberation [17,18,21]. Even though abstract cognition assessed with domain-general intelligence tests may provide higher-class individuals with a stronger foundation for wise reasoning than their lower-class counterparts, domain-general IQ tests are not equivalent with wise reasoning [11,15,22], raising a question about whether social class differences in wise reasoning would mirror results from standardized IQ tests.

A diverging propensity for abstract cognition as compared to wise reasoning is consistent with evolutionary [23] and ecological [24] theorizing on how class-specific behaviours reflect adaptations to different environments. Some behaviours associated with lower class, which at first glance may appear poorly reasoned, may be adaptive responses to the resource-related ecological constraints faced by people of lower class [25–27]. For instance, compared to more stable middle-class environments, the greater instability and adversity of working class environments may encourage shorter-term life-history strategies [28]. From this perspective, not delaying rewards, typically conceptualized as self-regulation failure, does not necessarily appear maladaptive [23,29,30]. Pertinent to the present investigation, compared to the middle class, the working class and the poor are more likely to focus on close relationships (versus individuality) and in-group cooperation (versus competition) [28,31–34]—ecological adaptations that secure survival and success in resource-poor environments. Indeed, studies of socialization patterns indicate that working-class parents are less likely to provide their children with support beyond adolescence, thereby affording less room for subjective feeling of entitlement fostered by middle and upper class upbringing [35]. Working-class people also show a broader attentional focus and heightened sensitivity to contextual cues [36,37], which are adaptive strategies when environments are threatening, and resources and opportunities are fleeting [30,38,39]. Building on social class differences in attentional, social and socialization strategies, we propose that wise reasoning about interpersonal affairs may be more prevalent in lower- compared with higher-class environments, because it may enable navigation and management of uncertainties surrounding such environments [13,14]. Moreover, because greater self-focus can attenuate wise reasoning [40,41], higher-class environments (which promote self-focus) may detriment higher-class individuals' propensity of using wise reasoning. The present ecological framework further suggests that class differences in wise reasoning would be specific to the ecologically-relevant *interpersonal* domain (versus domain-general), functional for in-group coordination and other survival-related activities [30].

## 2. Study 1

**Figure 1.**
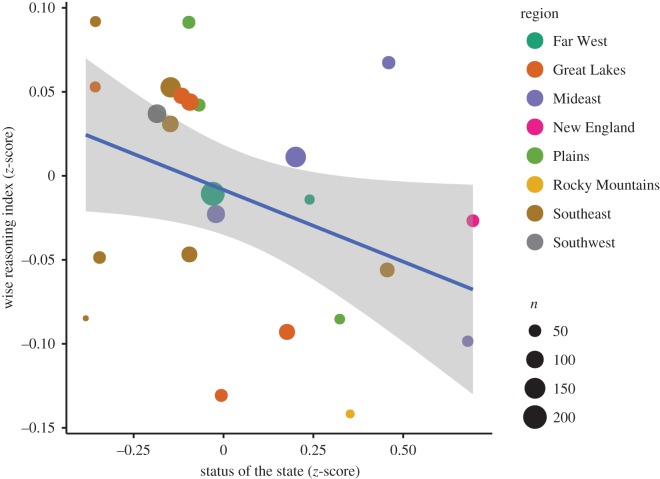
Lower levels of wise reasoning observed in states with higher average social class. *n* = number of participants from each state. Colours represent regions, as classified by the US Bureau of Economic Analysis (www.bea.gov/regional). We include states with *n* ≥ 25, with comparable results with other cut-offs (see electronic supplementary material). (Online version in colour.)

To explore the relationship between social class and wise reasoning, we conducted a large-scale online survey (*n* = 2145) of wise reasoning style among US residents from regions differing in socio-economic affluence (see figure 1 and electronic supplementary material, table S1). To avoid bias due to class-related differences in domain-specific knowledge, we focused on mundane interpersonal experiences both middle and working class people are likely to encounter in their lives, assessing wise reasoning about interpersonal conflicts. We tested the relationship between social class and wise reasoning across the group, individual, and situational levels of analysis [42]. Given *a priori* independence of cognitive and affective responses across the group, individual, and situational levels [42–45], probing possible social class differences across these different levels of analysis allowed us to triangulate on whether the impact of social class ecology on wise reasoning is additive or interactive [46].

### (a) Methods

We recruited participants from Amazon.com's Mechanical Turk (MTurk). Samples were taken from projects exploring the psychometric properties of the wise reasoning instrument [17] (*n* = 1960) and the relationship of wise reasoning to prosociality [21] (*n* = 250). To avoid order effects, we only included participants from studies in which wise reasoning instrument preceded other measures. Demographic information was collected last. We used participants' IP addresses to estimate which state they were located in. To ensure that no participant would be included more than once, 65 duplicate IP addresses were removed from the current analyses, leaving a total of 2145 responses. The majority of US states included at least 15 participants (see electronic supplementary material, figure S1). State-specific sample size closely mirrored state-specific population (*r* = 0.94), suggesting representative weighting of participants per state.

#### (i) Measures

*Wise reasoning.* To assess wise reasoning in an ecological and unbiased fashion, we asked participants to recall recent experiences from their lives (with a friend or in the workplace). To ensure accuracy of recall, we cued participants to reconstruct the context of their experience using the event-reconstruction method [47], including specific details about the time, space, and persons involved in the experience [17]. Subsequently, participants responded to 21 items asking them the extent to which they engaged in one of the five aspects of wise reasoning style (1 = *not at all* to 5 = *very much*): (i) recognition of the limits of one's own knowledge and intellectual humility; (ii) recognition of world in flux and change, and consideration of multiple ways a situation could unfold; (iii) application of an outsider's vantage point; (iv) recognition of others' perspectives; and (v) consideration of/search for compromise and recognition of importance of conflict resolution. As reported in the large-scale psychometric evaluation of the instrument [17], this method shows stronger and more reliable predictive validity as well as greater independence from biased responding than all other major measures of wisdom-related qualities, and shows small-to-moderate relations to measures of general other-orientation (e.g. agreeableness, attention to others' emotions, empathy).

In our analyses, we first evaluated model fit with the *lavaan* package in R. To this end, we fed first-order factor scores for each of the five facets into a second-order factor of wise reasoning (see electronic supplementary material, figure S2 and tables S2 and S3). We saved estimated factor scores of the first- and second-order factors for subsequent analyses. Employing the average score across 21 items did not change the pattern of results.

*US state-level social class.* Drawing on recent behavioural research on social class [28,31,46], we conceptualize the construct broadly. Specifically, given a continuing debate about social class measurement [48,49], we aimed to remain agnostic about superiority of a particular marker of social class by collecting a range of metrics concerning resource-deprivation, psychological attitudes, and sociological markers developed to compare population-wide distribution of social class, as well as individual markers of education and income, which we used in subsequent analyses. We collected data concerning group-level resource-deprivation based on the 2014 state-level percentages of Americans who were uninsured. To obtain a psychological marker of reactivity to resource-deprivation, we gathered data from the Gallup (gallup.com) concerning state-level expression of moderate to high levels of worries about money on at least 3/7 financial issues (retirement, medical costs for serious illness/injury, maintaining their standard of living, medical costs for normal healthcare, monthly bills, housing costs, and minimum credit card payments). As a sociological marker, we drew from the 2014 American Community Survey (usa.ipums.org) to estimate state-level median Nam-Powers-Boyd occupational status. The Nam-Powers-Boyd 1990 scores represent one of the more recent demographic estimates of occupational status, aiming to account for median earnings and median educational attainment associated with each major occupational category based on 1990 occupational classification by the same authors [50]. These scores give equal weights to education and earnings. On the state-level, aggregated medians of these scores reflect a standardized ranking of states in terms of typical income and education of civilian labour force in each state.

We estimated a structural equation model, with each of the above state-level indices of social class feeding into a latent factor of state-level social class (see electronic supplementary material, figure S2) and estimated factor scores for subsequent analyses. As separate control analyses, we examined effect of the Nam-Powers-Boyd occupational status index alone, as well as the impact of the state-level estimates based on participants' education and income, which we discuss below.

*Individual-level social class.* Measurement of individual-level social class is complex, as it involves an intersection of different factors, including ownership of capital assets and possession of skills or credential assets [49]. Relevant to the present research, psychologists have used education and income as central markers of behavioural social class studies [28,31,43,51]. To accurately model the interaction of these factors, we performed a parallel estimation of the individual-level social class via structural equation modelling, with participants' reported income (a marker of capital assets) and education (a marker of skills/credential assets) feeding into a latent factor of individual-level status (see electronic supplementary material, figure S3). We estimated individual-level class score, saving the resulting parameter estimates as an index of individual-level social class (see electronic supplementary material). Notably, group-level averages of individual-level social class estimates were highly correlated with population-based estimates of social class across US states (*r* = 0.96), indicating that the present sample was highly representative of the social class of the average person from the respective states, and suggesting a high degree of convergence across different measures of social class employed in Study 1.

*Situation-level status/subjective class and interpersonal closeness.* To examine subjective social class and level of interpersonal closeness, a subset of participants (*n* = 730) answered the following questions regarding their interpersonal experience: (i) ‘Did the other person have more status than you?’ (1 = *much less*, 2 = *less,* 3 = *same or similar*, 4 = *more*, 5 = *much more*), and (ii) ‘Were you close to the person before the incident?’ (1 = *no*, 2 = *somewhat*, 3 = *yes*, 4 = *very close*).

*Controls.* We controlled for several characteristics of regions and individuals that could be correlated with status and, thus, cause spurious associations: population size, percentage of residents living in urban centres, income inequality, scholastic aptitude, as well as age, gender and social desirability (see electronic supplementary material for methods and procedures).

### (b) Analytical procedure

To estimate underlying latent factors of wise reasoning and regional/individual social status, we employed structural equation analyses with maximum-likelihood parameter estimates. To ensure robustness to non-normality in this process, we employed robust standard errors and mean-adjusted chi-square test statistics. Subsequently, we used estimates from structural equation analyses in inferential analyses involving two-sided statistical tests. On the state- and situation-specific levels of analysis, our main analyses included correlations and linear regressions. To probe robustness of group-level results as a subject of number of participants available per state, we (i) examined the group-level relationship between status and wise reasoning at three different cut-off points for minimum number of participants per state (see electronic supplementary material), and (ii) performed a random intercept mixed effect analysis on all available data, with participants nested in respective states. We observed greater variability in wise reasoning at the within-state (s.e. = 0.013) as compared to the between-state level (s.e. = 0.003). To account for the nested data structure of the individual-level estimates, we conducted parallel random intercept mixed-effect analyses with participants' responses nested in respective states. We estimated indirect effects via the *mediation* package in R.

### (c) Results

#### (i) State-level analyses

First, we explored the distribution of wise reasoning about interpersonal conflicts across states differing in psychological and sociological markers of social class. As figure 1 indicates, people from states with higher average social class were less likely to use wise reasoning style about interpersonal experiences from their lives as compared to people from states with lower average social class, *r* = −0.39. This association was consistent when examining different cut-offs, −0.39 ≤ *r* < 0.35, when using occupational index alone, *r* = −0.34, when examining state-level averages of participants' social class based on education and income instead of population-based estimates, *r* = −0.30, and consistent across each facet of wise reasoning, *r*_humility_ =−0.37, *r*_outsider viewpoint_ =−0.52, *r*_change_ =−0.28, *r*_perspectives_ =−0.30, *r*_compromise_ =−0.27. Similarly, results were consistent when examining random intercept mixed effects models with participants' scores nested within states on the full sample (see electronic supplementary material, table S4).

Moreover, state-level status remained a robust negative predictor of wise reasoning when controlling for correlates of social class, including population density, *B* = −0.161, s.e. = 0.045, *t*(d.f. = 2145) = −3.573, *p* = 0.0004, urbanization, *B* = −0.161, s.e. = 0.045, *t*(d.f. = 2145) = −3.571, *p* = 0.0004, income inequality, *B* = −0.163, s.e. = 0.046, *t*(d.f. = 2145) = −3.573, *p* = 0.0004, status × inequality interaction, *B* = −0.168, s.e. = 0.046, *t*(d.f. = 2145) = −3.649, *p* = 0.0003, state-level differences in domain-general reasoning (as captured by the Scholastic Aptitude Test), *B* = −0.163, s.e. = 0.045, *t*(d.f. = 2145) = −3.603, *p* = 0.003, and social desirability, *B* = −0.304, s.e. = 0.080, *t*(d.f. = 637) = −3.809, *p* = 0.002.

#### (ii) Individual-level analyses

Because of substantial within-state variability in social class, in the next step we examined how individual-level indicators of social class, estimated from person's education and income, were associated with wise reasoning. We performed a separate set of random intercept mixed effects analyses with participants nested in states and individual-level social class as a predictor of wise reasoning about interpersonal conflicts.

Higher individual social class was significantly negatively associated with wise reasoning (see electronic supplementary material, figure S4 and table S4). Effect of individual status on wise reasoning was robust when controlling for gender and age, *B* = −0.218, s.e. = 0.021, *t*(d.f. = 2144) = −4.833, *p* < 0.0001, social desirability, *B* = −0.273, s.e. = 0.069, *t*(d.f. = 637) = −3.971, *p* < 0.0001, agreeableness, *B* = −0.246, s.e. = 0.058, *t*(d.f. = 833) = −4.204, *p* < 0.0001, openness, *B* = −0.254, s.e. = .058, *t*(d.f. = 833) = −4.366, *p* < 0.0001, and tendency to focus on others' emotions, *B* = −0.271, s.e. = 0.067, *t*(d.f. = 637) = −4.022, *p* < 0.0001.

To simultaneously assess independent effects of state- and individual-level social class on wise reasoning, we state-level class estimate by averaging social class within each state, and created individual-level estimates by obtaining the difference scores between participants' social class and state's average. We used these scores and their interaction as predictors of wise reasoning in random intercept mixed effect analyses, with participants nested in respective states. The results from this model indicated independent negative effects on wise reasoning at each level of social class, state-level: *B* = −0.259, s.e. = 0.125, *t*(d.f. = 2145) = −2.067, *p* = 0.039, individual-level: *B* = −0.220, s.e. = 0.045, *t*(d.f. = 2145) = −4.934, *p* < 0.0001, with no significant cross-level interaction, *t* < 1.

#### (iii) Situation-specific analyses and mediation through interpersonal closeness

**Figure 2.**
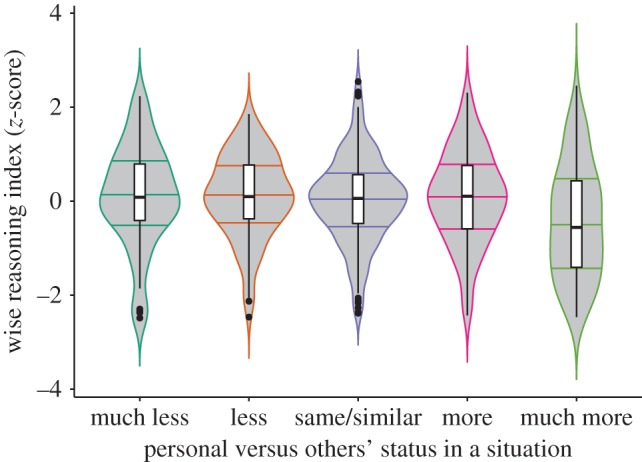
Lower levels of wise reasoning observed in situations with higher status (compared to the other person in the interpersonal conflict). Violin-plots with the median, and 1st and 3rd quantiles and boxplots. (Online version in colour.)

Finally, we examined whether situations in which participants reported being in a higher (versus low) status position were negatively associated with wise reasoning. To examine this question, we used participants' responses regarding their relative status, or subjective social class [52], which they reported immediately following the wise reasoning assessment. As figure 2 and electronic supplementary material, table S4 indicate, the higher-class position was significantly negatively associated with wise reasoning (also see electronic supplementary material, figure S5). Simultaneously entering mean-centred individual-level social class and situation-level status as predictors of wise reasoning in a random intercept mixed-effect analyses, with participants nested in respective states, indicated independent negative effects on wise reasoning at each level of social class, individual-level: *B* = −0.479, s.e. = 0.152, *t*(d.f. = 558.1) = −3.156, *p* = 0.002, situation-level: *B* = −0.110, s.e. = 0.032, *t*(d.f. = 729.5) = −3.414, *p* < 0.001, with no significant interaction, *t* < 1.

Past research indicates higher social class relates to lower levels of interpersonal closeness [31,46] and less sensitivity to socio-emotional cues [37,53]. Because wise reasoning style may be more accessible when interpersonal considerations are salient [21,54,55], we estimated whether situation-specific status differences in interpersonal orientation account for effects of subjective class on wise reasoning style. Results of mediation analyses, presented in electronic supplementary material, table S5, indicated that this was the case. Specifically, subjective social class was negatively related to wise reasoning about the interpersonal conflict, in part because of greater perceived interpersonal distance between the participant and the other person in the conflict. This indirect effect accounted for 13.55% of the status effect in wise reasoning. It was comparable across all facets of wise reasoning (see electronic supplementary material, table S5).

## 3. Study 2

Although the findings from Study 1 showed consistent negative effects of social class on wise reasoning across three different levels of analysis, they were observed using an online sample of convenience, which may have biased results through atypical sub-samples of working and middle-class participants. Thus, in Study 2 we obtained data from a recent behavioural study of abstract cognitive abilities and wise reasoning among random stratified samples of adults from Michigan [56]. This study involved standardized naturalistic vignettes depicting interpersonal and intergroup conflicts, thereby controlling for content of reasoning and allowing for analyses regarding the domain-specificity of the effect of social class on wise reasoning. Participants verbally reflected on conflicts depicted in vignettes, guided by several prompts. Independent coders, blind to socio-demographic information from the sample coded behavioural responses on key dimensions of wise reasoning [14], equivalent to those employed in Study 1, and competed established measures of fluid and crystallized cognitive abilities.

### (a) Method

In 2007–2009, the senior author recruited a probability sample from a Washtenaw county in Michigan [56]. A wide range of social class—from the nonworking poor to the affluent—was represented. Participants' names were randomly selected from a telephone directory and were sent out personalized letters, inviting them to participate in the study and announcing that researchers would also attempt to contact them by phone. The procedure resulted in 199 participants who completed both the measures of abstract cognitive abilities and wise reasoning about interpersonal and societal conflicts. See [56] for further recruitment details.

#### (i) Measures

*Cognitive tasks*. Participants completed measures of crystallized or knowledge-based intelligence using the comprehension and vocabulary subtests of the WAIS, and measures of fluid or working memory- and speed-related intelligence using the digit span subtest of WAIS and two processing speed tasks [56]. As in prior research, the respective scores were standartized and averaged into indices of fluid and crystallized cognitive abilities.

*Wise reasoning interviews*. To assess reasoning about interpersonal conflicts, participants read three authentic, detailed letters addressed to an advice columnist (‘Dear Abby’; letter length, 145–180 words), which described relational conflicts between siblings, friends, and spouses. The interviewer instructed participants to talk about future developments in these relationships, guided by four questions: (i) ‘How did the story develop after this letter?’; (ii) ‘Why do you think it happened as you said?’; (iii) ‘What was the final outcome of this conflict?’; and (iv) ‘What do you think should be done in this situation?’ After responses were transcribed and socio-demographic information removed from transcripts, two trained coders blind to the hypothesis and to the age, gender, and social class of the participants judged the responses for each story for the use of the wise reasoning categories (1 = *not at all*, to 3 = *a lot*).

To assess reasoning about intergroup conflicts, the same participants also completed another interview session concerning discussion of fictional newspaper articles depicting a fictitious conflicts between two equally strong groups from an unfamiliar country. The topics were chosen to be relevant to contemporary social issues, and included ethnic tension over political power, conflict over immigration, and conflict over natural resources. After each article the interviewer provided a brief verbal summary of the article and asked three questions: ‘What do you think will happen after that?’ and ‘Why do you think it will happen this way?’, and the additional probe, ‘Anything else?’ As for interpersonal conflicts, recorded conversations were transcribed and content-analysed by independent raters on the same dimensions of wise reasoning. We analysed standartized (*z*-scored) average responses across individual aspects of wise reasoning, along with supplementary analyses on individual dimensions. Further details concerning methods, procedure and reliability of estimates is reported in [56].

*Demographics*. Following insights by demographers that education is a central, ‘culture-carrying’ marker of social class [35,46,48,51], and frequent use of education as a marker of social class in the psychological scholarship [31,46], we used education (1 = *no college*, 2 = *some college*, 3 = *completed college*, 4 = *post-graduate degree*) as a marker of social class in our analyses. The same participants indicated their age, gender, which we used as control variables in our analyses.

*Control variables*. The same participants completed a host of measures concerning the syndromes of individualism and collectivism [43], allowing for analyses controlling for individual differences in self-construal [57], subjective closeness to family versus strangers [58], and sensitivity to social cues in vocal tone [59].

### (b) Results

Replicating prior research, lower level of education was associated with lower scores on both fluid and crystallized intelligence tasks, fluid IQ: *F*_3,194_ = 6.55, *p <* 0.001, 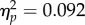, crystallized IQ: *F*_3,194_ = 15.23, *p* < 0.0001, 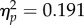, such that participants who did not attend college scored on average 0.75 s.d. lower on tests of fluid cognitive abilities and 1.4 s.d. lower on tests of crystallized cognitive abilities compared to participants who completed a post-graduate degree. Further, older age was associated with lower performance on tasks capturing fluid abilities, *B* = −0.024, s.e. = 0.004, *t*(d.f. = 197) = −5.988, *p* < 0.0001, 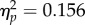, but not crystallized abilities, *B* = 0.001, s.e. = 0.004, *t*(d.f. = 197) = 0.333, *ns.* There were no significant gender differences on these cognitive tasks, *F* < 1.187.

**Figure 3.**
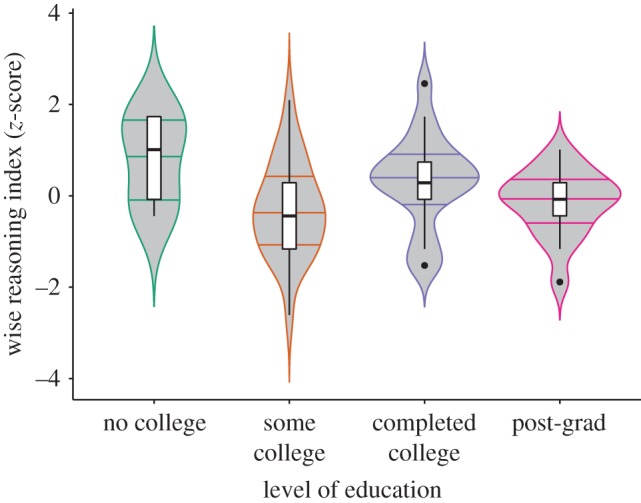
More educated participants were less likely to express wise reasoning about interpersonal conflicts (Study 2). Violin-plots with the median, and 1st and 3rd quantiles and boxplots. (Online version in colour.)

Next, we examined how performance on wise reasoning tasks varied as a function of educational attainment, simultaneously controlling for gender, number of words in participants narratives, as well as fluid and crystallized abilities. Both crystallized abilities, *B* = 0.356, s.e. = 0.090, *t*(d.f. = 197) = 3.940, *p* < 0.0001, 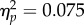, and word count, *B* = 0.002, s.e. = 0.001, *t*(d.f. = 197) = 2.454, *p* = 0.015, 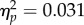, were significantly positively associated with wise reasoning about interpersonal conflicts. Importantly, we also observed a significant main effect of education, *F*_3,191_ = 3.131, *p* = 0.027, 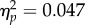. As figure 3 and electronic supplementary material, figure S6 indicate, participants without college education showed a significantly higher level of wise reasoning as compared to participants who attended college. Further tests indicated that participants without college education scored almost 0.5 s.d. higher than the rest of the sample, *B* = 0.416, s.e. = 0.205, 95% CI [0.011, 0.820], *p* = 0.044, with the largest difference between no-college and some college groups, *B* = 0.627, s.e. = 0.195, *p* = 0.014, and no significant difference between other groups. The effect of education was comparable when controlling for individualism-collectivism, self-construal: *F*_3,188_ = 2.217, *p* = 0.040, relative closeness to family versus strangers: *F*_3,177_ = 2.923, *p* = 0.035, and sensitivity to vocal tone: *F*_3,179_ = 2.898, *p* = 0.036.

#### (i) Specificity of education effects: analysis of reasoning about societal conflicts

We performed equivalent analyses for wise reasoning about societal conflicts, observing positive effects of word count, *B* = 0.002, s.e. = 0.0005, *t*(d.f. = 196) = 4.597, *p* < 0.0001, 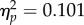, and crystallized cognitive abilities, *B* = 0.191, s.e. *=* 0.089, *t*(d.f. = 196) = 2.15, *p* = 0.033, 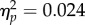. Notably, we observed no significant effect of education on wise reasoning about societal conflicts, *F*_3,189_ = 0.977.

## 4. Discussion and conclusion

In contrast to a long line of research suggesting that higher social class is aligned with superior cognition [2,3], the present data indicated that higher class is associated with a lower propensity of reasoning wisely in interpersonal situations. Our results were systematic across group, individual and situational levels of analysis when controlling for regional differences in scholastic aptitude, population, urbanization, income inequality, demographic factors such as age and gender, and a host of individual differences in agreeableness, openness to new experiences, consideration of others' emotions and individualism–collectivism. The present results were robust across different levels of analysis (group versus individual differences versus situations), methods (online autobiographic survey and content-analyses of standardized in-lab interviews) and analytical procedures (correlations, ordinary least square regressions, and linear mixed effect models with random coefficients). The present results were robust when examining markers of social class employed by behavioural scientists [28,31], demographers [50], and corresponding markers of social status on the level of a situation [53]. Notably, these results could not be accounted for by social desirability tendencies and occurred systematically across facets of wise reasoning. These analyses indicated that the negative relationship between social class and wise reasoning was not due to the potentially greater motivation of lower-class individuals to perform well on the task, nor were they fully accounted by a general orientation toward and closeness to other people, despite some shared variance with the latter process.

The consistency of effects of social class on wise reasoning across the group, individual, and situational levels of analysis is noteworthy given the potential independence of how social class may impact psychological processes at different levels of analysis [43–45]. First, the group-level results suggest that middle-class ecologies encourage less wise reasoning about interpersonal affairs than do working class ecologies (Study 1). In addition to such cultural-ecological differences, higher social class of an individual contributes to lower propensity to reason wisely about their interpersonal conflicts they encounter in their lives. In other words, above and beyond state differences in dominant social class ecology, individuals’ social class matters for their propensity for wise reasoning (Studies 1–2). Finally, situational effects explained unique variance in wise reasoning, showing that one is less likely to reason wisely when the other person involved in the situation is of lower status than oneself (Study 1). Overall, the triangulation across different levels of analysis paints an additive picture of social class ecology, individual differences and subjective experience of status in a given situation independently contributing to the propensity for wise reasoning.

The current work adds nuance to the research on group differences in reasoning. Past research has demonstrated that wise reasoning style can occur independently from abstract cognitive abilities [18,60]. Thus, while higher-class individuals may enjoy the cognitive benefits of status (e.g. environments that foster development in such areas as fluid cognition), those same environments may constrain their ability or motivation to reason wisely (e.g. acknowledge change, uncertainty, and the limits of their knowledge). Conversely, limited resources and other threats associated with lower class environments may promote wise reasoning about interpersonal affairs, enabling greater vigilance and management of uncertainty associated with such environments.

Wise reasoning is domain-specific [14]. The present evidence of social class differences in wise reasoning chiefly concerns the domain of interpersonal conflicts, with little evidence for class-related differences in the domain of intergroup conflicts. This specificity of wise reasoning effects is consistent with the specialization hypothesis in ecological and evolutionary psychology [30], which poses specificity of ecologically bound adaptation to the domains critical for one's survival. Because intergroup conflicts in foreign countries are not impactful for lower-class Americans' for day-to-day activities, one can speculate that there is little ecological pressure for these individuals to develop a distinct reasoning style in that domain. The domain-specificity of the relationship between social class and wise reasoning opens an important avenue for future research.

The present results extend other scholarship on social class in the behavioural sciences. Some recent work has indicated that, in North American samples, higher class can be associated with less prosocial behaviour [33,61] and more antisocial outcomes in interpersonal and organization settings [62,63]. However, studies conducted in non-North American parts of the world have failed to yield similar results [64,65]. It is possible that a consideration of baseline sample differences in wise reasoning [66,67] may shed new light on these inconsistencies. Wise reasoning has been previously associated with prosocial tendencies [15,17,21], suggesting that differences in wise reasoning style may underlie or moderate class-related differences in interpersonal outcomes. Indeed, in the present Study 2 we observed that the effect of class-related level of education on wise reasoning was pronounced among young and middle-aged cohorts, but not older cohorts (see electronic supplementary material). Given that the older cohort showed a higher wise reasoning baseline in Study 2 [56], this observation dovetails with the broad speculation about the role of cohort/cultural effects when evaluating the relationship between class and prosociality.

A few caveats are in order before concluding. The operationalization of wise reasoning in the present research focused on situation-specific assessment of reasoning. The approach used in Study 1 enabled us to perform ecologically sensitive, large-scale analysis of social class differences across regions, individual differences, and situations. The standardized interview and content-analysis approach in Study 2 enabled us to ensure comparability of the situation people engaged in, and to examine behavioural, open-ended performance in the lab. However, these techniques do not assess performance on wise reasoning with the fine-grained precision common to standardized scholastic aptitude tests, nor do they enable equivocal assessment of latent abilities [14]. As with most individual differences, multi-iteration assessment is necessary for a fuller understanding of underlying traits [68]. Future research may help to design multi-iterative ecological and in-lab wise reasoning tasks, to supplement the present methods by identifying specific boundary conditions influencing wise reasoning performance.

Other key questions for future research concern possible ways to accommodate the concurrent development of domain-general cognitive abilities and wise reasoning, as well as identification of situations in which domain-general versus wise cognitive style may be more adaptive. It is possible that domain-general cognition may be preferred in well-defined contexts, whereas wise reasoning style may be preferred in ill-defined contexts [14,69], with the latter contexts probably more common for the working class individuals [23,28]. Finally, the failure of the middle-class educational system to successfully teach for wise reasoning about day-to-day interpersonal matters raises questions how school curricula can be improved [70].

## Supplementary Material

Supplementary Information Files

## Supplementary Material

Analysis code

## Data accessibility

Data and analyses are available on Open Science Framework (https://osf.io/xk8gp) [71].
