# Supplementary material for: Social class and wise reasoning about interpersonal conflicts across regions, persons and situations
Source: Proc Biol Sci. 2017 Dec 20;284(1869):20171870. doi: 10.1098/rspb.2017.1870 (PMC5745406; doi:10.1098/rspb.2017.1870)
Supplement: Supplementary Information Files [file rspb20171870supp1.pdf]

SUPPLEMENTARY INFORMATION FILES

for

Social class and wise reasoning about interpersonal conflicts across regions, persons and situations

Justin P. Brienza[1] & Igor Grossmann[2]*

University of Waterloo, Canada

Author Note

[1]Lazaridis School of Business and Economics 64 University Avenue West, Waterloo, Ontario, Canada N2L 3C7

[2]Department of Psychology, University of Waterloo, Canada, 200 University Avenue West, Waterloo, Ontario, Canada N2L 3G1

* Igor Grossmann, 200 University Avenue West, Waterloo, Ontario, Canada N2L 3G1, Tel. +1 519 888-4567 ext. 31793, e-mail: igrossma@uwaterloo.ca

Study 1: Methods for control variables

We obtained State-level population estimates for 2014 from IPUMS, and State-level 2014-2015 estimates of scholastic aptitude (SAT) from the College Board (research.collegeboard.org). To assess income inequality in each U.S. state, we gathered Gini coefficients from the American Community Survey for the year when our study was conducted (2014-2015). Further, we obtained values for the percentage of the population living in urban clusters from the 2010 Census, conducted by the US Census Bureau.

To control for individual and state-specific differences in social desirability, participants (n = 637) completed Paulhus' 40-item scale of social desirability [1]. We performed analyses on the total social desirability score, which was computed as the sum of socially desirable statements participants endorsed strongly (i.e., > 5 on a 7-point scale) about themselves (Cronbach's α = 90). Because of possible social class differences in prosocial tendencies and possible conceptual overlap with other individual differences, a subset of participants (n = 833) completed two facets of the Big Five personality traits – agreeableness and openness using an established inventory [2], which showed excellent reliability (Cronbach's αs ≤ .82). To control for the potential confounding role of self-other orientation, a subset of participants (n = 650) completed measures of attention to personal and others' emotions, which also showed excellent reliability (Cronbach's αs ≤ .88).

Study 2: Education effects by age cohort

Given prior reports that older cohorts may be more likely to reason wisely about such interpersonal conflicts than their younger and middle-aged counterparts [3], we performed a parallel set of analyses separately by age group (25-40, 41-59, 60-90). The results indicated significant effects of education for younger, $F(3,63) = 3.48$, $P = 0.021$, $\eta_p^2 = .142$, and middle-aged adults, $F(3,55) = 2.17$, $P = 0.102$, $\eta_p^2 = .106$, but not for older adults, $F(3,57) = .552$, *ns.* Post-hoc analyses indicated that younger and middle-aged participants without college education scored half standard deviation higher than their more educated counterparts, 25-40 years: B = .491, SE = .223, 95% CI [.045, .936], P = 0.031; 41-59 years: B = .429, SE = .200, 95% CI [.028, .830], P = 0.037.

Table S1. *Demographics in Study 1*

N	2,145
Age_{mean} (*SD*)	32.75 (10.77)
Gender (%f/m)	59.3/40.7
Income (%)	
Under $15,000	12.7
$15,001-$25,000	14.0
$25,001-$35,000	16.8
$35,001-$50,000	17.0
$50,001-$75,000	18.7
$75,001-$100,000	12.3
$100,001-$150,000	6.8
Over $150,000	1.6
Education (%)	
Some high school	1.1
High school or equivalent	10.5
Some college	31.1
College degree	29.4
Undergraduate degree	14.4
Graduate degree	13.5

Table S2.
Factor Analysis of the 21 Wise Reasoning Items.

Item #	Component				
	1	2	3	4	5
1			**.719**		
2	.339		**.415**		
3			**.923**		
4	.208		**.524**		
5	.533			.182	
6	.387			**.432**	
7	-.151			**1.024**	
8				**.613**	.259
9					**.844**
10					**.624**
11	.190				**.440**
12	.215				**.382**
13	**.814**			-.117	
14	**.939**			-.147	
15	**.745**				.104
16	**.693**		-.111		
17	**.678**				
18		**.821**			
19		**.839**			
20		**.952**			
21		**.760**			.123

Note: Bolded coefficients represent a priori dimension loadings

Table S3.

Model Fit Indices for the 5-Factor model of Wise Reasoning Style.

$X^2(df)$	*P*	AIC	BIC	CFI	TLI	RMSEA[90% CI]
1399.23(286)	< .001	166611.66	167127.71	.951	.945	.043[.040, .045]

Note. Results are based on maximum-likelihood estimation. X^2 = Satorra-Bentler-scaled X^2.

Table S4.

Effect of State-, Individual-, and Situation-Level Social Class on Wise Reasoning about Interpersonal Conflicts.

**Social Status**	***B***	***SE***	***t*-value**	***P***
State-Level				
Wise Reasoning Index	-.161	.045	-3.583	< .001
Intellectual Humility	-.185	.058	-3.192	.001
Change	-.191	.060	-3.162	.002
Outsider Viewpoint	-.184	.074	-2.470	.014
Perspectives	-.168	.052	-3.219	.001
Compromise/Resolution	-.186	.061	-3.024	.003
Individual-Level				
Wise Reasoning Index	-.219	.045	-4.910	< .001
Intellectual Humility	-.231	.057	-4.103	< .001
Change	-.312	.060	-5.230	< .001
Outsider Viewpoint	-.212	.074	-2.873	.004
Perspectives	-.227	.052	-4.376	< .001
Compromise/Resolution	-.261	.061	-4.297	< .001
Situation-Level				
Wise Reasoning Index	-.063	.020	-4.525	<.001
Intellectual Humility	-.075	.024	-3.100	.002
Change	-.099	.030	-3.293	.001
Outsider Viewpoint	-.078	.031	-2.561	.011
Perspectives	-.070	.023	-3.051	.002
Compromise/Resolution	-.079	.027	-2.983	.003

Note. State- and individual-levels of analyses represent fixed effects from random intercept linear mixed effects models with maximum likelihood *t*-tests and Satterthwaite approximations to degrees of freedom. Situation-level effects represent linear regression analyses. State-/individual-level *df* = 2145, Situation-level *df* = 728.

Table S5.

Mediation Model Testing the Effect of Situation-Level (Subjective) Social Class and Perceived Interpersonal Closeness on Wise Reasoning Style, with 95% Quasi-Bayesian Confidence Intervals (CI)

	B	***CI* Lower**	***CI* Upper**	***P***
Wise Reasoning Index				
Indirect Effect	-.014	-.028	-.002	0.020
Direct Effect	-.087	-.149	-.024	0.010
Total Effect	-.101	-.163	-.038	< 0.001
Proportion Mediated (%)	13.24			
Intellectual Humility				
Indirect Effect	-.010	-.022	-.001	0.030
Direct Effect	-.070	-.121	-.020	< 0.001
Total Effect	-.081	-.131	-.030	< 0.001
Proportion Mediated (%)	12.37			
Change				
Indirect Effect	-.009	-.018	-.001	0.020
Direct Effect	-.070	-.123	-.018	0.010
Total Effect	-.079	-.132	-.026	< 0.001
Proportion Mediated (%)	10.66			
Outsider Viewpoint				
Indirect Effect	-.005	-.013	.0002	0.070
Direct Effect	-.073	-.131	-.015	0.010
Total Effect	-.078	-.136	-.021	0.010
Proportion Mediated (%)	5.78			
Perspectives				
Indirect Effect	-.010	-.021	-.001	0.030
Direct Effect	-.059	-.105	-.016	0.010
Total Effect	-.069	-.115	-.025	< 0.001
Proportion Mediated (%)	14.35			
Compromise/Resolution				
Indirect Effect	-.011	-.023	-.001	0.030
Direct Effect	-.066	-.119	-.015	0.010
Total Effect	-.077	-.129	-.026	< 0.001
Proportion Mediated (%)	14.37			

Notes: $N = 730$.

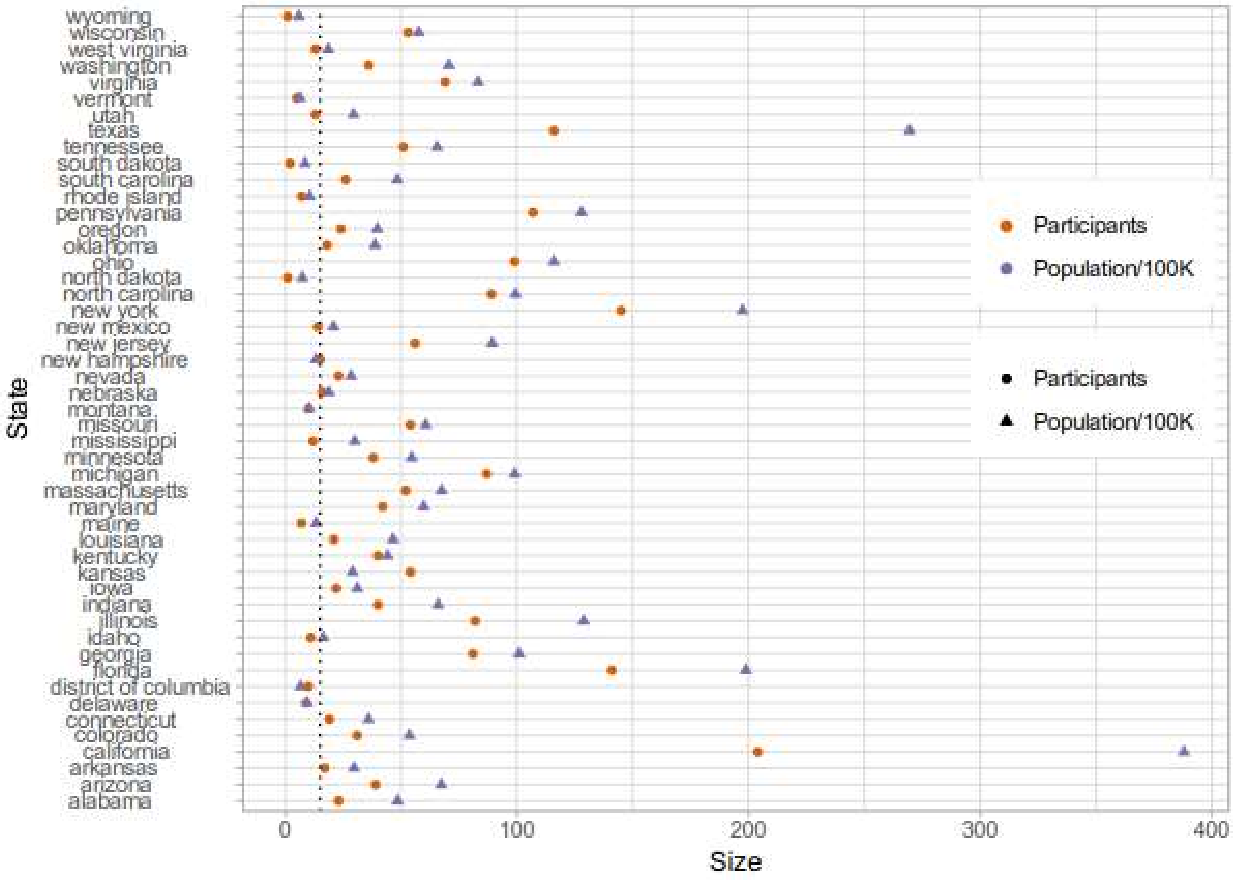

Figure S1. Distribution of study participants by U.S. State and size of each respective state. Dotted line represents $n = 15$.

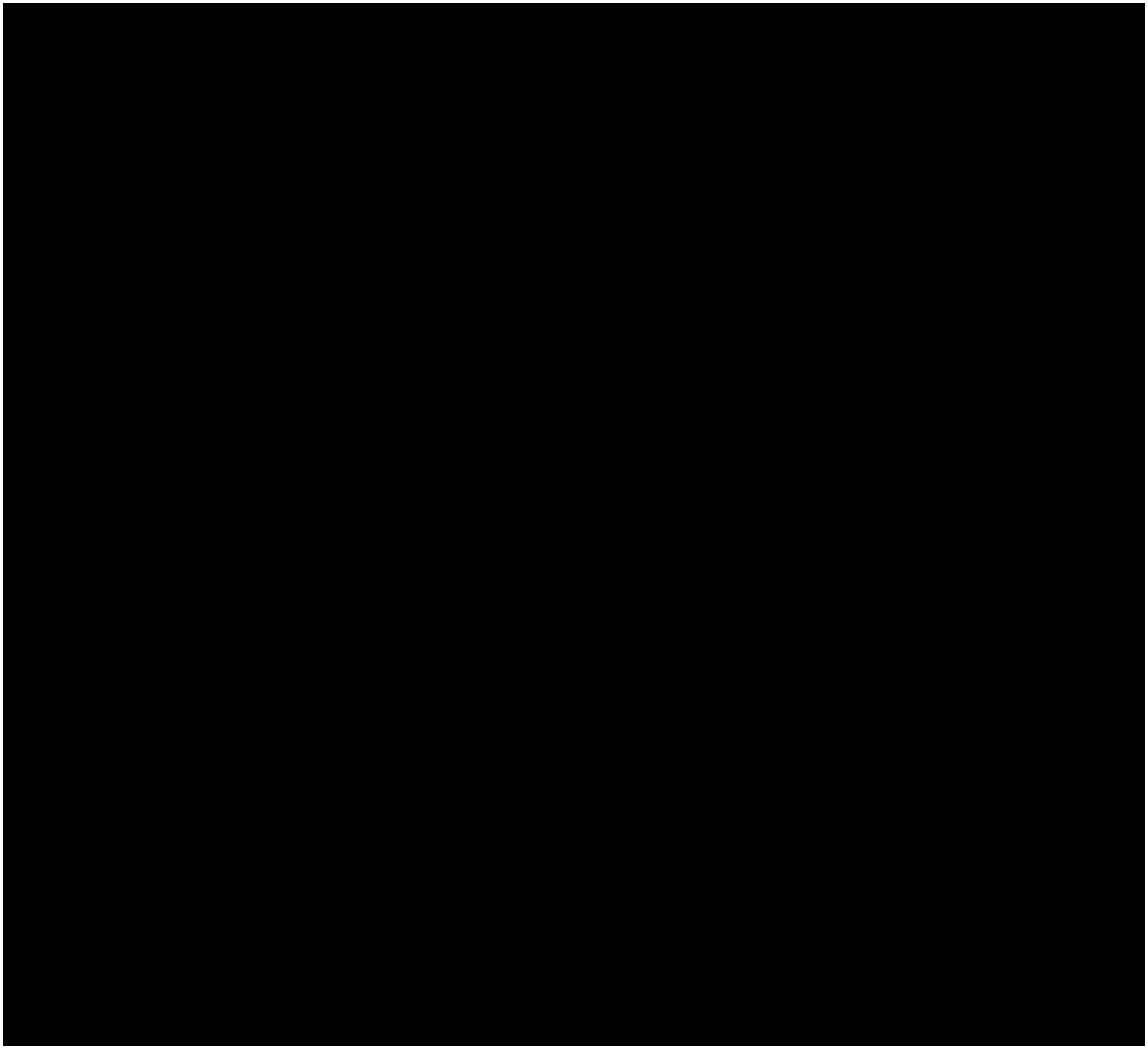

Figure S2. Final 5-Factor model of wise reasoning with standardized coefficients.

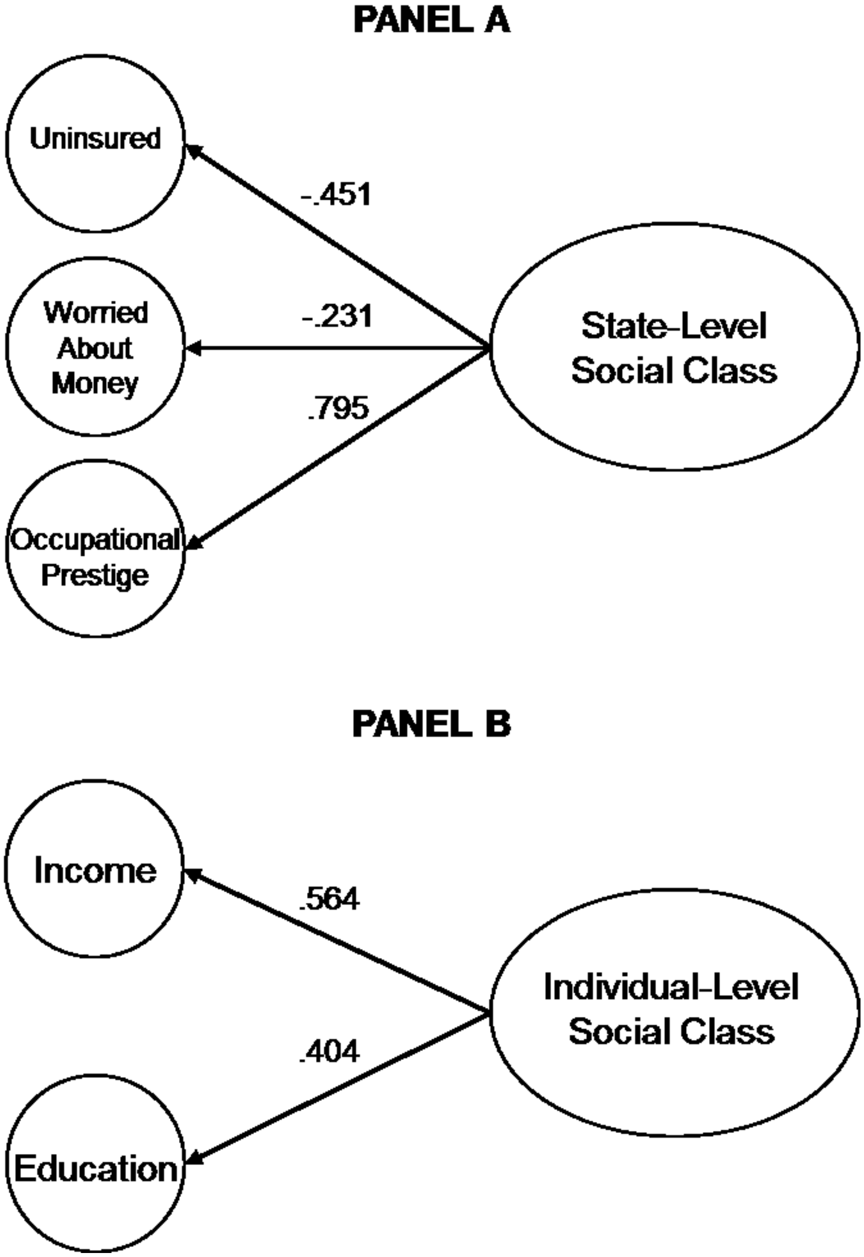

Figure S3. Panel A. 3-Factor model of state-level social class with standardized coefficients.
Panel B. 2-Factor model of individual-level social class with standardized coefficients.

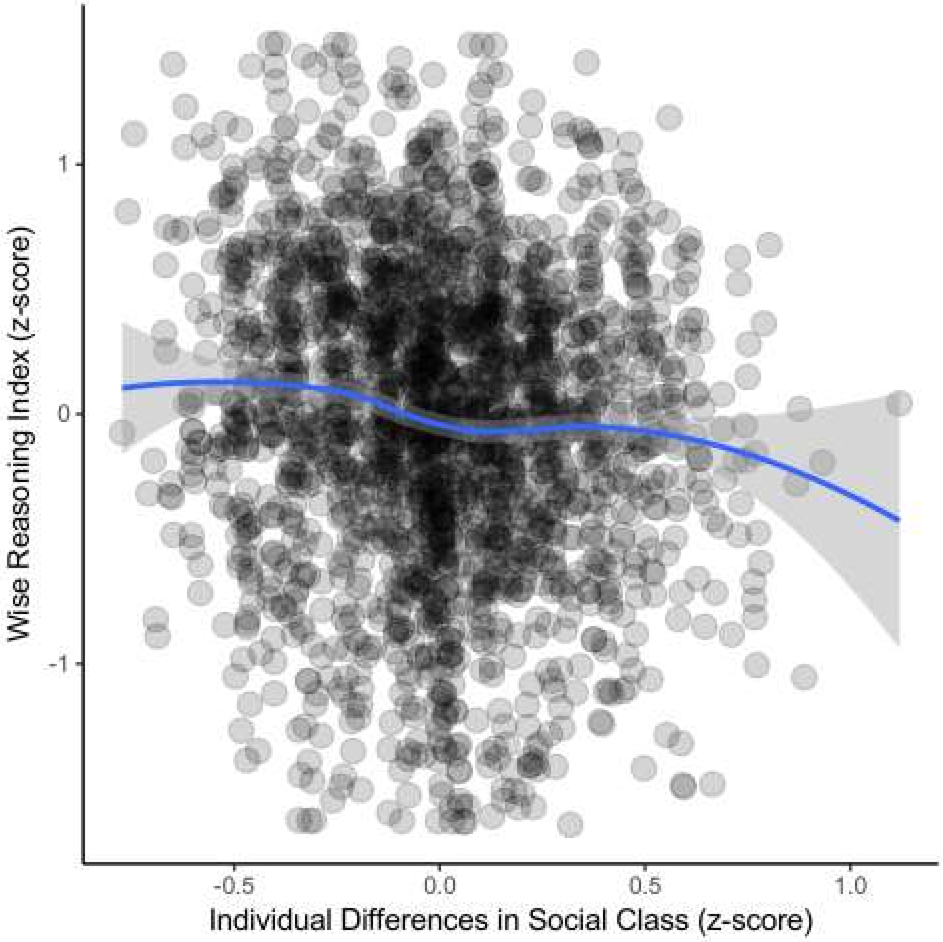

Figure S4. Individuals with higher social class showed less wise reasoning about interpersonal conflicts. Scatterplot with the line of best fit based on loess smoothness estimation.

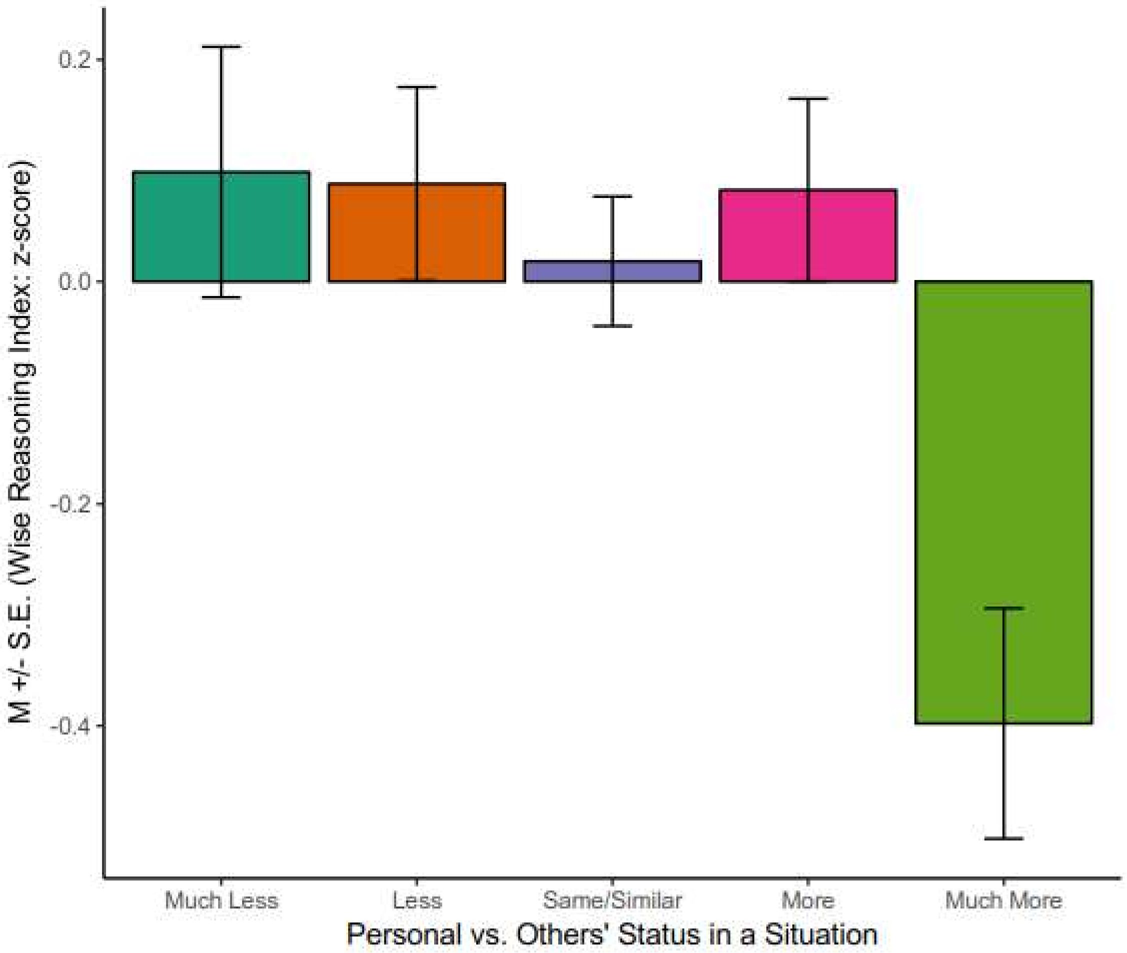

Figure S5. Means and standard errors of wise reasoning about interpersonal conflicts as a function of personal and other person's status in the conflict situation in Study 1.

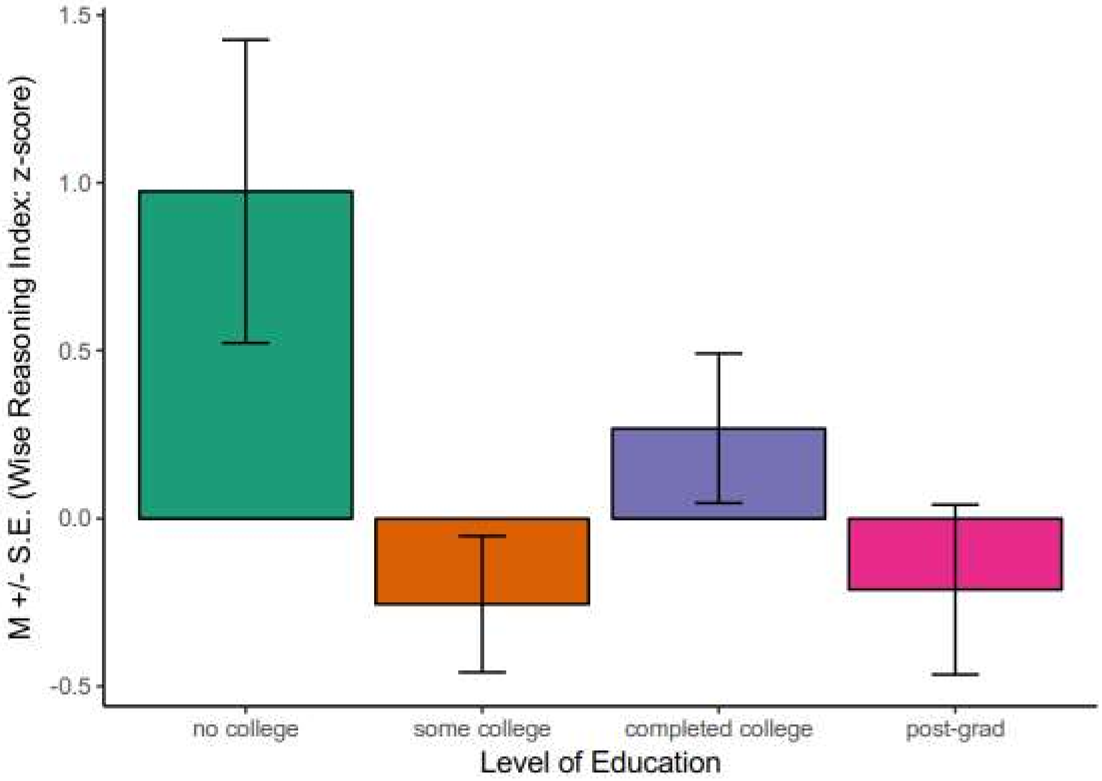

Figure S6. Means and standard errors of wise reasoning about interpersonal conflicts as a function of level of education in Study 2.

Appendix S1: Event Reconstruction Method for Wise Reasoning [4]

In this section, we would like you to think about a difficult situation that has happened to you with another person (e.g., a disagreement, conflict), **specifically in your workplace** / **specifically with a close friend**. This should be a situation that you were involved in, whether or not you were the person who initiated the situation. We would like you to take a moment to recall the situation and visualize the events in your mind's eye; consider who was involved and what happened, what you thought and how you felt. After doing so, please respond to the following questions:

1. When did this situation first begin?
    i. This week
    ii. Within the last month
    iii. Within the last 6 months
    iv. Within the last year
    v. Over a year ago
2. What day of the week was it?
    i. M
    ii. T
    iii. W
    iv. T
    v. F
    vi. S
    vii. S
    viii. Don't remember
3. What time of day was it?
    i. Morning
    ii. Afternoon
    iii. Evening
    iv. Don't remember
4. What were you doing when it happened? This only needs to be a sentence or two.
    i. {text box}
5. Where were you?
    i. {text box}
    ii.
6. As you were thinking about this situation, what thoughts came to your mind? Please write your thoughts in the space provided.
    i. {text box}

Wise Reasoning Items

Please continue to think about the situation you called to mind in the previous section and recall the extent to which you engaged in the following thoughts and behaviours – what you actually did as the situation unfolded. None of the statements listed below are supposed to be "good" or "bad." We are simply interested in how people approach difficult situations. Therefore, it is very important to us that you answer as accurately as possible - your honesty is appreciated, and your replies are, of course, anonymous.

"While this situation was unfolding, I did the following..." (from 1 – *not at all*, to 5 – *very much*)

1. Double-checked whether my opinion on the situation might be incorrect
2. Double-checked whether the other person's opinions might be correct
3. Looked for any extraordinary circumstances before forming my opinion
4. Behaved as if there may be some information to which I did not have access
5. Looked for different solutions as the situation evolved
6. Considered alternative solutions as the situation evolved
7. Believed the situation could lead to a number of different outcomes
8. Thought the situation could unfold in many different ways
9. Wondered what I would think if I was somebody else watching the situation
10. Tried to see the conflict from the point of view of an uninvolved person
11. Asked myself what other people might think or feel if they were watching the conflict
12. Thought about whether an outside person might have a different opinion from mine about the situation
13. Put myself in the other person's shoes
14. Tried to communicate with the other person what we might have in common
15. Made an effort to take the other person's perspective
16. Took time to get the other person's opinions on the matter before coming to a conclusion
17. Tried my best to find a way to accommodate both of us
18. Though it may not have been possible, I searched for a solution that could result in both of us being satisfied
19. Considered first whether a compromise was possible in resolving the situation
20. Viewed it as very important that we resolve the situation
21. Tried to anticipate how the conflict might be resolved

Legend

Items 1-4: intellectual humility/recognition of limits of knowledge; items 5-8: consideration of change and multiple ways situation may unfold; items 9-12: view of the event through the vantage point of an outsider; items 13-16: others' perspectives; items 17-21: search for a compromise/conflict resolution.

References

1. Paulhus, D. L. 1988 Balanced inventory of desirable responding (BIDR). *Accept. Commit. Ther. Meas. Packag.* **41**.
2. John, O. P., Naumann, L. P. & Soto, C. J. 2008 Paradigm shift to the integrative big give trait taxonomy. In *Handbook of personality: Theory and research* (eds O. P. John R. W. Robins & L. A. Pervin), pp. 114–158. New York, NY: Guilford Press.
3. Grossmann, I., Na, J., Varnum, M. E. W., Park, D. C., Kitayama, S. & Nisbett, R. E. 2010 Reasoning about social conflicts improves into old age. *Proc. Natl. Acad. Sci. U. S. A.* **107**. (doi:10.1073/pnas.1001715107)
4. Brienza, J., Kung, F. Y. H., Santos, H. C., Bobocel, R. & Grossmann, I. 2017 Wisdom, bias, and balance: Toward a process-sensitive measurement of wisdom-related cognition. *J. Pers. Soc. Psychol.* (doi:10.1037/pspp0000171)
