# Supplementary material for: Social class and wise reasoning about interpersonal conflicts across regions, persons and situations
Source: Proc Biol Sci. 2017 Dec 20;284(1869):20171870. doi: 10.1098/rspb.2017.1870 (PMC5745406; doi:10.1098/rspb.2017.1870)

Wise Reasoning and Social Class Analyses

Igor Grossmann

Aug 20, 2017

```r
require(foreign)
```

```
## Loading required package: foreign
```

```
## Warning: package 'foreign' was built under R version 3.3.3
```

```r
library(psych)
```

```
## Warning: package 'psych' was built under R version 3.3.3
```

```r
library(ggplot2)
```

```
## Warning: package 'ggplot2' was built under R version 3.3.3
```

```
##
## Attaching package: 'ggplot2'
```

```
## The following objects are masked from 'package:psych':
##
##     %+%, alpha
```

```r
library(lme4)
```

```
## Warning: package 'lme4' was built under R version 3.3.3
```

```
## Loading required package: Matrix
```

```r
library(lmerTest)
```

```
## Warning: package 'lmerTest' was built under R version 3.3.3
```

```
##
## Attaching package: 'lmerTest'
```

```
## The following object is masked from 'package:lme4':
##
##     lmer
```

```
## The following object is masked from 'package:stats':
##
##     step
```

```
library(lavaan)
```

```
## Warning: package 'lavaan' was built under R version 3.3.3
```

```
## This is lavaan 0.5-23.1097
```

```
## lavaan is BETA software! Please report any bugs.
```

```
##
## Attaching package: 'lavaan'
```

```
## The following object is masked from 'package:psych':
##
##     cor2cov
```

```
library(plyr)
```

```
## Warning: package 'plyr' was built under R version 3.3.3
```

```
library(dplyr)
```

```
## Warning: package 'dplyr' was built under R version 3.3.3
```

```
##
## Attaching package: 'dplyr'
```

```
## The following objects are masked from 'package:plyr':
##
##     arrange, count, desc, failwith, id, mutate, rename, summarise,
##     summarize
```

```
## The following objects are masked from 'package:stats':
##
##     filter, lag
```

```
## The following objects are masked from 'package:base':
## 
##     intersect, setdiff, setequal, union
```

```
library(apaTables)
library(QuantPsyc)
```

```
## Warning: package 'QuantPsyc' was built under R version 3.3.3
```

```
## Loading required package: boot
```

```
## Warning: package 'boot' was built under R version 3.3.3
```

```
## 
## Attaching package: 'boot'
```

```
## The following object is masked from 'package:psych':
## 
##     logit
```

```
## Loading required package: MASS
```

```
## Warning: package 'MASS' was built under R version 3.3.3
```

```
## 
## Attaching package: 'MASS'
```

```
## The following object is masked from 'package:dplyr':
## 
##     select
```

```
## 
## Attaching package: 'QuantPsyc'
```

```
## The following object is masked from 'package:Matrix':
## 
##     norm
```

```
## The following object is masked from 'package:base':
## 
##     norm
```

```
library(reshape2)
```

```
## Warning: package 'reshape2' was built under R version 3.3.3
```

```
library(mediation)
```

```
## Loading required package: mvtnorm
```

```
## Loading required package: sandwich
```

```
## mediation: Causal Mediation Analysis
## Version: 4.4.5
```

```
##
## Attaching package: 'mediation'
```

```
## The following object is masked from 'package:psych':
##
##     mediate
```

```
library(lsmeans)
```

```
## Warning: package 'lsmeans' was built under R version 3.3.3
```

```
## Loading required package: estimability
```

```
##
## Attaching package: 'lsmeans'
```

```
## The following object is masked from 'package:lmerTest':
##
##     lsmeans
```

```
options(scipen=999)
#read in the correct file (only first 7 samples, with the same presentation order: wisdom comes
 first)
mrb<-read.spss(choose.files(), to.data.frame=TRUE, use.value.labels=F)
```

```
## Warning in read.spss(choose.files(), to.data.frame = TRUE, use.value.labels
## = F): C:\Users\igrossma\Google Drive\Drive\scales\social class\7
## samples.wisdom first order.apr18.2017.abridged.sav: Unrecognized record
## type 7, subtype 14 encountered in system file
```

```
## Warning in read.spss(choose.files(), to.data.frame = TRUE, use.value.labels
## = F): C:\Users\igrossma\Google Drive\Drive\scales\social class\7
## samples.wisdom first order.apr18.2017.abridged.sav: Unrecognized record
## type 7, subtype 17 encountered in system file
```

```
## Warning in read.spss(choose.files(), to.data.frame = TRUE, use.value.labels
## = F): C:\Users\igrossma\Google Drive\Drive\scales\social class\7
## samples.wisdom first order.apr18.2017.abridged.sav: Unrecognized record
## type 7, subtype 18 encountered in system file
```

select subsamples that: - only completed wise reasoning measure first (no possible carry-over effects), samples >7 have manipulation, hence excluded - did not repeat the study (based on duplicate check (based on IPs))

```
mrbclean <- mrb[ which(mrb$presentationorder=='1' &
                         mrb$sampleIG <8 & mrb$filter.dup==0) , ]
mrbclean$SAT<-((mrbclean$SAT2014)+(mrbclean$SAT2015))/2
#calculate SAT scores across two years in whcih study was conducted
mrbclean1 <- subset(mrbclean, filter.dup=="0"|filter.dup=="NA",
select=c(w1,w2,w3,w4,w5,w6,w7,w8,w9,w10,w11,w12,w13,w14,w15,w16,w17,w18,w19,w20,w21,
WorriedMoney, Uninsured,Edu,Income,Gender,OccPres, Age, Population,
status, State, closebefor, BIDRFull, EIOthersEmotions , EIEmotionReg,SAT ))
#subset data for fitting SEM model
mrbshort <- mrbclean1[c(1:25,27)] #subset data for fitting SEM model
```

Examine model fit of wise reasoning and social class latent factors .

FIRST, EXPLORATORY ANALYSIS OF SOCIAL STATUS (just for information)

```
factanal(~WorriedMoney+Uninsured+OccPres+Edu+log(Income), factors=2,
         data=mrbclean1, rotation="Promax")
```

```
##
## Call:
## factanal(x = ~WorriedMoney + Uninsured + OccPres + Edu + log(Income),     factors = 2, data = mrbclean1, rotation = "Promax")
##
## Uniquenesses:
## WorriedMoney    Uninsured      OccPres          Edu  log(Income)
##        0.952        0.827        0.372        0.005        0.944
##
## Loadings:
##              Factor1 Factor2
## WorriedMoney         -0.219
## Uninsured            -0.414
## OccPres               0.797
## Edu           1.002
## log(Income)   0.205
##
##                Factor1 Factor2
## SS loadings      1.047   0.863
## Proportion Var   0.209   0.173
## Cumulative Var   0.209   0.382
##
## Factor Correlations:
##         Factor1 Factor2
## Factor1   1.000  -0.149
## Factor2  -0.149   1.000
##
## Test of the hypothesis that 2 factors are sufficient.
## The chi square statistic is 1.11 on 1 degree of freedom.
## The p-value is 0.293
```

==> 2 factors seem appropriate and structure into state-level and individual factors. # #EXPLORATORY ANALYSIS OF WISE REASONING

```
#results suggest a consistent pattern with apriori hypothesized results. One notable exception,
#item w8 appears to belong to humility rather than change
factanal(~w1+w2+w3+w4+w5+w6+w7+w8+w9+w10+w11+w12+w13+w14+w15+w16+w17+w18
         +w19+w20+w21, factors=5, data=mrbshort, rotation="Promax")
```

```
##
## Call:
## factanal(x = ~w1 + w2 + w3 + w4 + w5 + w6 + w7 + w8 + w9 + w10 +     w11 + w12 + w13 + w14 +
 w15 + w16 + w17 + w18 + w19 + w20 +     w21, factors = 5, data = mrbshort, rotation = "Promax")
##
## Uniquenesses:
##    w1    w2    w3    w4    w5    w6    w7    w8    w9   w10   w11   w12
## 0.418 0.593 0.175 0.456 0.541 0.526 0.166 0.501 0.369 0.689 0.555 0.719
##   w13   w14   w15   w16   w17   w18   w19   w20   w21
## 0.396 0.326 0.424 0.646 0.578 0.366 0.301 0.207 0.360
##
## Loadings:
##     Factor1 Factor2 Factor3 Factor4 Factor5
## w1                   0.719
## w2   0.339           0.415
## w3                   0.923
## w4   0.208           0.524
## w5   0.533                   0.182
## w6   0.387                   0.432
## w7  -0.151                   1.024
## w8                           0.613   0.259
## w9                                   0.844
## w10                                  0.624
## w11  0.190                           0.440
## w12  0.215                           0.382
## w13  0.814                  -0.117
## w14  0.939                  -0.147
## w15  0.745                           0.104
## w16  0.693          -0.111
## w17  0.678
## w18          0.821
## w19          0.839
## w20          0.952
## w21          0.760                   0.123
##
##                Factor1 Factor2 Factor3 Factor4 Factor5
## SS loadings      3.753   2.894   1.853   1.703   1.588
## Proportion Var   0.179   0.138   0.088   0.081   0.076
## Cumulative Var   0.179   0.317   0.405   0.486   0.561
##
## Factor Correlations:
##         Factor1 Factor2 Factor3 Factor4 Factor5
## Factor1   1.000  -0.427  -0.590  -0.692   0.653
## Factor2  -0.427   1.000   0.373   0.406  -0.534
## Factor3  -0.590   0.373   1.000   0.351  -0.473
## Factor4  -0.692   0.406   0.351   1.000  -0.632
## Factor5   0.653  -0.534  -0.473  -0.632   1.000
##
## Test of the hypothesis that 5 factors are sufficient.
## The chi square statistic is 1468.47 on 115 degrees of freedom.
## The p-value is 0.0000000000000000000000000000000000000000000000000000000000000000000000000000
0000000000000000000000000000000000000000000000000000000000000000000000000000000000000000000000000
0000000000000000000000000000000000000000000000000000000225
```

```
#overall, 5 model wise reasoning construct looks good
```

TEST STRUCTURAL MODELS OF WISE REASONING.

model with wisdom-related items feeding into 5 factors, which in turn feed into a second-order factor also, SES at state and individual levels

```
w.model<-
  'persp =~ w1+ w2+ w3+ w4
change=~w5+w6+w7+w8
humility=~w9+ w10+ w11+ w12
comprres=~w13+ w14+ w15+w16+w17
outsider=~w18+w19+ w20+ w21
wow=~persp+change+humility+comprres+outsider
class=~WorriedMoney+Uninsured+OccPres
pclass=~Edu+Income'


fit <- cfa(w.model, data = mrbshort, missing = "ML")
fitMeasures(fit,  c("chisq", "df", "pvalue", "cfi", "tli","aic","bic",
                    "rmsea","rmsea.ci.lower","rmsea.ci.upper"))
```

```
##          chisq             df         pvalue            cfi            tli
##       2657.277        291.000          0.000          0.896          0.884
##            aic            bic          rmsea rmsea.ci.lower rmsea.ci.upper
##     167859.714     168347.411          0.062          0.059          0.064
```

```
#very decent model with acceptable indices.
```

IMRPOVE MODEL FIT WITH MODIFICATION INDICES

```
modindices(fit, power=T, sort.=T) #suggestion to correlate residuals
```

```
##               lhs op          rhs      mi    epc sepc.all delta    ncp
## 555           w16 ~~          w17 528.463  0.462    0.348   0.1 24.758
## 420            w7 ~~           w8 468.119  0.505    0.355   0.1 18.334
## 380            w5 ~~           w7 226.364 -0.333   -0.236   0.1 20.451
## 519           w13 ~~          w14 174.171  0.237    0.160   0.1 31.045
## 401            w6 ~~           w8 166.769 -0.286   -0.202   0.1 20.356
## 286            w1 ~~           w3 131.415  0.199    0.151   0.1 33.200
## 379            w5 ~~           w6 126.049  0.294    0.209   0.1 14.631
## 173      comprres =~           w5 106.307  0.389    0.310   0.1  7.021
## 457            w9 ~~          w10  95.960  0.288    0.164   0.1 11.591
## 439            w8 ~~           w9  92.088  0.217    0.143   0.1 19.599
## 522           w13 ~~          w17  89.008 -0.161   -0.122   0.1 34.167
## 216           wow =~           w5  84.983  0.639    0.380   0.1  2.084
## 624      humility ~~     outsider  74.262  0.152    0.163   0.1 32.127
## 154      humility =~           w8  66.291  0.361    0.263   0.1  5.074
## 628      comprres ~~     outsider  63.326 -0.137   -0.136   0.1 33.910
## 381            w5 ~~           w8  63.079 -0.177   -0.124   0.1 20.234
## 105         persp =~           w7  58.984 -0.310   -0.222   0.1  6.135
## 170      comprres =~           w2  58.651  0.314    0.245   0.1  5.958
## 218           wow =~           w7  55.170 -0.490   -0.292   0.1  2.302
## 137        change =~          w17  54.401  0.256    0.215   0.1  8.277
## 152      humility =~           w6  53.758 -0.309   -0.229   0.1  5.647
## 618        change ~~     comprres  52.537  0.116    0.133   0.1 39.175
## 217           wow =~           w6  50.963 -0.498   -0.298   0.1  2.057
## 400            w6 ~~           w7  50.391  0.157    0.112   0.1 20.414
## 203      outsider =~          w14  49.817 -0.140   -0.122   0.1 25.244
## 103         persp =~           w5  49.710  0.280    0.200   0.1  6.345
## 231           wow =~          w20  46.241 -0.221   -0.118   0.1  9.443
## 214           wow =~           w3  44.461 -0.530   -0.322   0.1  1.582
## 183      comprres =~          w20  44.026 -0.148   -0.106   0.1 20.018
## 175      comprres =~           w7  43.795 -0.250   -0.199   0.1  7.017
## 521           w13 ~~          w16  43.170 -0.127   -0.087   0.1 26.733
## 104         persp =~           w6  40.465 -0.252   -0.181   0.1  6.371
## 118         persp =~          w20  40.002 -0.156   -0.099   0.1 16.448
## 534           w14 ~~          w17  39.579 -0.106   -0.079   0.1 35.181
## 215           wow =~           w4  38.131  0.483    0.287   0.1  1.636
## 197      outsider =~           w8  37.802  0.153    0.135   0.1 16.226
## 163      humility =~          w21  37.692  0.170    0.113   0.1 13.094
## 162      humility =~          w20  36.555 -0.153   -0.101   0.1 15.644
## 610         persp ~~       change  36.404 -0.085   -0.108   0.1 50.755
## 225           wow =~          w14  35.403 -0.559   -0.323   0.1  1.131
## 127        change =~           w3  34.575 -0.178   -0.141   0.1 10.965
## 125        change =~           w1  34.263 -0.183   -0.150   0.1 10.203
## 219           wow =~           w8  32.207  0.386    0.227   0.1  2.163
## 213           wow =~           w2  30.612  0.464    0.270   0.1  1.425
## 576           w18 ~~          w21  30.429 -0.107   -0.061   0.1 26.778
## 172      comprres =~           w4  30.232  0.206    0.164   0.1  7.103
## 533           w14 ~~          w16  30.231 -0.104   -0.070   0.1 27.763
## 451            w8 ~~          w21  29.936  0.108    0.068   0.1 25.809
## 623      humility ~~     comprres  29.249 -0.089   -0.108   0.1 36.587
## 156      humility =~          w14  28.941 -0.239   -0.171   0.1  5.087
## 490           w11 ~~          w12  27.100  0.124    0.087   0.1 17.693
## 287            w1 ~~           w4  26.821 -0.088   -0.065   0.1 34.627
```

```
## 200     outsider =~           w11  26.741  0.116    0.107   0.1  19.783
## 285           w1 ~~            w2  26.517 -0.094   -0.068   0.1  30.321
## 153     humility =~            w7  25.715 -0.217   -0.160   0.1   5.480
## 128       change =~            w4  25.167  0.165    0.128   0.1   9.246
## 171     comprres =~            w3  24.062 -0.178   -0.145   0.1   7.578
## 458           w9 ~~           w11  23.594 -0.127   -0.087   0.1  14.590
## 232          wow =~           w21  22.121  0.166    0.090   0.1   7.996
## 589          w20 ~~           w21  21.747  0.094    0.054   0.1  24.831
## 141       change =~           w21  21.607  0.116    0.082   0.1  16.033
## 151     humility =~            w5  21.478  0.195    0.144   0.1   5.627
## 130       change =~           w10  19.962 -0.222   -0.147   0.1   4.043
## 221          wow =~           w10  19.622 -0.549   -0.278   0.1   0.651
## 206     outsider =~           w17  18.172  0.093    0.090   0.1  21.137
## 468           w9 ~~           w21  18.120  0.080    0.048   0.1  28.380
## 565          w17 ~~           w18  17.966  0.073    0.049   0.1  34.101
## 150     humility =~            w4  17.857  0.179    0.131   0.1   5.581
## 140       change =~           w20  17.761 -0.096   -0.066   0.1  19.378
## 178     comprres =~           w10  17.630 -0.244   -0.165   0.1   2.968
## 226          wow =~           w15  17.597  0.392    0.229   0.1   1.147
## 357           w4 ~~            w5  16.351  0.064    0.045   0.1  39.729
## 316           w2 ~~            w9  16.321 -0.085   -0.056   0.1  22.543
## 459           w9 ~~           w12  16.037 -0.103   -0.066   0.1  15.139
## 169     comprres =~            w1  15.818 -0.142   -0.119   0.1   7.869
## 290           w1 ~~            w7  15.634 -0.068   -0.051   0.1  33.435
## 126       change =~            w2  15.140  0.143    0.108   0.1   7.437
## 612        persp ~~      comprres  14.599  0.061    0.076   0.1  39.565
## 157     humility =~           w15  14.505  0.172    0.124   0.1   4.902
## 149     humility =~            w3  14.372 -0.153   -0.115   0.1   6.169
## 184     comprres =~           w21  14.089  0.092    0.066   0.1  16.788
## 134       change =~           w14  14.052 -0.124   -0.094   0.1   9.098
## 161     humility =~           w19  13.747  0.096    0.065   0.1  14.790
## 429           w7 ~~           w17  13.536  0.070    0.054   0.1  27.430
## 202     outsider =~           w13  13.266 -0.075   -0.066   0.1  23.715
## 117        persp =~           w19  13.158  0.092    0.060   0.1  15.531
## 461           w9 ~~           w14  12.913 -0.063   -0.041   0.1  32.851
## 198     outsider =~            w9  12.433  0.088    0.074   0.1  15.996
## 321           w2 ~~           w14  11.408  0.057    0.038   0.1  35.032
## 136       change =~           w16  11.017  0.131    0.100   0.1   6.383
## 174     comprres =~            w6  10.867 -0.124   -0.100   0.1   7.020
## 212          wow =~            w1  10.862 -0.244   -0.153   0.1   1.819
## 402           w6 ~~            w9  10.765 -0.061   -0.041   0.1  29.279
## 111        persp =~           w13  10.243  0.132    0.092   0.1   5.922
## 574          w18 ~~           w19   9.879  0.061    0.035   0.1  26.440
## 520          w13 ~~           w15   9.334 -0.054   -0.037   0.1  31.855
## 176     comprres =~            w8   9.219  0.119    0.094   0.1   6.519
## 582          w19 ~~           w20   8.961 -0.063   -0.036   0.1  22.396
## 195     outsider =~            w6   8.930 -0.062   -0.056   0.1  23.038
## 426           w7 ~~           w14   8.846 -0.051   -0.035   0.1  33.995
## 410           w6 ~~           w17   8.662  0.048    0.037   0.1  36.954
## 320           w2 ~~           w13   8.587  0.051    0.035   0.1  32.503
## 312           w2 ~~            w5   8.474  0.053    0.037   0.1  30.073
## 107        persp =~            w9   8.430  0.157    0.106   0.1   3.410
## 505          w12 ~~           w13   8.281  0.055    0.037   0.1  27.336
## 228          wow =~           w17   8.271  0.262    0.169   0.1   1.209
```

```
## 230          wow =~          w19   8.004  0.094    0.051   0.1   8.993
## 430           w7 ~~          w18   7.974  0.054    0.034   0.1  27.728
## 474          w10 ~~          w11   7.942 -0.076   -0.047   0.1  13.790
## 135       change =~          w15   7.877  0.096    0.073   0.1   8.547
## 536          w14 ~~          w19   7.828 -0.039   -0.025   0.1  50.825
## 513          w12 ~~          w21   7.698  0.055    0.034   0.1  25.137
## 387           w5 ~~          w14   7.678  0.042    0.029   0.1  44.096
## 425           w7 ~~          w13   7.589 -0.049   -0.034   0.1  31.535
## 444           w8 ~~          w14   7.520 -0.050   -0.034   0.1  30.457
## 113        persp =~          w15   7.504  0.114    0.080   0.1   5.794
## 481          w10 ~~          w18   7.427 -0.065   -0.035   0.1  17.426
## 334           w3 ~~           w4   7.362 -0.050   -0.036   0.1  29.477
## 270       pclass =~          w10   7.254  0.248    0.087   0.1   1.176
## 292           w1 ~~           w9   6.839  0.046    0.032   0.1  32.619
## 544          w15 ~~          w16   6.793 -0.051   -0.035   0.1  25.920
## 114        persp =~          w16   6.467 -0.120   -0.084   0.1   4.487
## 346           w3 ~~          w16   6.423 -0.041   -0.029   0.1  39.131
## 475          w10 ~~          w12   6.346 -0.075   -0.044   0.1  11.203
## 119        persp =~          w21   6.328  0.068    0.044   0.1  13.740
## 177     comprres =~           w9   6.313 -0.134   -0.101   0.1   3.516
## 342           w3 ~~          w12   6.240 -0.042   -0.029   0.1  36.013
## 311           w2 ~~           w4   6.226  0.048    0.033   0.1  27.457
## 535          w14 ~~          w18   6.219 -0.038   -0.023   0.1  42.403
## 299           w1 ~~          w16   6.208 -0.044   -0.033   0.1  31.381
## 361           w4 ~~           w9   6.198  0.046    0.030   0.1  29.716
## 622       change ~~       pclass   6.127 -0.037   -0.082   0.1  44.743
## 267       pclass =~           w7   5.945 -0.178   -0.074   0.1   1.880
## 222          wow =~          w11   5.942  0.290    0.178   0.1   0.704
## 310           w2 ~~           w3   5.677 -0.043   -0.030   0.1  30.820
## 106        persp =~           w8   5.640  0.100    0.070   0.1   5.676
## 431           w7 ~~          w19   5.597 -0.041   -0.027   0.1  33.248
## 480          w10 ~~          w17   5.164 -0.055   -0.036   0.1  17.223
## 336           w3 ~~           w6   5.064 -0.030   -0.022   0.1  55.556
## 428           w7 ~~          w16   5.058  0.049    0.034   0.1  20.942
## 315           w2 ~~           w8   5.036 -0.049   -0.034   0.1  20.585
## 489          w10 ~~       Income   4.972  0.117    0.046   0.1   3.605
## 462           w9 ~~          w15   4.963  0.041    0.027   0.1  29.406
## 412           w6 ~~          w19   4.882 -0.033   -0.021   0.1  44.911
## 611        persp ~~     humility   4.826  0.032    0.043   0.1  48.438
## 497          w11 ~~          w19   4.809  0.034    0.023   0.1  40.923
## 297           w1 ~~          w14   4.599 -0.030   -0.022   0.1  50.665
## 347           w3 ~~          w17   4.493 -0.030   -0.023   0.1  51.175
## 366           w4 ~~          w14   4.375  0.031    0.021   0.1  46.152
## 617       change ~~     humility   4.156 -0.031   -0.039   0.1  42.440
## 302           w1 ~~          w19   4.119  0.029    0.020   0.1  49.878
## 507          w12 ~~          w15   4.110 -0.040   -0.027   0.1  26.229
## 190     outsider =~           w1   4.013  0.039    0.037   0.1  25.758
## 515          w12 ~~    Uninsured   3.981 -0.186   -0.035   0.1   1.150
## 496          w11 ~~          w18   3.917  0.034    0.022   0.1  34.141
## 325           w2 ~~          w18   3.829  0.037    0.023   0.1  28.594
## 575          w18 ~~          w20   3.818  0.040    0.022   0.1  23.930
## 392           w5 ~~          w19   3.760  0.029    0.019   0.1  43.546
## 549          w15 ~~          w21   3.745  0.031    0.019   0.1  39.189
## 395           w5 ~~ WorriedMoney   3.737 -0.055   -0.030   0.1  12.361
```

```
## 467         w9 ~~         w20   3.718 -0.032   -0.019   0.1  35.726
## 263     pclass =~          w3   3.717  0.103    0.044   0.1   3.493
## 386         w5 ~~         w13   3.715  0.030    0.021   0.1  41.014
## 438         w7 ~~      Income   3.568 -0.079   -0.036   0.1   5.750
## 275     pclass =~         w15   3.450  0.117    0.047   0.1   2.539
## 201   outsider =~         w12   3.217  0.047    0.040   0.1  14.623
## 377         w4 ~~         Edu   3.201 -0.042   -0.029   0.1  17.777
## 182   comprres =~         w19   3.187  0.041    0.030   0.1  18.949
## 409         w6 ~~         w16   3.094  0.033    0.023   0.1  28.233
## 390         w5 ~~         w17   3.090  0.029    0.023   0.1  35.843
## 482        w10 ~~         w19   3.029  0.038    0.021   0.1  20.894
## 503        w11 ~~         Edu   3.011  0.043    0.031   0.1  15.996
## 264     pclass =~          w4   3.008 -0.108   -0.045   0.1   2.562
## 143     change =~   Uninsured   3.003  0.187    0.039   0.1   0.863
## 473         w9 ~~      Income   2.993 -0.073   -0.031   0.1   5.641
## 491        w11 ~~         w13   2.951 -0.028   -0.020   0.1  38.189
## 110      persp =~         w12   2.943 -0.090   -0.062   0.1   3.627
## 493        w11 ~~         w15   2.906  0.028    0.020   0.1  36.690
## 443         w8 ~~         w13   2.903 -0.032   -0.022   0.1  28.240
## 322         w2 ~~         w15   2.853  0.030    0.021   0.1  31.171
## 309         w1 ~~      Income   2.794 -0.057   -0.027   0.1   8.617
## 388         w5 ~~         w15   2.782  0.027    0.018   0.1  39.362
## 463         w9 ~~         w16   2.691 -0.036   -0.024   0.1  20.529
## 437         w7 ~~         Edu   2.682 -0.045   -0.031   0.1  13.003
## 449         w8 ~~         w19   2.605 -0.030   -0.019   0.1  29.742
## 207   outsider =~ WorriedMoney   2.556  0.052    0.036   0.1   9.389
## 192   outsider =~          w3   2.556 -0.029   -0.026   0.1  30.744
## 158   humility =~         w16   2.543 -0.082   -0.059   0.1   3.784
## 567        w17 ~~         w20   2.542 -0.024   -0.016   0.1  44.702
## 472         w9 ~~         Edu   2.538  0.045    0.029   0.1  12.782
## 326         w2 ~~         w19   2.485 -0.027   -0.017   0.1  34.287
## 404         w6 ~~         w11   2.468 -0.026   -0.019   0.1  36.535
## 445         w8 ~~         w15   2.463  0.030    0.021   0.1  27.078
## 108      persp =~         w10   2.459 -0.094   -0.057   0.1   2.755
## 254      class =~         w17   2.429  0.103    0.033   0.1   2.293
## 370         w4 ~~         w18   2.398 -0.025   -0.016   0.1  37.918
## 487        w10 ~~     OccPres   2.391  0.073    0.027   0.1   4.426
## 583        w19 ~~         w21   2.345 -0.029   -0.017   0.1  27.450
## 403         w6 ~~         w10   2.323 -0.035   -0.021   0.1  18.867
## 620     change ~~         wow   2.308 -0.317   -0.486   0.1   0.229
## 514        w12 ~~ WorriedMoney   2.307 -0.053   -0.028   0.1   8.220
## 343         w3 ~~         w13   2.296  0.020    0.014   0.1  58.136
## 422         w7 ~~         w10   2.279 -0.040   -0.024   0.1  14.014
## 382         w5 ~~          w9   2.278 -0.028   -0.019   0.1  28.423
## 356         w3 ~~      Income   2.264  0.046    0.021   0.1  10.746
## 517        w12 ~~         Edu   2.258 -0.045   -0.030   0.1  11.326
## 371         w4 ~~         w19   2.232  0.022    0.014   0.1  45.461
## 435         w7 ~~   Uninsured   2.230  0.130    0.025   0.1   1.319
## 247      class =~         w10   2.228 -0.138   -0.035   0.1   1.171
## 605  Uninsured ~~         Edu   2.207 -0.178   -0.033   0.1   0.697
## 558        w16 ~~         w20   2.197 -0.025   -0.016   0.1  34.122
## 546        w15 ~~         w18   2.184 -0.024   -0.015   0.1  37.661
## 434         w7 ~~ WorriedMoney   2.154 -0.048   -0.026   0.1   9.433
## 447         w8 ~~         w17   2.123  0.029    0.022   0.1  24.547
```

```
## 423          w7 ~~         w11   2.112  0.028    0.020   0.1  27.366
## 564         w16 ~~      Income   2.094  0.062    0.028   0.1   5.396
## 391          w5 ~~         w18   2.069 -0.024   -0.015   0.1  36.322
## 335          w3 ~~          w5   2.055 -0.020   -0.014   0.1  53.959
## 109       persp =~         w11   2.046  0.072    0.053   0.1   3.968
## 548         w15 ~~         w20   2.034 -0.020   -0.013   0.1  49.344
## 569         w17 ~~ WorriedMoney   1.991  0.041    0.024   0.1  11.603
## 531         w13 ~~      Income   1.941 -0.049   -0.022   0.1   8.126
## 556         w16 ~~         w18   1.904  0.027    0.017   0.1  26.029
## 350          w3 ~~         w20   1.896 -0.017   -0.011   0.1  68.164
## 353          w3 ~~   Uninsured   1.888 -0.087   -0.017   0.1   2.478
## 427          w7 ~~         w15   1.885 -0.025   -0.017   0.1  30.241
## 348          w3 ~~         w18   1.881  0.019    0.012   0.1  52.063
## 606   Uninsured ~~      Income   1.877  0.261    0.032   0.1   0.275
## 506         w12 ~~         w14   1.863  0.025    0.017   0.1  29.413
## 529         w13 ~~     OccPres   1.855  0.043    0.019   0.1   9.956
## 354          w3 ~~     OccPres   1.850 -0.037   -0.017   0.1  13.163
## 252       class =~         w15   1.848 -0.086   -0.025   0.1   2.524
## 368          w4 ~~         w16   1.831  0.025    0.018   0.1  28.599
## 288          w1 ~~          w5   1.779 -0.020   -0.015   0.1  43.603
## 294          w1 ~~         w11   1.767  0.021    0.016   0.1  40.735
## 585         w19 ~~   Uninsured   1.747 -0.094   -0.017   0.1   1.965
## 591         w20 ~~   Uninsured   1.740  0.090    0.016   0.1   2.148
## 532         w14 ~~         w15   1.739  0.024    0.016   0.1  31.096
## 308          w1 ~~         Edu   1.707  0.030    0.021   0.1  19.504
## 486         w10 ~~   Uninsured   1.695  0.143    0.023   0.1   0.829
## 586         w19 ~~     OccPres   1.665 -0.040   -0.016   0.1  10.498
## 112       persp =~         w14   1.660 -0.053   -0.036   0.1   5.996
## 566         w17 ~~         w19   1.640  0.020    0.014   0.1  40.889
## 539         w14 ~~ WorriedMoney   1.636  0.034    0.018   0.1  14.436
## 393          w5 ~~         w20   1.612 -0.018   -0.012   0.1  47.597
## 399          w5 ~~      Income   1.605  0.046    0.021   0.1   7.522
## 148    humility =~          w2   1.601  0.059    0.042   0.1   4.621
## 351          w3 ~~         w21   1.601 -0.017   -0.011   0.1  54.175
## 525         w13 ~~         w20   1.579 -0.018   -0.011   0.1  51.467
## 470          w9 ~~   Uninsured   1.567  0.110    0.020   0.1   1.298
## 508         w12 ~~         w16   1.550 -0.029   -0.020   0.1  18.222
## 432          w7 ~~         w20   1.539  0.021    0.013   0.1  36.351
## 339          w3 ~~          w9   1.534  0.020    0.013   0.1  40.109
## 289          w1 ~~          w6   1.527 -0.018   -0.014   0.1  44.941
## 492         w11 ~~         w14   1.479 -0.019   -0.013   0.1  40.918
## 538         w14 ~~         w21   1.475 -0.018   -0.011   0.1  44.123
## 607     OccPres ~~         Edu   1.457 -0.099   -0.042   0.1   1.495
## 553         w15 ~~         Edu   1.450  0.029    0.019   0.1  17.650
## 469          w9 ~~ WorriedMoney   1.440 -0.039   -0.020   0.1   9.284
## 144      change =~     OccPres   1.388  0.073    0.035   0.1   2.571
## 250       class =~         w13   1.378 -0.072   -0.021   0.1   2.631
## 407          w6 ~~         w14   1.342 -0.017   -0.012   0.1  45.416
## 145      change =~         Edu   1.341 -0.043   -0.032   0.1   7.371
## 627    humility ~~      pclass   1.324  0.016    0.038   0.1  49.646
## 547         w15 ~~         w19   1.314  0.017    0.011   0.1  45.149
## 273      pclass =~         w13   1.302 -0.070   -0.029   0.1   2.647
## 185    comprres =~ WorriedMoney   1.287  0.042    0.026   0.1   7.203
## 440          w8 ~~         w10   1.276 -0.032   -0.019   0.1  12.542
```

```
## 523          w13 ~~          w18   1.251 -0.018    -0.011   0.1  39.286
## 132       change =~          w12   1.245  0.048     0.036   0.1   5.420
## 518          w12 ~~       Income   1.219  0.049     0.022   0.1   5.005
## 323           w2 ~~          w16   1.218  0.024     0.016   0.1  21.594
## 167     humility =~          Edu   1.215  0.044     0.032   0.1   6.136
## 238        class =~           w1   1.200 -0.066    -0.021   0.1   2.794
## 537          w14 ~~          w20   1.183  0.015     0.009   0.1  55.531
## 466           w9 ~~          w19   1.182  0.019     0.012   0.1  32.694
## 476          w10 ~~          w13   1.168 -0.024    -0.014   0.1  19.764
## 516          w12 ~~      OccPres   1.126 -0.043    -0.018   0.1   6.133
## 396           w5 ~~    Uninsured   1.111  0.080     0.015   0.1   1.729
## 349           w3 ~~          w19   1.111  0.013     0.009   0.1  62.398
## 446           w8 ~~          w16   1.105  0.024     0.017   0.1  18.739
## 116        persp =~          w18   1.102  0.029     0.018   0.1  13.207
## 595          w21 ~~ WorriedMoney   1.097  0.030     0.015   0.1  12.188
## 571          w17 ~~      OccPres   1.079 -0.035    -0.017   0.1   8.661
## 494          w11 ~~          w16   1.062  0.020     0.015   0.1  25.674
## 421           w7 ~~           w9   1.051 -0.022    -0.015   0.1  21.881
## 608      OccPres ~~       Income   1.037  0.137     0.039   0.1   0.550
## 372           w4 ~~          w20   1.036 -0.014    -0.009   0.1  49.691
## 179     comprres =~          w11   1.030  0.051     0.041   0.1   4.031
## 464           w9 ~~          w17   1.020 -0.019    -0.014   0.1  26.860
## 413           w6 ~~          w20   1.008  0.014     0.009   0.1  49.086
## 268       pclass =~           w8   1.000 -0.077    -0.032   0.1   1.682
## 115        persp =~          w17   0.970 -0.041    -0.032   0.1   5.792
## 592          w20 ~~      OccPres   0.956  0.029     0.011   0.1  11.478
## 460           w9 ~~          w13   0.954 -0.018    -0.012   0.1  30.620
## 550          w15 ~~ WorriedMoney   0.953 -0.027    -0.015   0.1  12.818
## 542          w14 ~~          Edu   0.920 -0.022    -0.014   0.1  19.865
## 545          w15 ~~          w17   0.919 -0.017    -0.013   0.1  33.216
## 598          w21 ~~          Edu   0.916 -0.023    -0.015   0.1  16.804
## 479          w10 ~~          w16   0.916 -0.026    -0.016   0.1  13.152
## 146       change =~       Income   0.907 -0.064    -0.032   0.1   2.208
## 227          wow =~          w16   0.882 -0.097    -0.056   0.1   0.943
## 355           w3 ~~          Edu   0.865  0.019     0.013   0.1  24.386
## 307           w1 ~~      OccPres   0.858  0.029     0.013   0.1  10.559
## 291           w1 ~~           w8   0.835 -0.017    -0.012   0.1  29.929
## 527          w13 ~~ WorriedMoney   0.835  0.025     0.013   0.1  13.372
## 337           w3 ~~           w7   0.819 -0.014    -0.010   0.1  41.697
## 554          w15 ~~       Income   0.808  0.032     0.014   0.1   7.792
## 509          w12 ~~          w17   0.805 -0.018    -0.014   0.1  23.857
## 220          wow =~           w9   0.777  0.109     0.061   0.1   0.654
## 619       change ~~    outsider   0.760  0.016     0.016   0.1  29.443
## 398           w5 ~~          Edu   0.720 -0.021    -0.014   0.1  17.028
## 478          w10 ~~          w15   0.693  0.019     0.011   0.1  18.959
## 577          w18 ~~ WorriedMoney   0.692 -0.024    -0.012   0.1  11.713
## 375           w4 ~~    Uninsured   0.686  0.062     0.012   0.1   1.805
## 510          w12 ~~          w18   0.678 -0.017    -0.010   0.1  24.158
## 495          w11 ~~          w17   0.668  0.014     0.011   0.1  33.580
## 572          w17 ~~          Edu   0.665  0.020     0.015   0.1  15.990
## 389           w5 ~~          w16   0.663  0.016     0.011   0.1  27.382
## 180     comprres =~          w12   0.662  0.041     0.032   0.1   3.872
## 269       pclass =~           w9   0.649 -0.059    -0.023   0.1   1.836
## 313           w2 ~~           w6   0.648  0.014     0.010   0.1  31.007
```

```
## 187     comprres =~      OccPres   0.636  0.053    0.026   0.1   2.248
## 629     comprres ~~          wow   0.636  0.150    0.224   0.1   0.282
## 276       pclass =~          w16   0.634  0.060    0.024   0.1   1.761
## 633     outsider ~~        class   0.632  0.008    0.020   0.1 110.805
## 300           w1 ~~          w17   0.631 -0.012   -0.010   0.1  41.086
## 246        class =~           w9   0.626  0.059    0.017   0.1   1.826
## 419           w6 ~~       Income   0.615 -0.028   -0.013   0.1   7.756
## 632     outsider ~~          wow   0.601 -0.200   -0.266   0.1   0.150
## 397           w5 ~~      OccPres   0.597  0.025    0.011   0.1   9.216
## 384           w5 ~~          w11   0.593 -0.013   -0.009   0.1  35.477
## 452           w8 ~~ WorriedMoney   0.586  0.026    0.014   0.1   8.438
## 235          wow =~      OccPres   0.580  0.077    0.028   0.1   0.972
## 394           w5 ~~          w21   0.572 -0.012   -0.008   0.1  37.795
## 256        class =~          w19   0.555  0.045    0.012   0.1   2.789
## 293           w1 ~~          w10   0.549  0.016    0.010   0.1  20.977
## 483          w10 ~~          w20   0.549  0.016    0.008   0.1  22.842
## 562          w16 ~~      OccPres   0.548 -0.029   -0.012   0.1   6.613
## 327           w2 ~~          w20   0.542 -0.012   -0.007   0.1  37.485
## 415           w6 ~~ WorriedMoney   0.538  0.021    0.011   0.1  12.749
## 266       pclass =~           w6   0.527 -0.046   -0.019   0.1   2.529
## 120        persp =~ WorriedMoney   0.507  0.030    0.016   0.1   5.773
## 500          w11 ~~ WorriedMoney   0.500  0.021    0.012   0.1  11.624
## 526          w13 ~~          w21   0.490 -0.011   -0.007   0.1  40.880
## 504          w11 ~~       Income   0.484 -0.026   -0.012   0.1   7.054
## 530          w13 ~~          Edu   0.438 -0.015   -0.010   0.1  18.411
## 340           w3 ~~          w10   0.429  0.013    0.008   0.1  26.081
## 330           w2 ~~    Uninsured   0.427  0.056    0.011   0.1   1.361
## 304           w1 ~~          w21   0.425 -0.010   -0.007   0.1  43.290
## 328           w2 ~~          w21   0.421 -0.012   -0.007   0.1  29.754
## 204     outsider =~          w15   0.420  0.014    0.012   0.1  22.835
## 138       change =~          w18   0.420  0.017    0.011   0.1  15.411
## 477          w10 ~~          w14   0.414 -0.014   -0.008   0.1  21.285
## 224          wow =~          w13   0.413  0.060    0.035   0.1   1.159
## 123        persp =~          Edu   0.412  0.025    0.018   0.1   6.339
## 248        class =~          w11   0.408  0.042    0.013   0.1   2.281
## 165     humility =~    Uninsured   0.405  0.074    0.015   0.1   0.745
## 484          w10 ~~          w21   0.400  0.015    0.008   0.1  18.133
## 376           w4 ~~      OccPres   0.397  0.020    0.009   0.1   9.623
## 367           w4 ~~          w15   0.393  0.010    0.007   0.1  41.160
## 147     humility =~           w1   0.393  0.025    0.020   0.1   6.175
## 551          w15 ~~    Uninsured   0.385 -0.046   -0.009   0.1   1.792
## 233          wow =~ WorriedMoney   0.383  0.032    0.014   0.1   3.833
## 573          w17 ~~       Income   0.381  0.023    0.011   0.1   7.068
## 374           w4 ~~ WorriedMoney   0.381  0.017    0.009   0.1  12.903
## 416           w6 ~~    Uninsured   0.379  0.046    0.009   0.1   1.783
## 630     comprres ~~        class   0.367 -0.004   -0.012   0.1 208.854
## 579          w18 ~~      OccPres   0.359 -0.020   -0.008   0.1   8.755
## 319           w2 ~~          w12   0.357  0.013    0.009   0.1  20.014
## 281       pclass =~          w21   0.357 -0.038   -0.014   0.1   2.443
## 242        class =~           w5   0.353 -0.038   -0.011   0.1   2.438
## 253        class =~          w16   0.352  0.045    0.013   0.1   1.751
## 587          w19 ~~          Edu   0.347  0.013    0.008   0.1  19.365
## 205     outsider =~          w16   0.335 -0.014   -0.013   0.1  16.189
## 317           w2 ~~          w10   0.333 -0.015   -0.009   0.1  14.447
```

```
## 405          w6 ~~          w12   0.332  0.011    0.008   0.1  26.105
## 274      pclass =~          w14   0.330 -0.034   -0.014   0.1   2.850
## 314          w2 ~~           w7   0.328 -0.012   -0.008   0.1  23.005
## 560         w16 ~~ WorriedMoney   0.322 -0.019   -0.010   0.1   8.855
## 616       persp ~~       pclass   0.319  0.007    0.017   0.1  66.342
## 239       class =~           w2   0.316  0.041    0.012   0.1   1.924
## 528         w13 ~~    Uninsured   0.313 -0.041   -0.008   0.1   1.870
## 559         w16 ~~          w21   0.282  0.010    0.006   0.1  27.084
## 359          w4 ~~           w7   0.279 -0.010   -0.007   0.1  30.471
## 540         w14 ~~    Uninsured   0.278 -0.037   -0.007   0.1   2.018
## 363          w4 ~~          w11   0.273 -0.009   -0.006   0.1  37.104
## 584         w19 ~~ WorriedMoney   0.271  0.014    0.007   0.1  14.046
## 278      pclass =~          w18   0.267 -0.034   -0.012   0.1   2.347
## 234         wow =~    Uninsured   0.264  0.073    0.012   0.1   0.501
## 511         w12 ~~          w19   0.263 -0.010   -0.006   0.1  28.964
## 296          w1 ~~          w13   0.263  0.007    0.005   0.1  47.082
## 244       class =~           w7   0.252  0.037    0.011   0.1   1.867
## 455          w8 ~~          Edu   0.248 -0.015   -0.010   0.1  11.632
## 604   Uninsured ~~      OccPres   0.243 -0.854   -0.102   0.1   0.003
## 324          w2 ~~          w17   0.241 -0.009   -0.007   0.1  28.282
## 249       class =~          w12   0.236 -0.038   -0.011   0.1   1.622
## 265      pclass =~           w5   0.231  0.031    0.013   0.1   2.454
## 303          w1 ~~          w20   0.231  0.007    0.004   0.1  54.521
## 552         w15 ~~      OccPres   0.225  0.015    0.007   0.1   9.548
## 243       class =~           w6   0.219  0.029    0.009   0.1   2.514
## 364          w4 ~~          w12   0.218 -0.009   -0.006   0.1  26.460
## 597         w21 ~~      OccPres   0.217  0.015    0.006   0.1   9.110
## 257       class =~          w20   0.211 -0.026   -0.007   0.1   3.049
## 625    humility ~~          wow   0.210  0.091    0.147   0.1   0.255
## 588         w19 ~~       Income   0.200  0.015    0.006   0.1   8.566
## 441          w8 ~~          w11   0.198  0.009    0.006   0.1  24.520
## 241       class =~           w4   0.198  0.028    0.008   0.1   2.546
## 590         w20 ~~ WorriedMoney   0.197  0.011    0.006   0.1  15.359
## 456          w8 ~~       Income   0.195 -0.019   -0.009   0.1   5.144
## 631    comprres ~~       pclass   0.193  0.006    0.013   0.1  55.074
## 155    humility =~          w13   0.184 -0.019   -0.014   0.1   5.013
## 142      change =~ WorriedMoney   0.181 -0.017   -0.010   0.1   6.469
## 378          w4 ~~       Income   0.175 -0.015   -0.007   0.1   7.854
## 502         w11 ~~      OccPres   0.175 -0.014   -0.006   0.1   8.642
## 338          w3 ~~           w8   0.165  0.007    0.005   0.1  37.370
## 442          w8 ~~          w12   0.165  0.010    0.007   0.1  17.381
## 229         wow =~          w18   0.161  0.014    0.008   0.1   7.686
## 448          w8 ~~          w18   0.155  0.008    0.005   0.1  24.803
## 255       class =~          w18   0.151  0.025    0.007   0.1   2.326
## 406          w6 ~~          w13   0.150  0.006    0.004   0.1  42.257
## 424          w7 ~~          w12   0.150  0.009    0.006   0.1  19.417
## 557         w16 ~~          w19   0.146 -0.007   -0.004   0.1  31.210
## 344          w3 ~~          w14   0.145 -0.005   -0.003   0.1  62.259
## 122       persp =~      OccPres   0.141  0.027    0.012   0.1   1.944
## 563         w16 ~~          Edu   0.135 -0.010   -0.007   0.1  12.205
## 191    outsider =~           w2   0.134 -0.009   -0.008   0.1  18.034
## 277      pclass =~          w17   0.128  0.024    0.011   0.1   2.306
## 345          w3 ~~          w15   0.127 -0.005   -0.003   0.1  55.863
## 358          w4 ~~           w6   0.126 -0.006   -0.004   0.1  40.947
```

```
## 166     humility =~      OccPres   0.125  0.026    0.012   0.1   1.815
## 485          w10 ~~ WorriedMoney   0.122 -0.014   -0.007   0.1   5.929
## 332           w2 ~~          Edu   0.121  0.010    0.006   0.1  13.408
## 614        persp ~~          wow   0.119  0.059    0.099   0.1   0.340
## 282       pclass =~ WorriedMoney   0.116 -0.043   -0.014   0.1   0.624
## 561          w16 ~~    Uninsured   0.116  0.031    0.006   0.1   1.239
## 280       pclass =~          w20   0.115  0.019    0.007   0.1   3.076
## 306           w1 ~~    Uninsured   0.114 -0.024   -0.005   0.1   1.980
## 570          w17 ~~    Uninsured   0.114  0.027    0.006   0.1   1.623
## 599          w21 ~~       Income   0.114  0.012    0.005   0.1   7.434
## 168     humility =~       Income   0.112 -0.026   -0.013   0.1   1.598
## 594          w20 ~~       Income   0.109 -0.011   -0.004   0.1   9.366
## 236          wow =~          Edu   0.109  0.017    0.010   0.1   3.982
## 237          wow =~       Income   0.109 -0.035   -0.013   0.1   0.884
## 259        class =~          Edu   0.109 -0.177   -0.052   0.1   0.035
## 245        class =~           w8   0.109  0.026    0.008   0.1   1.671
## 260        class =~       Income   0.109  0.375    0.073   0.1   0.008
## 129       change =~           w9   0.108  0.014    0.010   0.1   5.463
## 295           w1 ~~          w12   0.102  0.006    0.004   0.1  29.038
## 626     humility ~~        class   0.101  0.002    0.007   0.1 188.314
## 223          wow =~          w12   0.099  0.035    0.020   0.1   0.824
## 488          w10 ~~          Edu   0.096  0.011    0.006   0.1   8.155
## 613        persp ~~     outsider   0.095 -0.005   -0.005   0.1  41.714
## 194     outsider =~           w5   0.092  0.006    0.006   0.1  22.454
## 210     outsider =~          Edu   0.084 -0.008   -0.007   0.1  12.011
## 139       change =~          w19   0.082  0.007    0.005   0.1  18.188
## 279       pclass =~          w19   0.076  0.016    0.006   0.1   2.814
## 251        class =~          w14   0.074  0.016    0.005   0.1   2.833
## 498          w11 ~~          w20   0.074 -0.004   -0.003   0.1  44.711
## 603 WorriedMoney ~~       Income   0.073 -0.018   -0.006   0.1   2.345
## 159     humility =~          w17   0.072  0.012    0.010   0.1   4.887
## 581          w18 ~~       Income   0.070 -0.010   -0.004   0.1   7.144
## 471           w9 ~~      OccPres   0.068 -0.010   -0.004   0.1   6.911
## 181     comprres =~          w18   0.061 -0.006   -0.004   0.1  16.137
## 568          w17 ~~          w21   0.060 -0.004   -0.003   0.1  35.485
## 453           w8 ~~    Uninsured   0.059 -0.022   -0.004   0.1   1.180
## 600 WorriedMoney ~~    Uninsured   0.059 -0.054   -0.008   0.1   0.202
## 450           w8 ~~          w20   0.053  0.004    0.003   0.1  32.519
## 341           w3 ~~          w11   0.051 -0.003   -0.002   0.1  49.889
## 133       change =~          w13   0.051 -0.008   -0.006   0.1   8.795
## 580          w18 ~~          Edu   0.050 -0.006   -0.003   0.1  16.149
## 160     humility =~          w18   0.048  0.006    0.004   0.1  12.586
## 124        persp =~       Income   0.048 -0.017   -0.008   0.1   1.702
## 436           w7 ~~      OccPres   0.044  0.008    0.003   0.1   7.046
## 418           w6 ~~          Edu   0.043 -0.005   -0.003   0.1  17.560
## 362           w4 ~~          w10   0.041 -0.005   -0.003   0.1  19.116
## 283       pclass =~    Uninsured   0.041  0.080    0.009   0.1   0.064
## 272       pclass =~          w12   0.041  0.016    0.006   0.1   1.632
## 541          w14 ~~      OccPres   0.040 -0.006   -0.003   0.1  10.731
## 188     comprres =~          Edu   0.038  0.007    0.005   0.1   7.787
## 383           w5 ~~          w10   0.037 -0.004   -0.003   0.1  18.302
## 131       change =~          w11   0.034 -0.007   -0.006   0.1   6.548
## 301           w1 ~~          w18   0.034  0.003    0.002   0.1  41.602
## 333           w2 ~~       Income   0.032  0.007    0.003   0.1   5.928
```

```
## 360          w4 ~~           w8   0.030 -0.003   -0.002   0.1  27.277
## 578         w18 ~~    Uninsured   0.030 -0.014   -0.002   0.1   1.638
## 271      pclass =~          w11   0.028  0.011    0.005   0.1   2.294
## 499         w11 ~~          w21   0.027  0.003    0.002   0.1  35.526
## 512         w12 ~~          w20   0.024  0.003    0.002   0.1  31.660
## 209    outsider =~      OccPres   0.023 -0.006   -0.004   0.1   5.649
## 634    outsider ~~       pclass   0.022 -0.003   -0.005   0.1  29.211
## 385          w5 ~~          w12   0.021  0.003    0.002   0.1  25.328
## 261      pclass =~           w1   0.018 -0.008   -0.003   0.1   2.811
## 305          w1 ~~ WorriedMoney   0.017  0.004    0.002   0.1  14.157
## 121       persp =~    Uninsured   0.015  0.014    0.003   0.1   0.764
## 208    outsider =~    Uninsured   0.015  0.011    0.003   0.1   1.288
## 596         w21 ~~    Uninsured   0.012 -0.008   -0.001   0.1   1.705
## 331          w2 ~~      OccPres   0.011 -0.004   -0.002   0.1   7.265
## 352          w3 ~~ WorriedMoney   0.010 -0.002   -0.001   0.1  17.728
## 318          w2 ~~          w11   0.009  0.002    0.001   0.1  28.188
## 196    outsider =~           w7   0.009 -0.002   -0.002   0.1  18.000
## 240       class =~           w3   0.007  0.005    0.001   0.1   3.473
## 454          w8 ~~      OccPres   0.007  0.003    0.001   0.1   6.304
## 501         w11 ~~    Uninsured   0.005  0.006    0.001   0.1   1.625
## 199    outsider =~          w10   0.005  0.002    0.002   0.1  10.651
## 411          w6 ~~          w18   0.005 -0.001   -0.001   0.1  37.461
## 593         w20 ~~          Edu   0.005  0.002    0.001   0.1  21.175
## 258       class =~          w21   0.005  0.004    0.001   0.1   2.421
## 211    outsider =~       Income   0.005  0.003    0.002   0.1   4.676
## 189    comprres =~       Income   0.004  0.005    0.002   0.1   1.987
## 373          w4 ~~          w21   0.004  0.001    0.001   0.1  39.456
## 621      change ~~        class   0.004  0.000   -0.001   0.1 169.707
## 602 WorriedMoney ~~          Edu   0.004 -0.003   -0.001   0.1   5.396
## 417          w6 ~~      OccPres   0.003  0.002    0.001   0.1   9.503
## 465          w9 ~~          w18   0.003 -0.001   -0.001   0.1  27.273
## 543         w14 ~~       Income   0.002 -0.001   -0.001   0.1   8.760
## 369          w4 ~~          w17   0.001  0.001    0.000   0.1  37.442
## 601 WorriedMoney ~~      OccPres   0.001  0.006    0.002   0.1   0.308
## 329          w2 ~~ WorriedMoney   0.001 -0.001    0.000   0.1   9.728
## 186    comprres =~    Uninsured   0.000  0.002    0.000   0.1   0.950
## 433          w7 ~~          w21   0.000  0.000    0.000   0.1  28.853
## 615       persp ~~        class   0.000  0.000    0.000   0.1 251.599
## 298          w1 ~~          w15   0.000  0.000    0.000   0.1  45.175
## 193    outsider =~           w4   0.000  0.000    0.000   0.1  23.435
## 524         w13 ~~          w19   0.000  0.000    0.000   0.1  47.096
## 262      pclass =~           w2   0.000 -0.001    0.000   0.1   1.936
## 284      pclass =~      OccPres   0.000  0.003    0.001   0.1   0.116
## 365          w4 ~~          w13   0.000  0.000    0.000   0.1  42.895
## 408          w6 ~~          w15   0.000  0.000    0.000   0.1  40.560
## 414          w6 ~~          w21   0.000  0.000    0.000   0.1  38.980
## 164    humility =~ WorriedMoney   0.000  0.000    0.000   0.1   5.644
##     power decision
## 555 0.999  *epc:m*
## 420 0.990  *epc:m*
## 380 0.995   epc:nm
## 519 1.000  *epc:m*
## 401 0.995   epc:nm
## 286 1.000  *epc:m*
```

```
## 379 0.969  *epc:m*
## 173 0.755  *epc:m*
## 457 0.926  *epc:m*
## 439 0.993  *epc:m*
## 522 1.000   epc:nm
## 216 0.303  **(m)**
## 624 1.000  *epc:m*
## 154 0.615  **(m)**
## 628 1.000   epc:nm
## 381 0.994   epc:nm
## 105 0.697  **(m)**
## 170 0.685  **(m)**
## 218 0.329  **(m)**
## 137 0.820  *epc:m*
## 152 0.661  **(m)**
## 618 1.000  *epc:m*
## 217 0.300  **(m)**
## 400 0.995  *epc:m*
## 203 0.999   epc:nm
## 103 0.712  **(m)**
## 231 0.867   epc:nm
## 214 0.242  **(m)**
## 183 0.994   epc:nm
## 175 0.755   epc:nm
## 521 0.999   epc:nm
## 104 0.714  **(m)**
## 118 0.982   epc:nm
## 534 1.000   epc:nm
## 215 0.249  **(m)**
## 197 0.981  *epc:m*
## 163 0.951  *epc:m*
## 162 0.977   epc:nm
## 610 1.000   epc:nm
## 225 0.186  **(m)**
## 127 0.912   epc:nm
## 125 0.891   epc:nm
## 219 0.313  **(m)**
## 213 0.223  **(m)**
## 576 0.999   epc:nm
## 172 0.760  *epc:m*
## 533 1.000   epc:nm
## 451 0.999  *epc:m*
## 623 1.000   epc:nm
## 156 0.616  **(m)**
## 490 0.988  *epc:m*
## 287 1.000   epc:nm
## 200 0.994  *epc:m*
## 285 1.000   epc:nm
## 153 0.648  **(m)**
## 128 0.860  *epc:m*
## 171 0.786   epc:nm
## 458 0.969   epc:nm
## 232 0.807  *epc:m*
## 589 0.999   epc:nm
```

```
## 141 0.980  *epc:m*
## 151 0.660  **(m)**
## 130 0.520  **(m)**
## 221 0.127  **(m)**
## 206 0.996   epc:nm
## 468 1.000   epc:nm
## 565 1.000   epc:nm
## 150 0.656  **(m)**
## 140 0.993   epc:nm
## 178 0.406  **(m)**
## 226 0.188  **(m)**
## 357 1.000   epc:nm
## 316 0.997   epc:nm
## 459 0.973   epc:nm
## 169 0.801   epc:nm
## 290 1.000   epc:nm
## 126 0.778  *epc:m*
## 612 1.000   epc:nm
## 157 0.600  **(m)**
## 149 0.700  **(m)**
## 184 0.984   epc:nm
## 134 0.855   epc:nm
## 161 0.970   epc:nm
## 429 0.999   epc:nm
## 202 0.998   epc:nm
## 117 0.976   epc:nm
## 461 1.000   epc:nm
## 198 0.979   epc:nm
## 321 1.000   epc:nm
## 136 0.714  **(m)**
## 174 0.755   epc:nm
## 212 0.271  **(m)**
## 402 1.000   epc:nm
## 111 0.682  **(m)**
## 574 0.999   epc:nm
## 520 1.000   epc:nm
## 176 0.723  **(m)**
## 582 0.997   epc:nm
## 195 0.998   epc:nm
## 426 1.000   epc:nm
## 410 1.000   epc:nm
## 320 1.000   epc:nm
## 312 1.000   epc:nm
## 107 0.455  **(m)**
## 505 0.999   epc:nm
## 228 0.196  **(m)**
## 230 0.851   epc:nm
## 430 1.000   epc:nm
## 474 0.960   epc:nm
## 135 0.832   epc:nm
## 536 1.000   epc:nm
## 513 0.999   epc:nm
## 387 1.000   epc:nm
## 425 1.000   epc:nm
```

```
## 444 1.000   epc:nm
## 113 0.673  **(m)**
## 481 0.987   epc:nm
## 334 1.000   epc:nm
## 270 0.192  **(m)**
## 292 1.000   epc:nm
## 544 0.999   epc:nm
## 114 0.563  **(m)**
## 346 1.000   epc:nm
## 475 0.917   epc:nm
## 119 0.960   epc:nm
## 177 0.466  **(m)**
## 342 1.000   epc:nm
## 311 0.999   epc:nm
## 535 1.000   epc:nm
## 299 1.000   epc:nm
## 361 1.000   epc:nm
## 622 1.000   epc:nm
## 267 0.278  **(m)**
## 222 0.134  **(m)**
## 310 1.000   epc:nm
## 106 0.664  **(m)**
## 431 1.000   epc:nm
## 480 0.986   epc:nm
## 336 1.000   epc:nm
## 428 0.996   epc:nm
## 315 0.995   epc:nm
## 489 0.476  **(m)**
## 462 1.000   epc:nm
## 412 1.000   epc:nm
## 611 1.000   epc:nm
## 497 1.000   epc:nm
## 297 1.000   epc:nm
## 347 1.000   epc:nm
## 366 1.000   epc:nm
## 617 1.000   epc:nm
## 302 1.000   epc:nm
## 507 0.999   epc:nm
## 190 0.999   epc:nm
## 515 0.189  **(m)**
## 496 1.000   epc:nm
## 325 1.000     (nm)
## 575 0.998     (nm)
## 392 1.000     (nm)
## 549 1.000     (nm)
## 395 0.940     (nm)
## 467 1.000     (nm)
## 263 0.464      (i)
## 386 1.000     (nm)
## 438 0.669      (i)
## 275 0.357      (i)
## 201 0.969     (nm)
## 377 0.988     (nm)
## 182 0.992     (nm)
```

```
## 409 1.000     (nm)
## 390 1.000     (nm)
## 482 0.995     (nm)
## 503 0.979     (nm)
## 264 0.360      (i)
## 143 0.153      (i)
## 473 0.661      (i)
## 491 1.000     (nm)
## 110 0.478      (i)
## 493 1.000     (nm)
## 443 1.000     (nm)
## 322 1.000     (nm)
## 309 0.835     (nm)
## 388 1.000     (nm)
## 463 0.995     (nm)
## 437 0.950     (nm)
## 449 1.000     (nm)
## 207 0.865     (nm)
## 192 1.000     (nm)
## 158 0.494      (i)
## 567 1.000     (nm)
## 472 0.947     (nm)
## 326 1.000     (nm)
## 404 1.000     (nm)
## 445 0.999     (nm)
## 108 0.382      (i)
## 254 0.328      (i)
## 370 1.000     (nm)
## 487 0.557      (i)
## 583 0.999     (nm)
## 403 0.991     (nm)
## 620 0.077      (i)
## 514 0.818     (nm)
## 343 1.000     (nm)
## 422 0.963     (nm)
## 382 1.000     (nm)
## 356 0.906     (nm)
## 517 0.920     (nm)
## 371 1.000     (nm)
## 435 0.210      (i)
## 247 0.191      (i)
## 605 0.133      (i)
## 558 1.000     (nm)
## 546 1.000     (nm)
## 434 0.867     (nm)
## 447 0.999     (nm)
## 423 0.999     (nm)
## 564 0.642      (i)
## 391 1.000     (nm)
## 335 1.000     (nm)
## 109 0.513      (i)
## 548 1.000     (nm)
## 569 0.926     (nm)
## 531 0.813     (nm)
```

```
## 556 0.999     (nm)
## 350 1.000     (nm)
## 353 0.350      (i)
## 427 1.000     (nm)
## 348 1.000     (nm)
## 606 0.082      (i)
## 506 1.000     (nm)
## 529 0.884     (nm)
## 354 0.952     (nm)
## 252 0.355      (i)
## 368 1.000     (nm)
## 288 1.000     (nm)
## 294 1.000     (nm)
## 585 0.289      (i)
## 591 0.311      (i)
## 532 1.000     (nm)
## 308 0.993     (nm)
## 486 0.149      (i)
## 586 0.900     (nm)
## 112 0.687      (i)
## 566 1.000     (nm)
## 539 0.967     (nm)
## 393 1.000     (nm)
## 399 0.783     (nm)
## 148 0.575      (i)
## 351 1.000     (nm)
## 525 1.000     (nm)
## 470 0.207      (i)
## 508 0.990     (nm)
## 432 1.000     (nm)
## 339 1.000     (nm)
## 289 1.000     (nm)
## 492 1.000     (nm)
## 538 1.000     (nm)
## 607 0.231      (i)
## 553 0.987     (nm)
## 469 0.861     (nm)
## 144 0.361      (i)
## 250 0.368      (i)
## 407 1.000     (nm)
## 145 0.775     (nm)
## 627 1.000     (nm)
## 547 1.000     (nm)
## 273 0.370      (i)
## 185 0.765     (nm)
## 440 0.943     (nm)
## 523 1.000     (nm)
## 132 0.644      (i)
## 518 0.609      (i)
## 323 0.996     (nm)
## 167 0.697      (i)
## 238 0.387      (i)
## 537 1.000     (nm)
## 466 1.000     (nm)
```

```
## 476 0.994     (nm)
## 516 0.697      (i)
## 396 0.260      (i)
## 349 1.000     (nm)
## 446 0.991     (nm)
## 116 0.953     (nm)
## 595 0.937     (nm)
## 571 0.837     (nm)
## 494 0.999     (nm)
## 421 0.997     (nm)
## 608 0.115      (i)
## 372 1.000     (nm)
## 179 0.519      (i)
## 464 0.999     (nm)
## 413 1.000     (nm)
## 268 0.254      (i)
## 115 0.672      (i)
## 592 0.923     (nm)
## 460 1.000     (nm)
## 550 0.947     (nm)
## 542 0.994     (nm)
## 545 1.000     (nm)
## 598 0.984     (nm)
## 479 0.952     (nm)
## 146 0.318      (i)
## 227 0.163      (i)
## 355 0.999     (nm)
## 307 0.901     (nm)
## 291 1.000     (nm)
## 527 0.955     (nm)
## 337 1.000     (nm)
## 554 0.797     (nm)
## 509 0.998     (nm)
## 220 0.128      (i)
## 619 1.000     (nm)
## 398 0.985     (nm)
## 478 0.992     (nm)
## 577 0.928     (nm)
## 375 0.269      (i)
## 510 0.998     (nm)
## 495 1.000     (nm)
## 572 0.979     (nm)
## 389 0.999     (nm)
## 180 0.503      (i)
## 269 0.273      (i)
## 313 1.000     (nm)
## 187 0.323      (i)
## 629 0.083      (i)
## 276 0.264      (i)
## 633 1.000     (nm)
## 300 1.000     (nm)
## 246 0.272      (i)
## 419 0.795     (nm)
## 632 0.067      (i)
```

```
## 397 0.859     (nm)
## 384 1.000     (nm)
## 452 0.828     (nm)
## 235 0.167      (i)
## 394 1.000     (nm)
## 256 0.386      (i)
## 293 0.996     (nm)
## 483 0.998     (nm)
## 562 0.730      (i)
## 327 1.000     (nm)
## 415 0.946     (nm)
## 266 0.356      (i)
## 120 0.671      (i)
## 500 0.926     (nm)
## 526 1.000     (nm)
## 504 0.757     (nm)
## 530 0.990     (nm)
## 340 0.999     (nm)
## 330 0.215      (i)
## 304 1.000     (nm)
## 328 1.000     (nm)
## 204 0.998     (nm)
## 138 0.975     (nm)
## 477 0.996     (nm)
## 224 0.190      (i)
## 123 0.711      (i)
## 248 0.327      (i)
## 165 0.139      (i)
## 484 0.989     (nm)
## 376 0.873     (nm)
## 367 1.000     (nm)
## 147 0.700      (i)
## 551 0.268      (i)
## 233 0.499      (i)
## 573 0.758     (nm)
## 374 0.949     (nm)
## 416 0.267      (i)
## 630 1.000     (nm)
## 579 0.841     (nm)
## 319 0.994     (nm)
## 281 0.346      (i)
## 242 0.345      (i)
## 253 0.263      (i)
## 587 0.993     (nm)
## 205 0.980     (nm)
## 317 0.967     (nm)
## 405 0.999     (nm)
## 274 0.393      (i)
## 314 0.998     (nm)
## 560 0.845     (nm)
## 616 1.000     (nm)
## 239 0.284      (i)
## 528 0.277      (i)
## 559 0.999     (nm)
```

```
## 359 1.000     (nm)
## 540 0.295      (i)
## 363 1.000     (nm)
## 584 0.963     (nm)
## 278 0.335      (i)
## 234 0.109      (i)
## 511 1.000     (nm)
## 296 1.000     (nm)
## 244 0.277      (i)
## 455 0.927     (nm)
## 604 0.050      (i)
## 324 1.000     (nm)
## 249 0.247      (i)
## 265 0.347      (i)
## 303 1.000     (nm)
## 552 0.871     (nm)
## 243 0.354      (i)
## 364 0.999     (nm)
## 597 0.855     (nm)
## 257 0.415      (i)
## 625 0.080      (i)
## 588 0.833     (nm)
## 441 0.999     (nm)
## 241 0.358      (i)
## 590 0.975     (nm)
## 456 0.621      (i)
## 631 1.000     (nm)
## 155 0.610      (i)
## 142 0.720      (i)
## 378 0.800     (nm)
## 502 0.836     (nm)
## 338 1.000     (nm)
## 442 0.986     (nm)
## 229 0.792     (nm)
## 448 0.999     (nm)
## 255 0.332      (i)
## 406 1.000     (nm)
## 424 0.993     (nm)
## 557 1.000     (nm)
## 344 1.000     (nm)
## 122 0.286      (i)
## 563 0.937     (nm)
## 191 0.989     (nm)
## 277 0.330      (i)
## 345 1.000     (nm)
## 358 1.000     (nm)
## 166 0.270      (i)
## 485 0.683      (i)
## 332 0.956     (nm)
## 614 0.090      (i)
## 282 0.124      (i)
## 561 0.200      (i)
## 280 0.418      (i)
## 306 0.291      (i)
```

```
## 570 0.247      (i)
## 599 0.778     (nm)
## 168 0.244      (i)
## 594 0.864     (nm)
## 236 0.514      (i)
## 237 0.156      (i)
## 259 0.054      (i)
## 245 0.253      (i)
## 260 0.051      (i)
## 129 0.647      (i)
## 295 1.000     (nm)
## 626 1.000     (nm)
## 223 0.148      (i)
## 488 0.815     (nm)
## 613 1.000     (nm)
## 194 0.997     (nm)
## 210 0.934     (nm)
## 139 0.989     (nm)
## 279 0.389      (i)
## 251 0.391      (i)
## 498 1.000     (nm)
## 603 0.334      (i)
## 159 0.599      (i)
## 581 0.762     (nm)
## 471 0.748      (i)
## 181 0.980     (nm)
## 568 1.000     (nm)
## 453 0.192      (i)
## 600 0.073      (i)
## 450 1.000     (nm)
## 341 1.000     (nm)
## 133 0.843     (nm)
## 580 0.980     (nm)
## 160 0.944     (nm)
## 124 0.257      (i)
## 436 0.756     (nm)
## 418 0.987     (nm)
## 362 0.992     (nm)
## 283 0.057      (i)
## 272 0.248      (i)
## 541 0.906     (nm)
## 188 0.797     (nm)
## 383 0.990     (nm)
## 131 0.725      (i)
## 301 1.000     (nm)
## 333 0.683      (i)
## 360 0.999     (nm)
## 578 0.249      (i)
## 271 0.328      (i)
## 499 1.000     (nm)
## 512 1.000     (nm)
## 209 0.662      (i)
## 634 1.000     (nm)
## 385 0.999     (nm)
```

```
## 261 0.389      (i)
## 305 0.964     (nm)
## 121 0.141      (i)
## 208 0.206      (i)
## 596 0.257      (i)
## 331 0.769     (nm)
## 352 0.988     (nm)
## 318 1.000     (nm)
## 196 0.989     (nm)
## 240 0.462      (i)
## 454 0.709      (i)
## 501 0.247      (i)
## 199 0.904     (nm)
## 411 1.000     (nm)
## 593 0.996     (nm)
## 258 0.343      (i)
## 211 0.580      (i)
## 189 0.291      (i)
## 373 1.000     (nm)
## 621 1.000     (nm)
## 602 0.642      (i)
## 417 0.869     (nm)
## 465 0.999     (nm)
## 543 0.841     (nm)
## 369 1.000     (nm)
## 601 0.086      (i)
## 329 0.877     (nm)
## 186 0.164      (i)
## 433 1.000     (nm)
## 615 1.000     (nm)
## 298 1.000     (nm)
## 193 0.998     (nm)
## 524 1.000     (nm)
## 262 0.285      (i)
## 284 0.063      (i)
## 365 1.000     (nm)
## 408 1.000     (nm)
## 414 1.000     (nm)
## 164 0.661      (i)
```

MODEL FIT IMPROVEMENT BY CORRELATING SOME OF THE RESIDUALS
(correlate top 10 indicator residuals)

```
w.modelalt<-
  'persp =~ w1+ w2+ w3+ w4
change=~w5+w6+w7 +w8
humility=~w9+ w10+ w11+ w12
comprres=~w13+ w14+ w15 +w16+ w17
outsider=~w18+w19+ w20+ w21
wow=~persp+change+humility+comprres+outsider
class=~WorriedMoney+Uninsured+OccPres
pclass=~Edu+Income
#residual correlations
w16~~w17
w7~~w8
w13~~w14
w1~~w3
w5~~w6'

fit.alt <- cfa(w.modelalt, data = mrbshort, estimator = "mlr",missing = "fiml")
fitMeasures(fit.alt,  c("chisq", "df", "pvalue", "cfi", "tli",
                        "aic","bic","rmsea","rmsea.ci.lower","rmsea.ci.upper"))
```

```
##          chisq             df         pvalue            cfi            tli
##       1399.226        286.000          0.000          0.951          0.945
##            aic            bic          rmsea rmsea.ci.lower rmsea.ci.upper
##     166611.663     167127.714          0.043          0.040          0.045
```

```
summary(fit.alt, standardized=T,rsquare=T)
```

```
## lavaan (0.5-23.1097) converged normally after 117 iterations
##
##   Number of observations                          2145
##
##   Number of missing patterns                         8
##
##   Estimator                                         ML      Robust
##   Minimum Function Test Statistic             1399.226    1213.434
##   Degrees of freedom                               286         286
##   P-value (Chi-square)                           0.000       0.000
##   Scaling correction factor                                  1.153
##     for the Yuan-Bentler correction
##
## Parameter Estimates:
##
##   Information                                 Observed
##   Standard Errors                   Robust.huber.white
##
## Latent Variables:
##                    Estimate  Std.Err  z-value  P(>|z|)   Std.lv  Std.all
##   persp =~
##     w1                1.000                               0.767    0.679
##     w2                1.043    0.042   24.979    0.000    0.799    0.659
##     w3                1.233    0.032   38.020    0.000    0.946    0.812
##     w4                1.220    0.040   30.208    0.000    0.935    0.785
##   change =~
##     w5                1.000                               0.919    0.774
##     w6                0.955    0.039   24.267    0.000    0.877    0.742
##     w7                0.701    0.041   17.248    0.000    0.644    0.543
##     w8                0.649    0.053   12.337    0.000    0.597    0.497
##   humility =~
##     w9                1.000                               0.876    0.695
##     w10               0.819    0.034   24.124    0.000    0.717    0.513
##     w11               0.966    0.036   26.539    0.000    0.846    0.731
##     w12               0.807    0.040   20.337    0.000    0.707    0.573
##   comprres =~
##     w13               1.000                               0.898    0.743
##     w14               1.068    0.026   41.242    0.000    0.959    0.784
##     w15               1.065    0.031   34.835    0.000    0.957    0.790
##     w16               0.724    0.035   20.571    0.000    0.650    0.537
##     w17               0.732    0.034   21.666    0.000    0.657    0.599
##   outsider =~
##     w18               1.000                               1.063    0.795
##     w19               1.028    0.021   48.186    0.000    1.093    0.837
##     w20               1.096    0.022   50.021    0.000    1.165    0.877
##     w21               0.982    0.026   38.468    0.000    1.044    0.795
##   wow =~
##     persp             1.000                               0.866    0.866
##     change            1.101    0.059   18.708    0.000    0.796    0.796
##     humility          1.096    0.046   23.591    0.000    0.831    0.831
##     comprres          1.216    0.052   23.461    0.000    0.899    0.899
##     outsider          0.770    0.044   17.396    0.000    0.481    0.481
##   class =~
```

```
##     WorriedMoney      1.000                               0.357    0.231
##     Uninsured         5.510    0.833    6.616    0.000    1.967    0.451
##     OccPres          -4.280    0.816   -5.246    0.000   -1.528   -0.795
##   pclass =~
##     Edu               1.000                               0.493    0.404
##     Income            2.112    0.607    3.482    0.000    1.041    0.564
##
## Covariances:
##                    Estimate  Std.Err  z-value  P(>|z|)   Std.lv  Std.all
##  .w16 ~~
##    .w17               0.434    0.029   15.113    0.000    0.434    0.484
##  .w7 ~~
##    .w8                0.476    0.036   13.189    0.000    0.476    0.458
##  .w13 ~~
##    .w14               0.184    0.027    6.876    0.000    0.184    0.300
##  .w1 ~~
##    .w3                0.188    0.022    8.512    0.000    0.188    0.334
##  .w5 ~~
##    .w6                0.113    0.057    1.986    0.047    0.113    0.190
##   wow ~~
##     class             0.014    0.007    1.945    0.052    0.061    0.061
##     pclass           -0.021    0.014   -1.509    0.131   -0.063   -0.063
##   class ~~
##     pclass           -0.047    0.016   -2.945    0.003   -0.267   -0.267
##
## Intercepts:
##                    Estimate  Std.Err  z-value  P(>|z|)   Std.lv  Std.all
##    .w1                2.838    0.024  116.500    0.000    2.838    2.515
##    .w2                2.994    0.026  114.334    0.000    2.994    2.469
##    .w3                3.065    0.025  121.866    0.000    3.065    2.631
##    .w4                3.098    0.026  120.493    0.000    3.098    2.602
##    .w5                3.281    0.026  127.953    0.000    3.281    2.763
##    .w6                3.323    0.026  130.202    0.000    3.323    2.811
##    .w7                3.370    0.026  131.587    0.000    3.370    2.841
##    .w8                3.271    0.026  126.109    0.000    3.271    2.723
##    .w9                3.062    0.027  112.630    0.000    3.062    2.432
##    .w10               2.424    0.030   80.084    0.000    2.424    1.735
##    .w11               2.827    0.025  113.184    0.000    2.827    2.444
##    .w12               2.789    0.027  104.743    0.000    2.789    2.262
##    .w13               3.190    0.026  122.228    0.000    3.190    2.639
##    .w14               3.175    0.026  120.170    0.000    3.175    2.595
##    .w15               3.146    0.026  120.298    0.000    3.146    2.598
##    .w16               3.682    0.026  140.785    0.000    3.682    3.040
##    .w17               3.601    0.024  151.962    0.000    3.601    3.281
##    .w18               2.955    0.029  102.326    0.000    2.955    2.209
##    .w19               2.820    0.028  100.064    0.000    2.820    2.161
##    .w20               2.859    0.029   99.655    0.000    2.859    2.152
##    .w21               2.892    0.028  102.074    0.000    2.892    2.204
##    .WorriedMoney     36.039    0.033 1081.723    0.000   36.039   23.290
##    .Uninsured        13.432    0.095  142.110    0.000   13.432    3.082
##    .OccPres          48.920    0.041 1178.953    0.000   48.920   25.456
##    .Edu               3.863    0.029  133.690    0.000    3.863    3.162
##    .Income            3.876    0.044   88.949    0.000    3.876    2.101
##     persp             0.000                               0.000    0.000
```

```
##     change           0.000                                       0.000    0.000
##     humility         0.000                                       0.000    0.000
##     comprres         0.000                                       0.000    0.000
##     outsider         0.000                                       0.000    0.000
##     wow              0.000                                       0.000    0.000
##     class            0.000                                       0.000    0.000
##     pclass           0.000                                       0.000    0.000
##
## Variances:
##                    Estimate  Std.Err  z-value  P(>|z|)   Std.lv  Std.all
##    .w1                0.685    0.030   23.028    0.000    0.685    0.538
##    .w2                0.832    0.033   25.059    0.000    0.832    0.566
##    .w3                0.462    0.025   18.493    0.000    0.462    0.341
##    .w4                0.543    0.026   20.543    0.000    0.543    0.383
##    .w5                0.566    0.049   11.664    0.000    0.566    0.401
##    .w6                0.628    0.078    8.054    0.000    0.628    0.449
##    .w7                0.992    0.042   23.753    0.000    0.992    0.705
##    .w8                1.087    0.043   25.055    0.000    1.087    0.753
##    .w9                0.819    0.038   21.521    0.000    0.819    0.517
##    .w10               1.437    0.044   32.324    0.000    1.437    0.737
##    .w11               0.622    0.030   20.496    0.000    0.622    0.465
##    .w12               1.021    0.040   25.633    0.000    1.021    0.671
##    .w13               0.654    0.034   19.456    0.000    0.654    0.448
##    .w14               0.577    0.034   17.148    0.000    0.577    0.385
##    .w15               0.552    0.030   18.470    0.000    0.552    0.376
##    .w16               1.044    0.035   29.659    0.000    1.044    0.712
##    .w17               0.773    0.030   25.486    0.000    0.773    0.641
##    .w18               0.659    0.032   20.411    0.000    0.659    0.368
##    .w19               0.509    0.032   16.017    0.000    0.509    0.299
##    .w20               0.409    0.028   14.507    0.000    0.409    0.231
##    .w21               0.632    0.036   17.636    0.000    0.632    0.367
##    .WorriedMoney      2.267    0.089   25.414    0.000    2.267    0.947
##    .Uninsured        15.129    0.879   17.207    0.000   15.129    0.796
##    .OccPres           1.358    0.386    3.515    0.000    1.358    0.368
##    .Edu               1.250    0.081   15.381    0.000    1.250    0.837
##    .Income            2.321    0.323    7.191    0.000    2.321    0.681
##     persp             0.147    0.017    8.791    0.000    0.250    0.250
##     change            0.310    0.058    5.354    0.000    0.367    0.367
##     humility          0.237    0.026    9.264    0.000    0.309    0.309
##     comprres          0.155    0.020    7.668    0.000    0.193    0.193
##     outsider          0.869    0.041   21.032    0.000    0.769    0.769
##     wow               0.441    0.030   14.546    0.000    1.000    1.000
##     class             0.127    0.034    3.708    0.000    1.000    1.000
##     pclass            0.243    0.076    3.184    0.001    1.000    1.000
##
## R-Square:
##                    Estimate
##     w1                0.462
##     w2                0.434
##     w3                0.659
##     w4                0.617
##     w5                0.599
##     w6                0.551
##     w7                0.295
```

```
##     w8                 0.247
##     w9                 0.483
##     w10                0.263
##     w11                0.535
##     w12                0.329
##     w13                0.552
##     w14                0.615
##     w15                0.624
##     w16                0.288
##     w17                0.359
##     w18                0.632
##     w19                0.701
##     w20                0.769
##     w21                0.633
##     WorriedMoney       0.053
##     Uninsured          0.204
##     OccPres            0.632
##     Edu                0.163
##     Income             0.319
##     persp              0.750
##     change             0.633
##     humility           0.691
##     comprres           0.807
##     outsider           0.231
```

```
# excellent improvement - very good fit!
```

ADD ESTIMATES OF LATENT VARIABLES TO THE DATASET

```
parameterEstimates(fit.alt,ci=T,rsquare=T)
```

```
##             lhs op          rhs    est    se      z pvalue ci.lower
## 1         persp =~           w1  1.000 0.000     NA     NA    1.000
## 2         persp =~           w2  1.043 0.042 24.979  0.000    0.961
## 3         persp =~           w3  1.233 0.032 38.020  0.000    1.170
## 4         persp =~           w4  1.220 0.040 30.208  0.000    1.141
## 5        change =~           w5  1.000 0.000     NA     NA    1.000
## 6        change =~           w6  0.955 0.039 24.267  0.000    0.878
## 7        change =~           w7  0.701 0.041 17.248  0.000    0.621
## 8        change =~           w8  0.649 0.053 12.337  0.000    0.546
## 9      humility =~           w9  1.000 0.000     NA     NA    1.000
## 10     humility =~          w10  0.819 0.034 24.124  0.000    0.752
## 11     humility =~          w11  0.966 0.036 26.539  0.000    0.895
## 12     humility =~          w12  0.807 0.040 20.337  0.000    0.730
## 13     comprres =~          w13  1.000 0.000     NA     NA    1.000
## 14     comprres =~          w14  1.068 0.026 41.242  0.000    1.017
## 15     comprres =~          w15  1.065 0.031 34.835  0.000    1.005
## 16     comprres =~          w16  0.724 0.035 20.571  0.000    0.655
## 17     comprres =~          w17  0.732 0.034 21.666  0.000    0.666
## 18     outsider =~          w18  1.000 0.000     NA     NA    1.000
## 19     outsider =~          w19  1.028 0.021 48.186  0.000    0.986
## 20     outsider =~          w20  1.096 0.022 50.021  0.000    1.053
## 21     outsider =~          w21  0.982 0.026 38.468  0.000    0.932
## 22          wow =~        persp  1.000 0.000     NA     NA    1.000
## 23          wow =~       change  1.101 0.059 18.708  0.000    0.986
## 24          wow =~     humility  1.096 0.046 23.591  0.000    1.005
## 25          wow =~     comprres  1.216 0.052 23.461  0.000    1.114
## 26          wow =~     outsider  0.770 0.044 17.396  0.000    0.684
## 27        class =~ WorriedMoney  1.000 0.000     NA     NA    1.000
## 28        class =~    Uninsured  5.510 0.833  6.616  0.000    3.877
## 29        class =~      OccPres -4.280 0.816 -5.246  0.000   -5.880
## 30       pclass =~          Edu  1.000 0.000     NA     NA    1.000
## 31       pclass =~       Income  2.112 0.607  3.482  0.000    0.923
## 32          w16 ~~          w17  0.434 0.029 15.113  0.000    0.378
## 33           w7 ~~           w8  0.476 0.036 13.189  0.000    0.405
## 34          w13 ~~          w14  0.184 0.027  6.876  0.000    0.132
## 35           w1 ~~           w3  0.188 0.022  8.512  0.000    0.145
## 36           w5 ~~           w6  0.113 0.057  1.986  0.047    0.002
## 37           w1 ~~           w1  0.685 0.030 23.028  0.000    0.627
## 38           w2 ~~           w2  0.832 0.033 25.059  0.000    0.767
## 39           w3 ~~           w3  0.462 0.025 18.493  0.000    0.413
## 40           w4 ~~           w4  0.543 0.026 20.543  0.000    0.491
## 41           w5 ~~           w5  0.566 0.049 11.664  0.000    0.471
## 42           w6 ~~           w6  0.628 0.078  8.054  0.000    0.475
## 43           w7 ~~           w7  0.992 0.042 23.753  0.000    0.910
## 44           w8 ~~           w8  1.087 0.043 25.055  0.000    1.002
## 45           w9 ~~           w9  0.819 0.038 21.521  0.000    0.745
## 46          w10 ~~          w10  1.437 0.044 32.324  0.000    1.350
## 47          w11 ~~          w11  0.622 0.030 20.496  0.000    0.563
## 48          w12 ~~          w12  1.021 0.040 25.633  0.000    0.943
## 49          w13 ~~          w13  0.654 0.034 19.456  0.000    0.588
## 50          w14 ~~          w14  0.577 0.034 17.148  0.000    0.511
## 51          w15 ~~          w15  0.552 0.030 18.470  0.000    0.493
## 52          w16 ~~          w16  1.044 0.035 29.659  0.000    0.975
```

```
## 53           w17 ~~          w17  0.773 0.030   25.486  0.000    0.713
## 54           w18 ~~          w18  0.659 0.032   20.411  0.000    0.595
## 55           w19 ~~          w19  0.509 0.032   16.017  0.000    0.447
## 56           w20 ~~          w20  0.409 0.028   14.507  0.000    0.353
## 57           w21 ~~          w21  0.632 0.036   17.636  0.000    0.562
## 58   WorriedMoney ~~ WorriedMoney  2.267 0.089   25.414  0.000    2.092
## 59      Uninsured ~~    Uninsured 15.129 0.879   17.207  0.000   13.406
## 60        OccPres ~~      OccPres  1.358 0.386    3.515  0.000    0.601
## 61            Edu ~~          Edu  1.250 0.081   15.381  0.000    1.091
## 62         Income ~~       Income  2.321 0.323    7.191  0.000    1.688
## 63          persp ~~        persp  0.147 0.017    8.791  0.000    0.114
## 64         change ~~       change  0.310 0.058    5.354  0.000    0.196
## 65       humility ~~     humility  0.237 0.026    9.264  0.000    0.187
## 66       comprres ~~     comprres  0.155 0.020    7.668  0.000    0.116
## 67       outsider ~~     outsider  0.869 0.041   21.032  0.000    0.788
## 68            wow ~~          wow  0.441 0.030   14.546  0.000    0.381
## 69          class ~~        class  0.127 0.034    3.708  0.000    0.060
## 70         pclass ~~       pclass  0.243 0.076    3.184  0.001    0.093
## 71            wow ~~        class  0.014 0.007    1.945  0.052    0.000
## 72            wow ~~       pclass -0.021 0.014   -1.509  0.131   -0.048
## 73          class ~~       pclass -0.047 0.016   -2.945  0.003   -0.078
## 74             w1 ~1               2.838 0.024  116.500  0.000    2.790
## 75             w2 ~1               2.994 0.026  114.334  0.000    2.943
## 76             w3 ~1               3.065 0.025  121.866  0.000    3.016
## 77             w4 ~1               3.098 0.026  120.493  0.000    3.048
## 78             w5 ~1               3.281 0.026  127.953  0.000    3.231
## 79             w6 ~1               3.323 0.026  130.202  0.000    3.273
## 80             w7 ~1               3.370 0.026  131.587  0.000    3.320
## 81             w8 ~1               3.271 0.026  126.109  0.000    3.220
## 82             w9 ~1               3.062 0.027  112.630  0.000    3.009
## 83            w10 ~1               2.424 0.030   80.084  0.000    2.364
## 84            w11 ~1               2.827 0.025  113.184  0.000    2.778
## 85            w12 ~1               2.789 0.027  104.743  0.000    2.737
## 86            w13 ~1               3.190 0.026  122.228  0.000    3.139
## 87            w14 ~1               3.175 0.026  120.170  0.000    3.123
## 88            w15 ~1               3.146 0.026  120.298  0.000    3.095
## 89            w16 ~1               3.682 0.026  140.785  0.000    3.631
## 90            w17 ~1               3.601 0.024  151.962  0.000    3.555
## 91            w18 ~1               2.955 0.029  102.326  0.000    2.899
## 92            w19 ~1               2.820 0.028  100.064  0.000    2.764
## 93            w20 ~1               2.859 0.029   99.655  0.000    2.803
## 94            w21 ~1               2.892 0.028  102.074  0.000    2.837
## 95   WorriedMoney ~1              36.039 0.033 1081.723  0.000   35.973
## 96      Uninsured ~1              13.432 0.095  142.110  0.000   13.247
## 97        OccPres ~1              48.920 0.041 1178.953  0.000   48.839
## 98            Edu ~1               3.863 0.029  133.690  0.000    3.807
## 99         Income ~1               3.876 0.044   88.949  0.000    3.791
## 100         persp ~1               0.000 0.000       NA     NA    0.000
## 101        change ~1               0.000 0.000       NA     NA    0.000
## 102      humility ~1               0.000 0.000       NA     NA    0.000
## 103      comprres ~1               0.000 0.000       NA     NA    0.000
## 104      outsider ~1               0.000 0.000       NA     NA    0.000
## 105           wow ~1               0.000 0.000       NA     NA    0.000
## 106         class ~1               0.000 0.000       NA     NA    0.000
```

```
## 107       pclass ~1                0.000 0.000       NA     NA    0.000
## 108           w1 r2            w1  0.462    NA       NA     NA       NA
## 109           w2 r2            w2  0.434    NA       NA     NA       NA
## 110           w3 r2            w3  0.659    NA       NA     NA       NA
## 111           w4 r2            w4  0.617    NA       NA     NA       NA
## 112           w5 r2            w5  0.599    NA       NA     NA       NA
## 113           w6 r2            w6  0.551    NA       NA     NA       NA
## 114           w7 r2            w7  0.295    NA       NA     NA       NA
## 115           w8 r2            w8  0.247    NA       NA     NA       NA
## 116           w9 r2            w9  0.483    NA       NA     NA       NA
## 117          w10 r2           w10  0.263    NA       NA     NA       NA
## 118          w11 r2           w11  0.535    NA       NA     NA       NA
## 119          w12 r2           w12  0.329    NA       NA     NA       NA
## 120          w13 r2           w13  0.552    NA       NA     NA       NA
## 121          w14 r2           w14  0.615    NA       NA     NA       NA
## 122          w15 r2           w15  0.624    NA       NA     NA       NA
## 123          w16 r2           w16  0.288    NA       NA     NA       NA
## 124          w17 r2           w17  0.359    NA       NA     NA       NA
## 125          w18 r2           w18  0.632    NA       NA     NA       NA
## 126          w19 r2           w19  0.701    NA       NA     NA       NA
## 127          w20 r2           w20  0.769    NA       NA     NA       NA
## 128          w21 r2           w21  0.633    NA       NA     NA       NA
## 129 WorriedMoney r2 WorriedMoney  0.053    NA       NA     NA       NA
## 130    Uninsured r2    Uninsured  0.204    NA       NA     NA       NA
## 131      OccPres r2      OccPres  0.632    NA       NA     NA       NA
## 132          Edu r2          Edu  0.163    NA       NA     NA       NA
## 133       Income r2       Income  0.319    NA       NA     NA       NA
## 134        persp r2        persp  0.750    NA       NA     NA       NA
## 135       change r2       change  0.633    NA       NA     NA       NA
## 136     humility r2     humility  0.691    NA       NA     NA       NA
## 137     comprres r2     comprres  0.807    NA       NA     NA       NA
## 138     outsider r2     outsider  0.231    NA       NA     NA       NA
##     ci.upper
## 1      1.000
## 2      1.125
## 3      1.297
## 4      1.299
## 5      1.000
## 6      1.032
## 7      0.781
## 8      0.752
## 9      1.000
## 10     0.885
## 11     1.038
## 12     0.885
## 13     1.000
## 14     1.119
## 15     1.125
## 16     0.793
## 17     0.798
## 18     1.000
## 19     1.069
## 20     1.139
## 21     1.032
```

```
## 22      1.000
## 23      1.216
## 24      1.187
## 25      1.317
## 26      0.857
## 27      1.000
## 28      7.142
## 29     -2.681
## 30      1.000
## 31      3.301
## 32      0.491
## 33      0.546
## 34      0.237
## 35      0.231
## 36      0.225
## 37      0.744
## 38      0.897
## 39      0.511
## 40      0.595
## 41      0.661
## 42      0.780
## 43      1.074
## 44      1.172
## 45      0.894
## 46      1.524
## 47      0.682
## 48      1.100
## 49      0.720
## 50      0.643
## 51      0.610
## 52      1.113
## 53      0.832
## 54      0.722
## 55      0.571
## 56      0.464
## 57      0.703
## 58      2.442
## 59     16.852
## 60      2.116
## 61      1.409
## 62      2.953
## 63      0.180
## 64      0.423
## 65      0.287
## 66      0.195
## 67      0.950
## 68      0.500
## 69      0.195
## 70      0.393
## 71      0.029
## 72      0.006
## 73     -0.016
## 74      2.886
## 75      3.046
```

```
## 76     3.114
## 77     3.149
## 78     3.331
## 79     3.373
## 80     3.420
## 81     3.322
## 82     3.116
## 83     2.483
## 84     2.876
## 85     2.841
## 86     3.241
## 87     3.227
## 88     3.197
## 89     3.733
## 90     3.648
## 91     3.012
## 92     2.875
## 93     2.915
## 94     2.948
## 95    36.104
## 96    13.618
## 97    49.001
## 98     3.920
## 99     3.962
## 100    0.000
## 101    0.000
## 102    0.000
## 103    0.000
## 104    0.000
## 105    0.000
## 106    0.000
## 107    0.000
## 108       NA
## 109       NA
## 110       NA
## 111       NA
## 112       NA
## 113       NA
## 114       NA
## 115       NA
## 116       NA
## 117       NA
## 118       NA
## 119       NA
## 120       NA
## 121       NA
## 122       NA
## 123       NA
## 124       NA
## 125       NA
## 126       NA
## 127       NA
## 128       NA
## 129       NA
```

```
## 130       NA
## 131       NA
## 132       NA
## 133       NA
## 134       NA
## 135       NA
## 136       NA
## 137       NA
## 138       NA
```

```
summary(lavPredict(fit.alt))
```

```
##      persp              change            humility      
##  Min.   :-1.69955   Min.   :-2.18355   Min.   :-1.8874  
##  1st Qu.:-0.46412   1st Qu.:-0.52480   1st Qu.:-0.5022  
##  Median : 0.02414   Median : 0.04439   Median : 0.0337  
##  Mean   : 0.00000   Mean   : 0.00000   Mean   : 0.0000  
##  3rd Qu.: 0.51733   3rd Qu.: 0.57863   3rd Qu.: 0.5244  
##  Max.   : 1.59258   Max.   : 1.75261   Max.   : 1.9664  
##     comprres           outsider              wow          
##  Min.   :-2.13371   Min.   :-1.74057   Min.   :-1.64460  
##  1st Qu.:-0.53282   1st Qu.:-0.75834   1st Qu.:-0.38452  
##  Median : 0.06237   Median : 0.05843   Median : 0.02187  
##  Mean   : 0.00000   Mean   : 0.00000   Mean   : 0.00000  
##  3rd Qu.: 0.60374   3rd Qu.: 0.78219   3rd Qu.: 0.41912  
##  Max.   : 1.78653   Max.   : 1.93735   Max.   : 1.48426  
##      class              pclass         
##  Min.   :-1.58055   Min.   :-0.772979  
##  1st Qu.:-0.18041   1st Qu.:-0.205624  
##  Median : 0.06595   Median :-0.006738  
##  Mean   : 0.00000   Mean   : 0.000000  
##  3rd Qu.: 0.15325   3rd Qu.: 0.190556  
##  Max.   : 0.74222   Max.   : 1.119645
```

```
estimates<-as.data.frame(lavPredict(fit.alt))#save estimated factor scores
```

add estimates scores to the original dataset

```
mrbclean$wow<-estimates$wow
mrbclean$persp<-estimates$persp
mrbclean$change<-estimates$change
mrbclean$humility<-estimates$humility
mrbclean$comprres<-estimates$comprres
mrbclean$outsider<-estimates$outsider
mrbclean$stateclassr<-estimates$class
mrbclean$stateclass<-mrbclean$stateclassr*(-1)
mrbclean$personalclass<-estimates$pclass

#examine SE of group and within-group variability

W<-ddply(mrbclean[c("State","wow")], "State", transform,
         wowB = mean(wow))
mrbclean$wowB<-unlist(W[c("wowB")])
mrbclean$wowW<-mrbclean$wow-mrbclean$wowB

sd(mrbclean$wowB)/sqrt(length(mrbclean$wowB))
```

```
## [1] 0.001963809
```

```
sd(mrbclean$wowW)/sqrt(length(mrbclean$wowW))
```

```
## [1] 0.0133269
```

obtain individual and state-level effect of social class based on individual-level estimates. hereby, indiv.-level reflects deviation from state average score.

```
d<-ddply(mrbclean[c("State","personalclass")], "State", transform,
         personalclassB = mean(personalclass))
mrbclean$personalclassSTATEAV<-unlist(d[c("personalclassB")])
mrbclean$personalclassDIFFFROMSTATEAV<-mrbclean$personalclass-mrbclean$personalclassSTATEAV
```

EXAMINE REGIONAL-LEVEL EFFECTS by STATES, and across States nested in REGIONS

prepare data first (average scores across states, such that state is the unit of analysis)

```
mrbcleanSmall <- subset(mrbclean, filter.dup=="0"|filter.dup=="NA",
select=c(StateString,SAT,wow,persp,humility,comprres,
change,outsider,stateclass,personalclass,Population,State,Region,OccPres))
mrbcleanSmall$StateString<-strtrim(mrbcleanSmall$StateString, 20)
estimatesReg<-as.data.frame(mrbcleanSmall %>%
    group_by(StateString) %>% summarise_all(funs(mean)))
mrbcleanSmall$StateString<-strtrim(mrbcleanSmall$StateString, 20)
countstate<-as.data.frame(dplyr::count(mrbcleanSmall, StateString))
estimateState<-full_join(estimatesReg, countstate, by = "StateString")

estimateState$Region <- factor(estimateState$Region,
levels = c(4,7,6,5,8,3,1,2),
labels = c("Far West","Great Lakes", "Mideast", "New England",
           "Plains","Rocky Mountains","Southeast","Southwest"))
```

examine how many participants are in each state and how the count varies by size of the state

```
table_score<-as.data.frame(estimateState[c("StateString","n","Population")])
```

Participant Number by State

```
table_score
```

```
##             StateString   n Population
## 1  alabama               23    4849377
## 2  arizona               39    6731484
## 3  arkansas              17    2966369
## 4  california           204   38802500
## 5  colorado              31    5355866
## 6  connecticut           19    3596677
## 7  delaware               9     935614
## 8  district of columbia  10     658893
## 9  florida              141   19893297
## 10 georgia               81   10097343
## 11 idaho                 11    1634464
## 12 illinois              82   12880580
## 13 indiana               40    6596855
## 14 iowa                  22    3107126
## 15 kansas                54    2904021
## 16 kentucky              40    4413457
## 17 louisiana             21    4649676
## 18 maine                  7    1330089
## 19 maryland              42    5976407
## 20 massachusetts         52    6745408
## 21 michigan              87    9909877
## 22 minnesota             38    5457173
## 23 mississippi           12    2994079
## 24 missouri              54    6063589
## 25 montana               10    1023579
## 26 nebraska              16    1881503
## 27 nevada                23    2839099
## 28 new hampshire         15    1326813
## 29 new jersey            56    8938175
## 30 new mexico            14    2085572
## 31 new york             145   19746227
## 32 north carolina        89    9943964
## 33 north dakota           1     739482
## 34 ohio                  99   11594163
## 35 oklahoma              18    3878051
## 36 oregon                24    3970239
## 37 pennsylvania         107   12787209
## 38 rhode island           7    1055173
## 39 south carolina        26    4832482
## 40 south dakota           2     853175
## 41 tennessee             51    6549352
## 42 texas                116   26956958
## 43 utah                  13    2942902
## 44 vermont                5     626562
## 45 virginia              69    8326289
## 46 washington            36    7061530
## 47 west virginia         13    1850326
## 48 wisconsin             53    5757564
## 49 wyoming                1     584153
```

```
summary(table_score$n)
```

```
##    Min. 1st Qu.  Median    Mean 3rd Qu.    Max. 
##    1.00   13.00   26.00   43.78   54.00  204.00
```

most states have at least 10 datapoints, exceptions: delaware, maine, N & S. dakota, rhode island, vermont * wyoming - smaller states

```
cor(estimateState$Population,estimateState$n,method = c("pearson"))
```

```
## [1] 0.9440492
```

```
#r = .94 # population size very closely correlated to the number of participants per state
cor(estimateState$Population,estimateState$n,method = c("kendall"))
```

```
## [1] 0.8298544
```

```
#when examining ordinal correlations, tau = .83==> very high!

ggplot()+geom_point(data=estimateState, aes(x=n, y=StateString,color="Participants",
 shape="Participants"))+ labs(x="Size",y="State")+theme_light()+
  geom_point(data=estimateState, aes(x=Population/100000,y=StateString,
                                     color="Population/100K",shape="Population/100K"))+
  scale_colour_manual(values=c("Participants"="#d95f02", "Population/100K"="#7570b3"))+
scale_shape_discrete()+geom_vline(xintercept=c(15), linetype="dotted")+
  theme(legend.justification=c(.97,0), legend.position=c(.98,.44))+theme(legend.title=element_bl
ank())
```

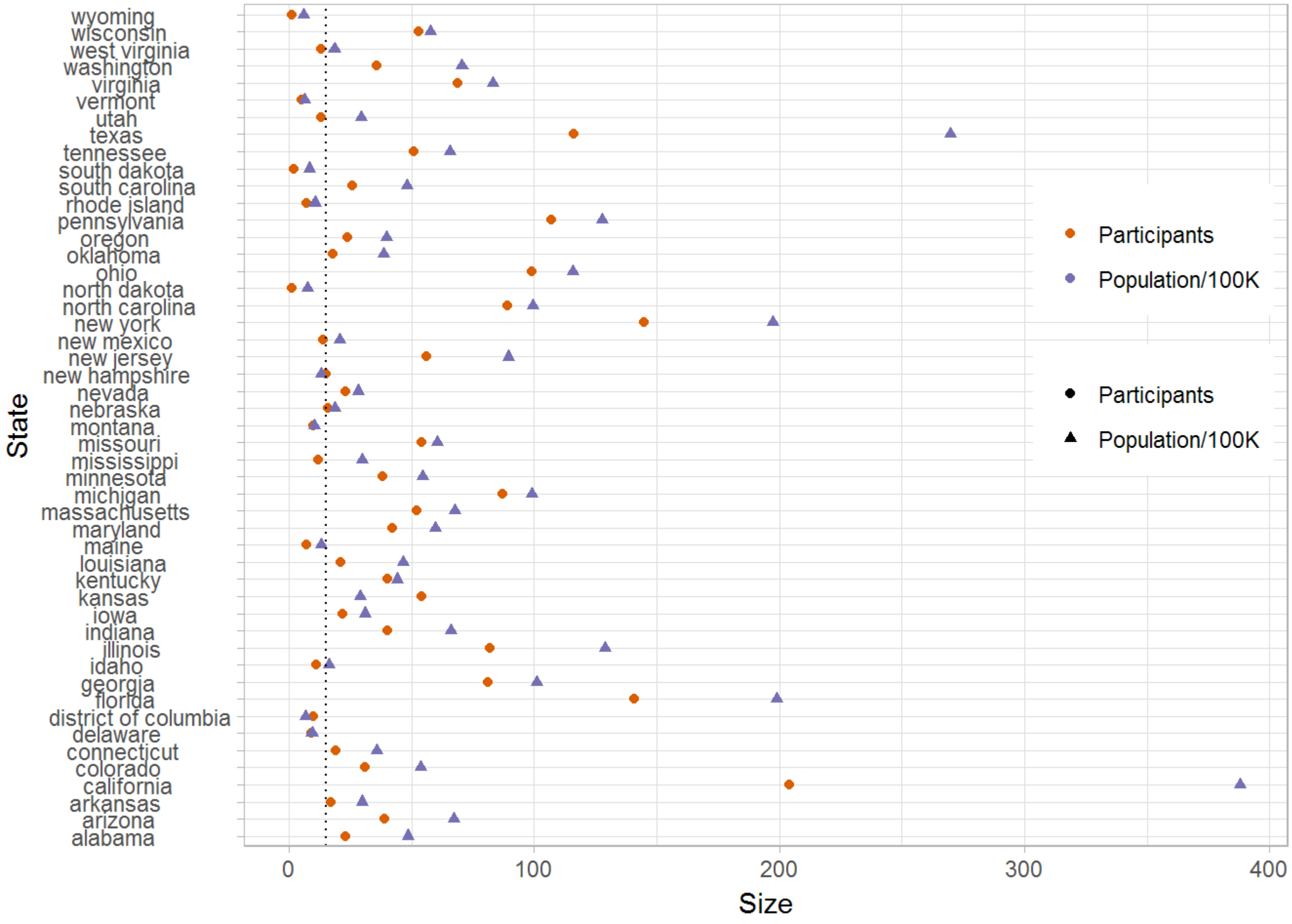

reduce state-specific sample, so that only states with 25+ participants/state included

(to ensure sufficient N per state).

```
estimatesStateclean <- estimateState[ which(estimateState$n>24) , ]
cor(estimatesStateclean$stateclass,estimatesStateclean$wow)
```

```
## [1] -0.3907578
```

```
cor(estimatesStateclean$stateclass,estimatesStateclean$persp)
```

```
## [1] -0.2968698
```

```
cor(estimatesStateclean$stateclass,estimatesStateclean$humility)
```

```
## [1] -0.367001
```

```
cor(estimatesStateclean$stateclass,estimatesStateclean$comprres)
```

```
## [1] -0.2707067
```

```
cor(estimatesStateclean$stateclass,estimatesStateclean$change)
```

```
## [1] -0.2783828
```

```
cor(estimatesStateclean$stateclass,estimatesStateclean$outsider)
```

```
## [1] -0.5224024
```

strongest effect for outsider perspective, weakest effect for change/compromise+resolution. All state-level effects are below r=-.27

```
ggplot(estimatesStateclean, aes(x=stateclass, y=wow)) +
  geom_point(shape=19) +
  geom_smooth(color="black",method=lm)+  # Add linear regression line
  geom_rug(sides = "bl")+
  theme_classic() +labs(x = "Status of the State (z-score)",
                        y = "Wise Reasoning Index (z-score)")
```

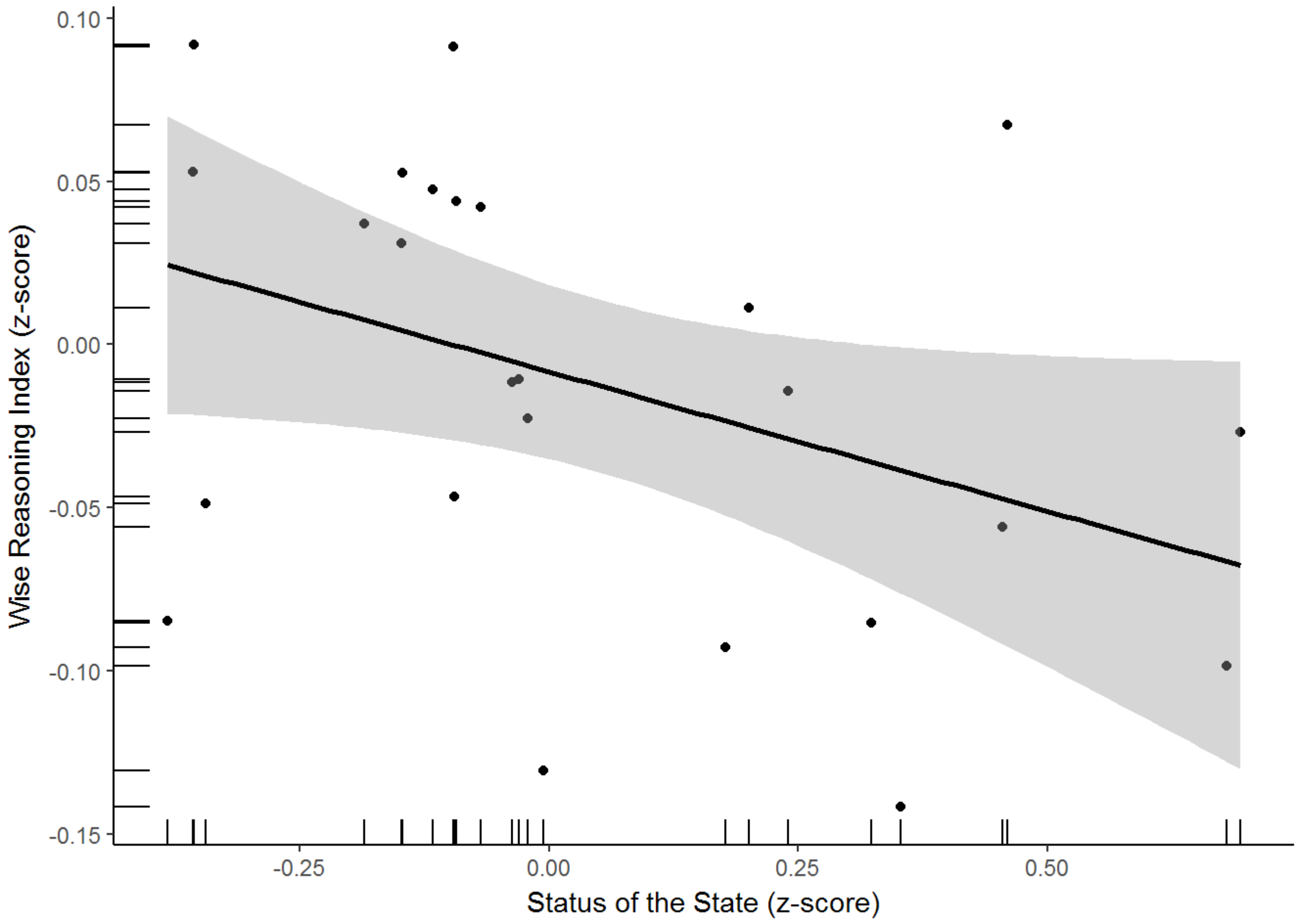

```
ggplot(estimatesStateclean, aes(x=stateclass, y=persp)) +
  geom_point(shape=19) +    # Use hollow circles
  geom_smooth(color="black",method=lm)+  # Add linear regression line
  geom_rug(sides = "bl")+
  theme_classic() +labs(x = "Status of the State  (z-score)",
  y = "Consideration of Others' Perspectives (z-score)")
```

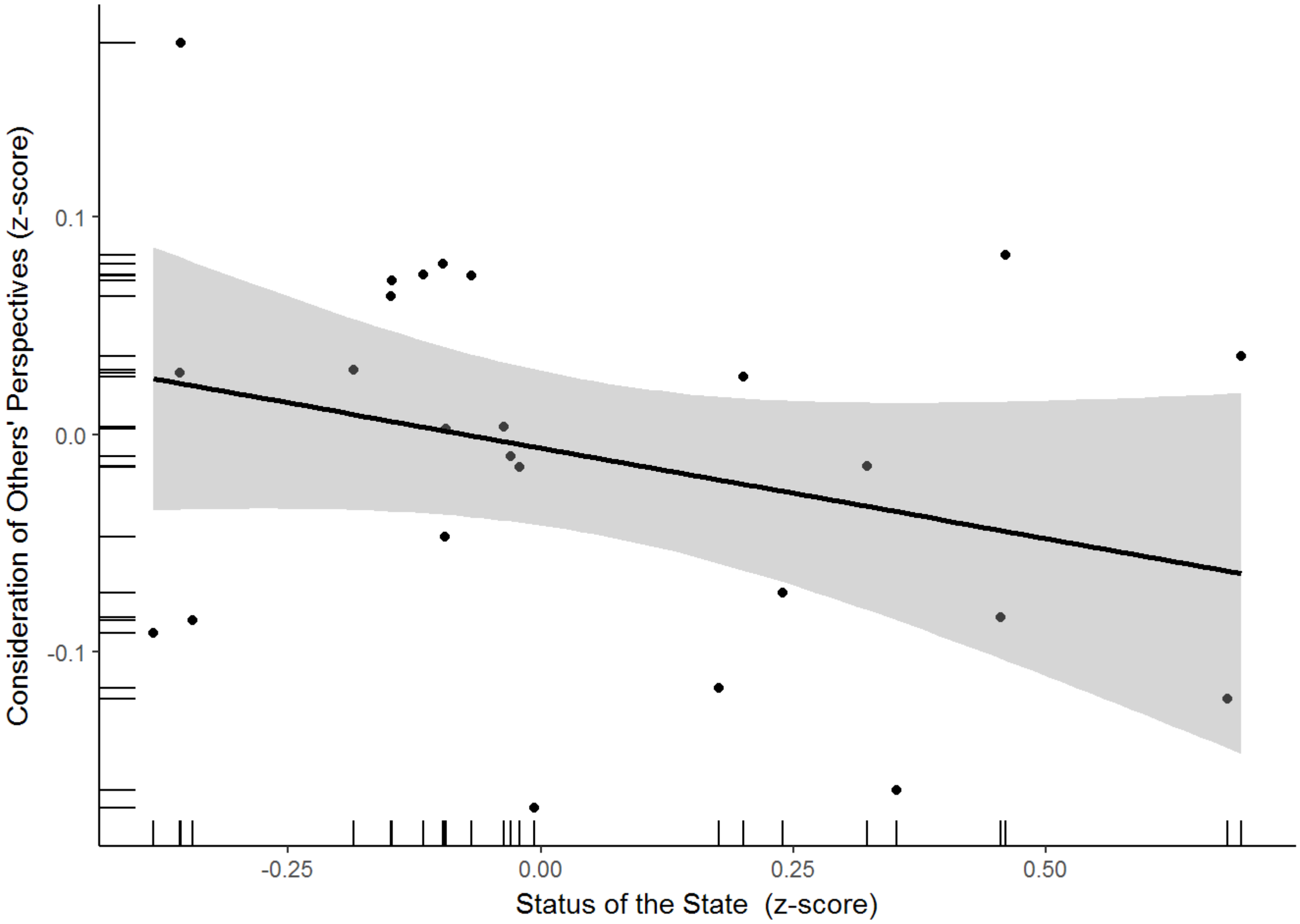


```
ggplot(estimatesStateclean, aes(x=stateclass, y=change)) +
  geom_point(shape=19) +    # Use hollow circles
  geom_smooth(color="black",method=lm)+  # Add linear regression line
  geom_rug(sides = "bl")+
  theme_classic() +labs(x = "Status of the State  (z-score)",
                        y = "Recognition of Change (z-score)")
```

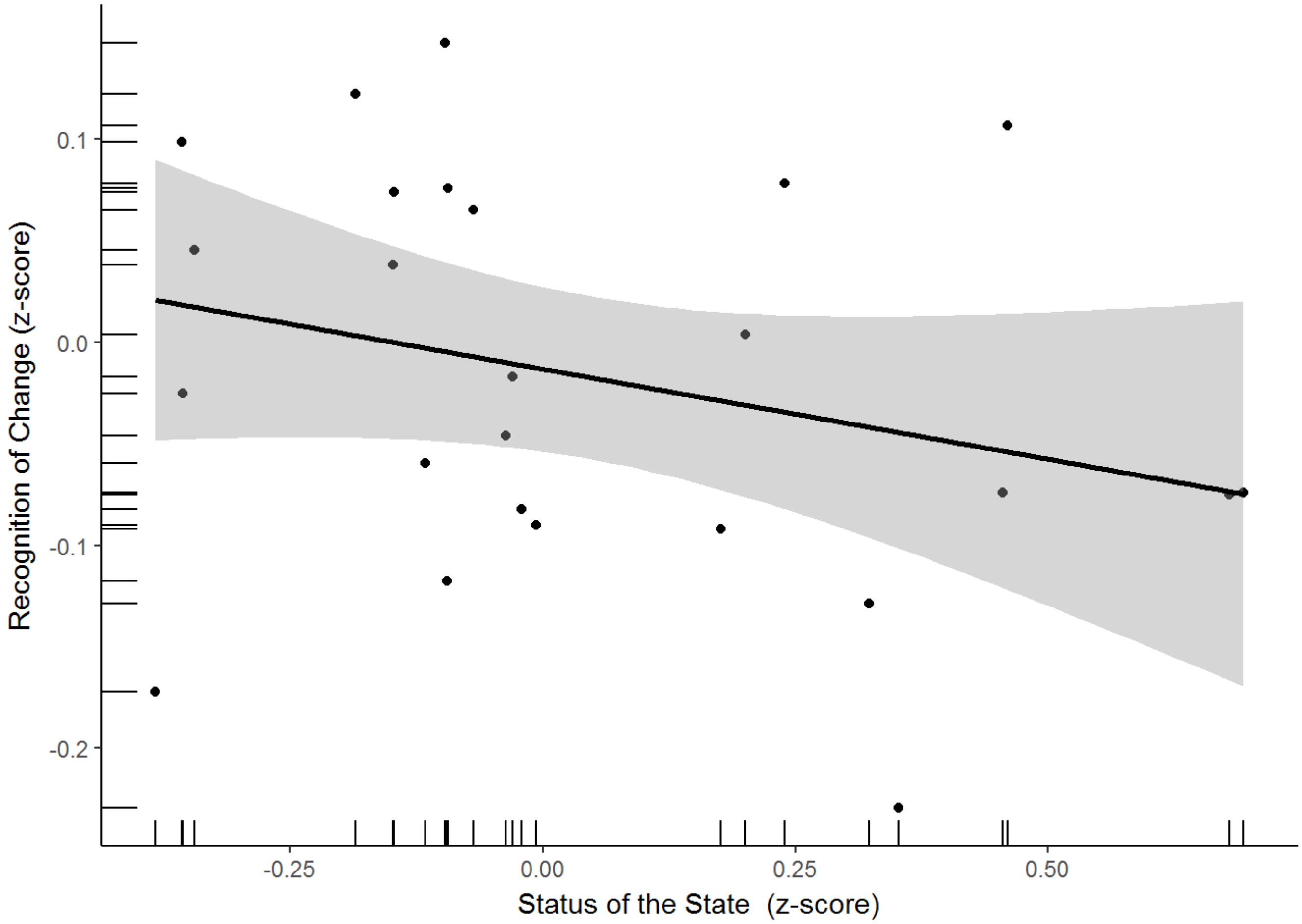


```
ggplot(estimatesStateclean, aes(x=stateclass, y=humility)) +
  geom_point(shape=19) +    # Use hollow circles
  geom_smooth(color="black",method=lm)+  # Add linear regression line
  geom_rug(sides = "bl")+
  theme_classic() +labs(x = "Status of the State  (z-score)",
                      y = "Intellectual Humility (z-score)")
```

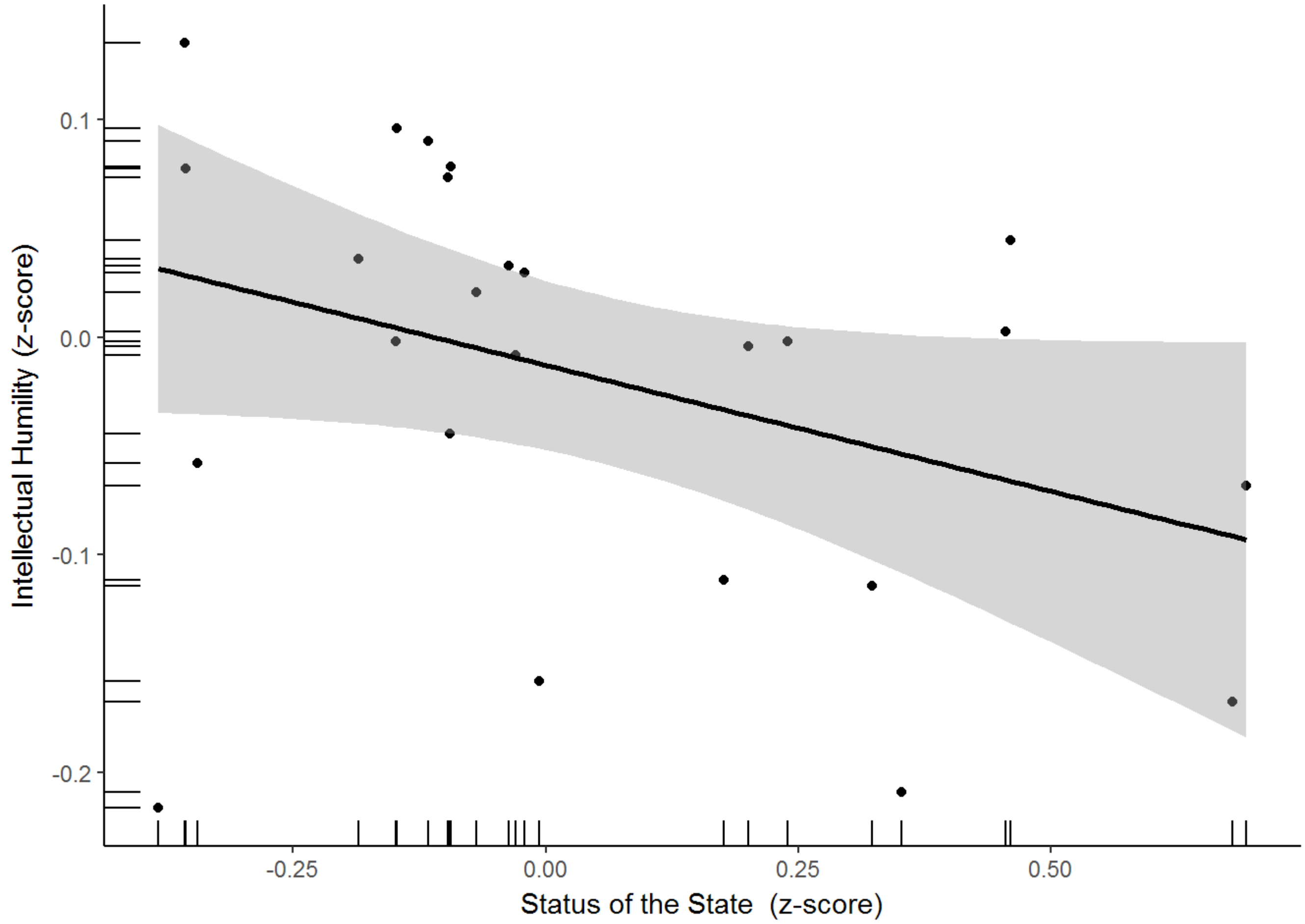


```
ggplot(estimatesStateclean, aes(x=stateclass, y=comprres)) +
  geom_point(shape=19) +    # Use hollow circles
  geom_smooth(color="black",method=lm)+  # Add linear regression line
  geom_rug(sides = "bl")+
  theme_classic() +labs(x = "States: High to Low Status (z-score)",
                        y = "Search for Compromise/Resolution (z-score)")
```

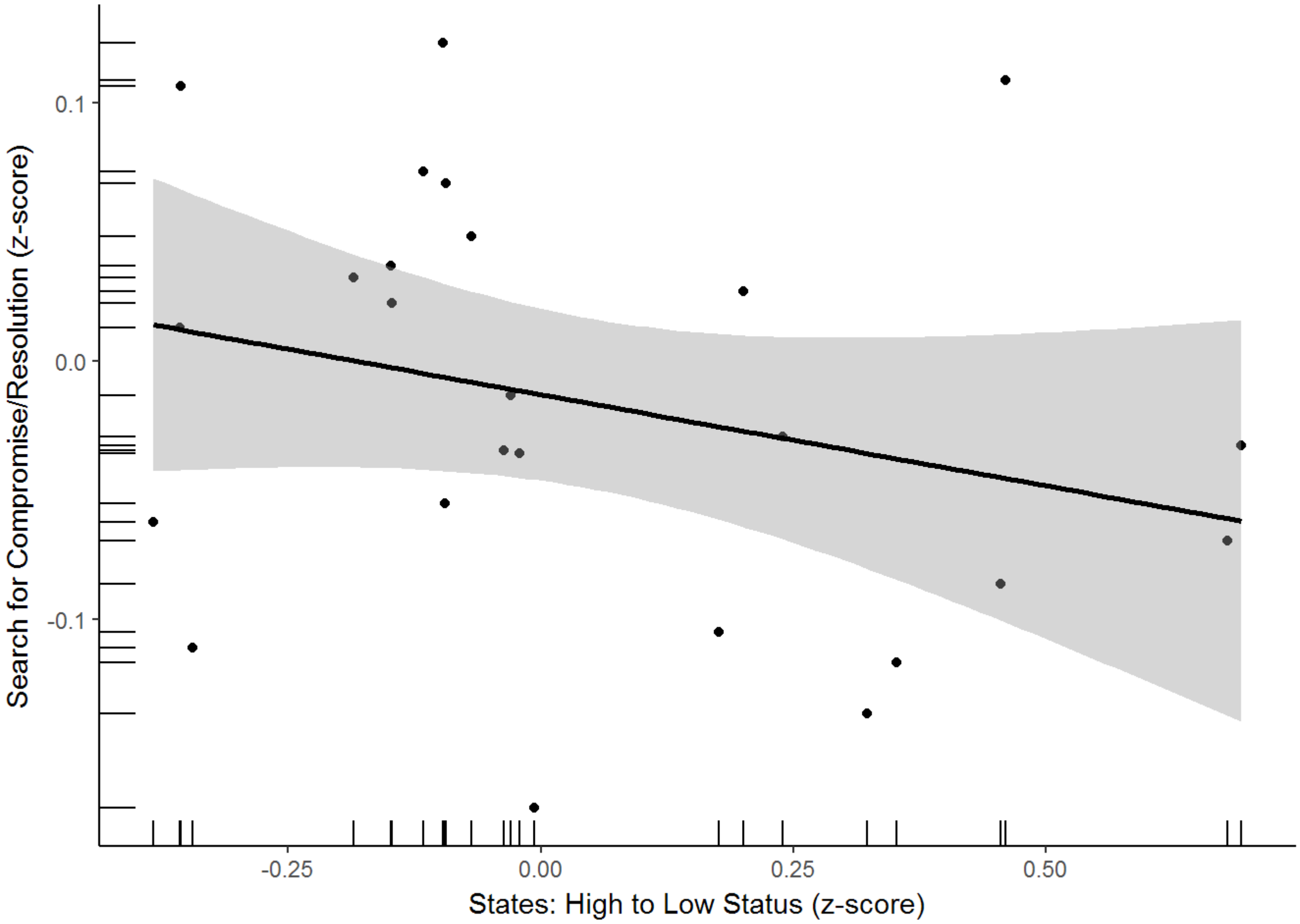


```
ggplot(estimatesStateclean, aes(x=stateclass, y=outsider)) +
  geom_point(shape=19) +    # Use hollow circles
  geom_smooth(color="black",method=lm)+  # Add linear regression line
  geom_rug(sides = "bl")+
  theme_classic() +labs(x = "Status of the State  (z-score)",
                      y = "Outsider's Viewpoint (z-score)")
```

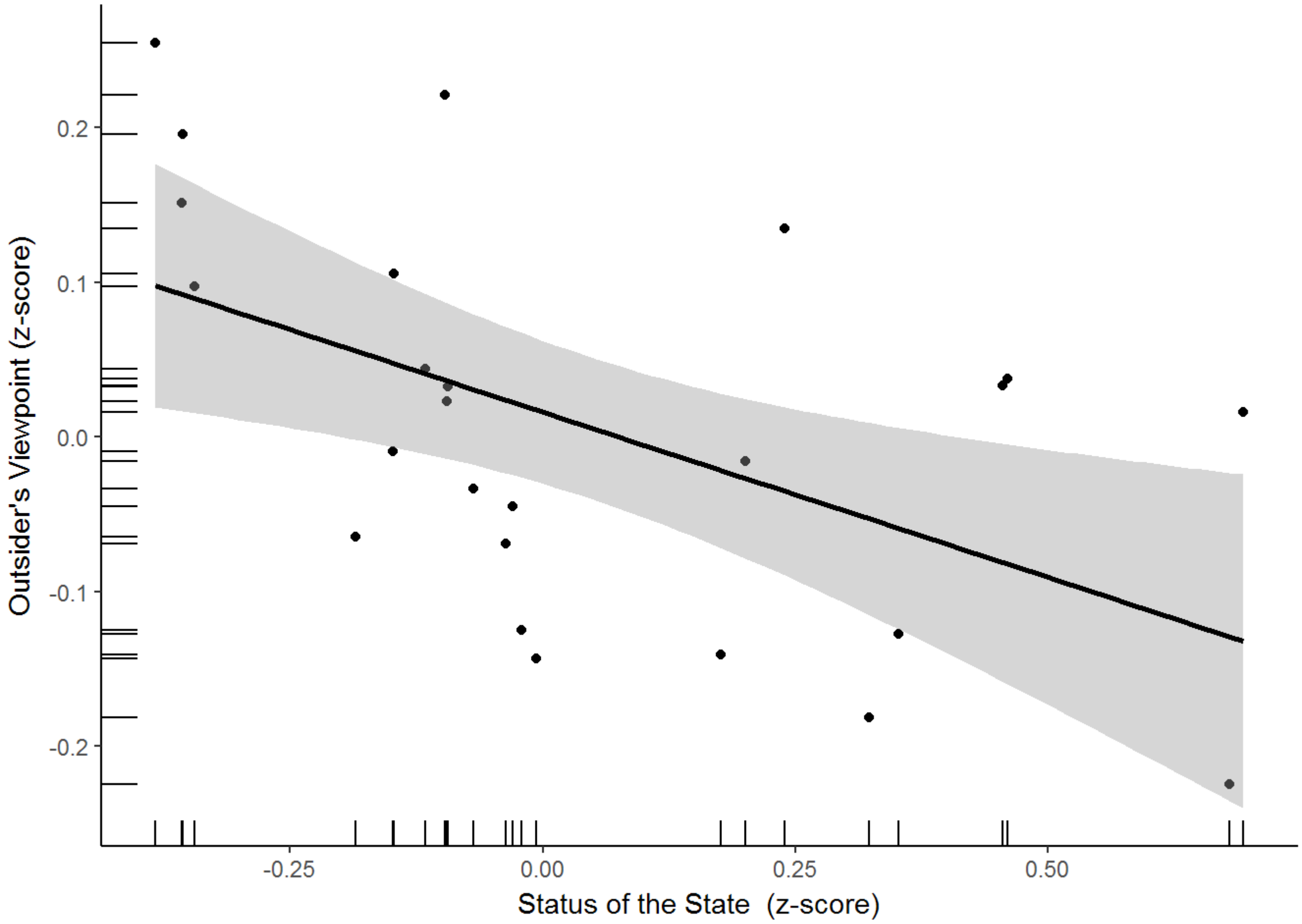

what is the correlation between OccPres index alone and wise reasoning?

```
cor(estimatesStateclean$OccPres,estimatesStateclean$wow)
```

```
## [1] -0.3390648
```

```
cor(estimatesStateclean$OccPres,estimatesStateclean$persp)
```

```
## [1] -0.2628904
```

```
cor(estimatesStateclean$OccPres,estimatesStateclean$humility)
```

```
## [1] -0.3321925
```

```
cor(estimatesStateclean$OccPres,estimatesStateclean$comprres)
```

```
## [1] -0.2090052
```

```
cor(estimatesStateclean$OccPres,estimatesStateclean$change)
```

```
## [1] -0.2307258
```

```
cor(estimatesStateclean$OccPres,estimatesStateclean$outsider)
```

```
## [1] -0.4722589
```

```
#all estimates in the .21-47 range, comparable. weakest for compromise and strongest for outside
r perspective
```

what is the correlation between state-level estimates of status and state-level aggregated person-level estimates?

```
cor(estimatesStateclean$stateclass,estimatesStateclean$personalclass)
```

```
## [1] 0.9550075
```

```
#r = .9 ; very high!
```

replication with person’s own estimates - not just effects of general estimates

# of state’s social status, but also participant-specific estimates

```
ggplot(estimatesStateclean, aes(x=personalclass, y=wow)) +
  geom_point(shape=19) +    # Use hollow circles
  geom_smooth(color="black",method=lm)+  # Add linear regression line
  geom_rug(sides = "bl")+
  theme_classic() +labs(x = "Person-estimated Status of the State (z-score)",
                        y = "Wise Reasoning Index (z-score)")
```

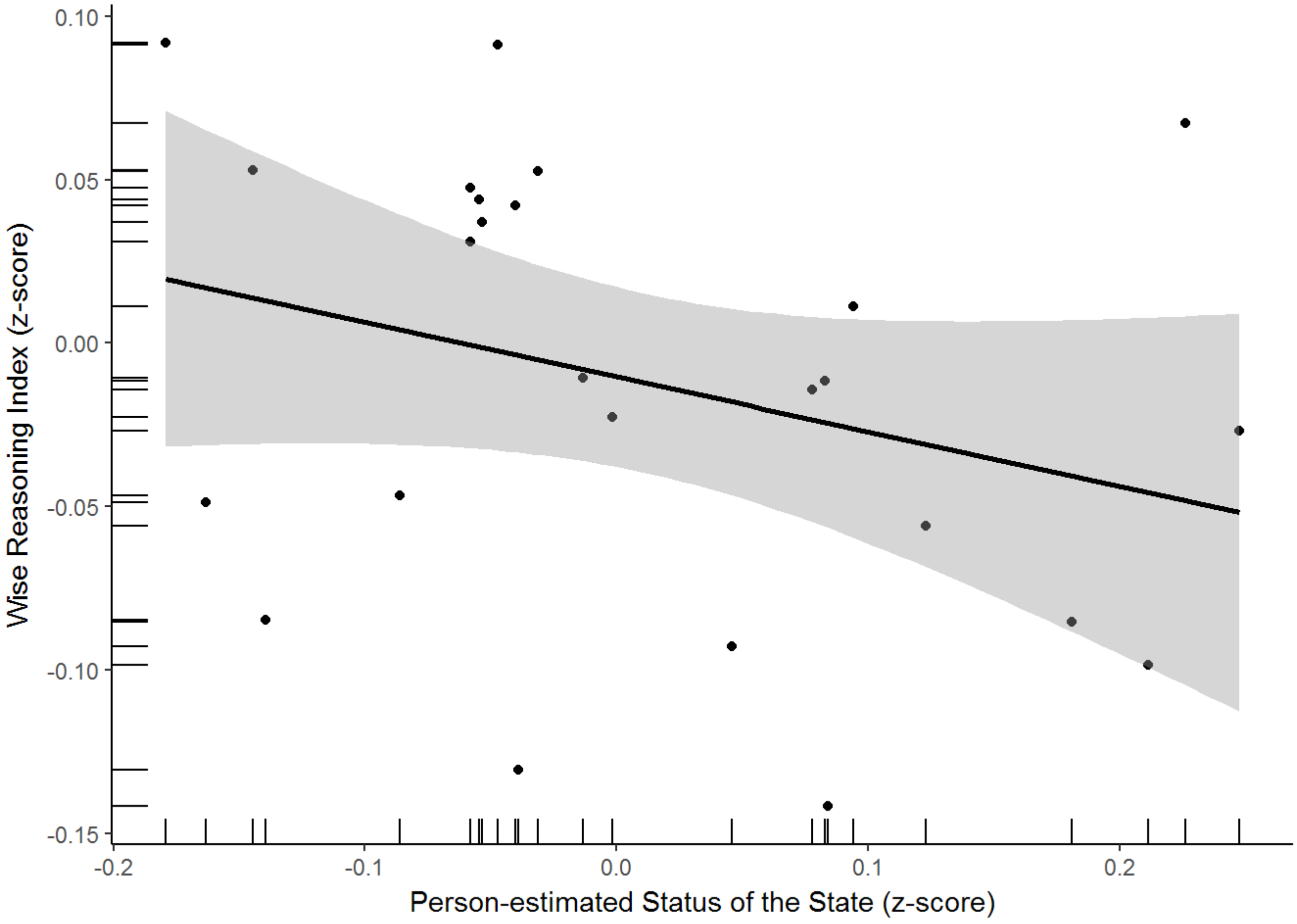

re-run correlations with state-level aggregates of participants’ own estimates of status

```
cor(estimatesStateclean$personalclass,estimatesStateclean$wow) #cor = -.30
```

```
## [1] -0.3012466
```

```
cor(estimatesStateclean$personalclass,estimatesStateclean$persp) #cor = -.19
```

```
## [1] -0.1868936
```

```
cor(estimatesStateclean$personalclass,estimatesStateclean$humility) #cor = -.28
```

```
## [1] -0.281258
```

```
cor(estimatesStateclean$personalclass,estimatesStateclean$comprres) #cor = -.21
```

```
## [1] -0.2070563
```

```
cor(estimatesStateclean$personalclass,estimatesStateclean$change) #cor = -.20
```

```
## [1] -0.2035508
```

```
cor(estimatesStateclean$personalclass,estimatesStateclean$outsider) #cor = -.52
```

```
## [1] -0.5264502
```

```
############################################################
```

REPLICATE WITH different cut-offs - 10 and 15

```
############################################################
```

reduce state-specific sample, so that only states with 10+ participants per state are included.

```
estimatesStateclean10 <- estimateState[ which(estimateState$n>10) , ]
cor(estimatesStateclean10$stateclass,estimatesStateclean10$wow)
```

```
## [1] -0.3919163
```

```
cor(estimatesStateclean10$stateclass,estimatesStateclean10$persp)
```

```
## [1] -0.3590248
```

```
cor(estimatesStateclean10$stateclass,estimatesStateclean10$humility)
```

```
## [1] -0.347325
```

```
cor(estimatesStateclean10$stateclass,estimatesStateclean10$comprres)
```

```
## [1] -0.3641408
```

```
cor(estimatesStateclean10$stateclass,estimatesStateclean10$change)
```

```
## [1] -0.3177701
```

```
cor(estimatesStateclean10$stateclass,estimatesStateclean10$outsider)
```

```
## [1] -0.4125891
```

strongest effect for outsider perspective, weakest effect for change. All state-level effects are below r=-.30!

```
#####################
```

with 15+

```
#####################
```

reduce state-specific sample, so that only states with 15+ participants per state are included.

```
estimatesStateclean15 <- estimateState[ which(estimateState$n>15) , ]
cor(estimatesStateclean15$stateclass,estimatesStateclean15$wow)
```

```
## [1] -0.3457941
```

```
cor(estimatesStateclean15$stateclass,estimatesStateclean15$persp)
```

```
## [1] -0.279155
```

```
cor(estimatesStateclean15$stateclass,estimatesStateclean15$humility)
```

```
## [1] -0.3032005
```

```
cor(estimatesStateclean15$stateclass,estimatesStateclean15$comprres)
```

```
## [1] -0.2881071
```

```
cor(estimatesStateclean15$stateclass,estimatesStateclean15$change)
```

```
## [1] -0.3178294
```

```
cor(estimatesStateclean15$stateclass,estimatesStateclean15$outsider)
```

```
## [1] -0.3697534
```

strongest effect for outsider perspective, weakest effect for change. All state-level effects are below r=-.27 #replicate results, using mixed effects model, with random intercept (i.e., participants' scores NESTED within their states), including ALL participants

```
state.wow<-lmer(wow ~ stateclass+(1|State), mrbclean, REML=F)
state.persp<-lmer(persp ~ stateclass+(1|State), mrbclean, REML=F)
state.humility<-lmer(humility ~ stateclass+(1|State), mrbclean, REML=F)
state.comprres<-lmer(comprres ~ stateclass+(1|State), mrbclean, REML=F)
state.change<-lmer(change ~ stateclass+(1|State), mrbclean, REML=F)
state.outsider<-lmer(outsider ~ stateclass+(1|State), mrbclean, REML=F)
```

Effect of State-level class on wise reasoning

```
summary(state.wow)
```

```
## Linear mixed model fit by maximum likelihood t-tests use Satterthwaite
##   approximations to degrees of freedom [lmerMod]
## Formula: wow ~ stateclass + (1 | State)
##    Data: mrbclean
##
##      AIC      BIC   logLik deviance df.resid
##   3981.0   4003.7  -1986.5   3973.0     2141
##
## Scaled residuals:
##      Min       1Q   Median       3Q      Max
## -2.71213 -0.63822  0.04342  0.68874  2.46352
##
## Random effects:
##  Groups   Name        Variance Std.Dev.
##  State    (Intercept) 0.0000   0.0000
##  Residual             0.3732   0.6109
## Number of obs: 2145, groups:  State, 49
##
## Fixed effects:
##                       Estimate        Std. Error                df t value
## (Intercept)    0.000000001939    0.013190292240 2145.000000000000   0.000
## stateclass    -0.161423754101    0.045047488250 2145.000000000000  -3.583
##             Pr(>|t|)
## (Intercept) 1.000000
## stateclass  0.000347 ***
## ---
## Signif. codes:  0 '***' 0.001 '**' 0.01 '*' 0.05 '.' 0.1 ' ' 1
##
## Correlation of Fixed Effects:
##            (Intr)
## stateclass 0.000
```

compare individual estimates of occupational prestige alone

```
summary(state.wow<-lmer(wow ~ OccPres+(1|State), mrbclean, REML=F)
)
```

```
## Linear mixed model fit by maximum likelihood t-tests use Satterthwaite
##   approximations to degrees of freedom [lmerMod]
## Formula: wow ~ OccPres + (1 | State)
##    Data: mrbclean
##
##      AIC      BIC   logLik deviance df.resid
##   3988.7   4011.4  -1990.4   3980.7     2141
##
## Scaled residuals:
##      Min       1Q   Median       3Q      Max
## -2.69661 -0.62911  0.04288  0.68348  2.44810
##
## Random effects:
##  Groups   Name        Variance Std.Dev.
##  State    (Intercept) 0.0000   0.000
##  Residual             0.3745   0.612
## Number of obs: 2145, groups:  State, 49
##
## Fixed effects:
##                Estimate  Std. Error          df t value Pr(>|t|)
## (Intercept)    0.761008    0.336628 2145.000000   2.261   0.0239 *
## OccPres       -0.015556    0.006876 2145.000000  -2.262   0.0238 *
## ---
## Signif. codes:  0 '***' 0.001 '**' 0.01 '*' 0.05 '.' 0.1 ' ' 1
##
## Correlation of Fixed Effects:
##         (Intr)
## OccPres -0.999
```

Effect of State-level class on recognition of change/ multiple ways a situation may unfold

```
summary(state.change)
```

```
## Linear mixed model fit by maximum likelihood t-tests use Satterthwaite
##   approximations to degrees of freedom [lmerMod]
## Formula: change ~ stateclass + (1 | State)
##    Data: mrbclean
## 
##      AIC      BIC   logLik deviance df.resid 
##   5233.3   5256.0  -2612.6   5225.3     2141 
## 
## Scaled residuals: 
##      Min       1Q   Median       3Q      Max 
## -2.71655 -0.63932  0.05781  0.70855  2.22678 
## 
## Random effects:
##  Groups   Name        Variance              Std.Dev.     
##  State    (Intercept) 0.0000000000000006119 0.00000002474
##  Residual             0.6690826876016711111 0.81797474753
## Number of obs: 2145, groups:  State, 49
## 
## Fixed effects:
##                       Estimate        Std. Error                df t value
## (Intercept)   -0.000000007719    0.017661445606 2145.000000000000   0.000
## stateclass    -0.190731676776    0.060317371969 2145.000000000000  -3.162
##             Pr(>|t|)   
## (Intercept)  1.00000   
## stateclass   0.00159 **
## ---
## Signif. codes:  0 '***' 0.001 '**' 0.01 '*' 0.05 '.' 0.1 ' ' 1
## 
## Correlation of Fixed Effects:
##            (Intr)
## stateclass 0.000
```

Effect of State-level class on facet of consideration of others’ perspectives

```
summary(state.persp)
```

```
## Linear mixed model fit by maximum likelihood t-tests use Satterthwaite
##   approximations to degrees of freedom [lmerMod]
## Formula: persp ~ stateclass + (1 | State)
##    Data: mrbclean
## 
##      AIC      BIC   logLik deviance df.resid 
##   4618.4   4641.1  -2305.2   4610.4     2141 
## 
## Scaled residuals: 
##      Min       1Q   Median       3Q      Max 
## -2.44666 -0.65678  0.03937  0.72921  2.30582 
## 
## Random effects:
##  Groups   Name        Variance Std.Dev.
##  State    (Intercept) 0.0000   0.0000  
##  Residual             0.5023   0.7087  
## Number of obs: 2145, groups:  State, 49
## 
## Fixed effects:
##                      Estimate        Std. Error                df t value
## (Intercept)    0.000000001552    0.015303078107 2145.000000000000   0.000
## stateclass    -0.168248707663    0.052263074896 2145.000000000000  -3.219
##             Pr(>|t|)   
## (Intercept)   1.0000   
## stateclass    0.0013 **
## ---
## Signif. codes:  0 '***' 0.001 '**' 0.01 '*' 0.05 '.' 0.1 ' ' 1
## 
## Correlation of Fixed Effects:
##            (Intr)
## stateclass 0.000
```

Effect of State-level class on facet of intellectual humility

```
summary(state.humility)
```

```
## Linear mixed model fit by maximum likelihood t-tests use Satterthwaite
##   approximations to degrees of freedom [lmerMod]
## Formula: humility ~ stateclass + (1 | State)
##    Data: mrbclean
##
##      AIC      BIC   logLik deviance df.resid
##   5061.6   5084.2  -2526.8   5053.6     2141
##
## Scaled residuals:
##      Min       1Q   Median       3Q      Max
## -2.43287 -0.65792  0.04542  0.66634  2.56234
##
## Random effects:
##  Groups   Name        Variance              Std.Dev.
##  State    (Intercept) 0.0000000000000004089 0.00000002022
##  Residual             0.6176066824840865666 0.78587955978
## Number of obs: 2145, groups:  State, 49
##
## Fixed effects:
##                      Estimate        Std. Error                df t value
## (Intercept)    0.000000004218    0.016968456715 2145.000000000000   0.000
## stateclass    -0.185001162441    0.057950676195 2145.000000000000  -3.192
##             Pr(>|t|)
## (Intercept)  1.00000
## stateclass   0.00143 **
## ---
## Signif. codes:  0 '***' 0.001 '**' 0.01 '*' 0.05 '.' 0.1 ' ' 1
##
## Correlation of Fixed Effects:
##            (Intr)
## stateclass 0.000
```

Effect of State-level class on facet of compromise/search for resolution

```
summary(state.comprres)
```

```
## Linear mixed model fit by maximum likelihood t-tests use Satterthwaite
##   approximations to degrees of freedom [lmerMod]
## Formula: comprres ~ stateclass + (1 | State)
##    Data: mrbclean
## 
##      AIC      BIC   logLik deviance df.resid 
##   5308.7   5331.4  -2650.3   5300.7     2141 
## 
## Scaled residuals: 
##      Min       1Q   Median       3Q      Max 
## -2.60103 -0.63454  0.06871  0.72993  2.18902 
## 
## Random effects:
##  Groups   Name        Variance Std.Dev.
##  State    (Intercept) 0.000    0.0000  
##  Residual             0.693    0.8325  
## Number of obs: 2145, groups:  State, 49
## 
## Fixed effects:
##                     Estimate        Std. Error                df t value
## (Intercept)    0.000000002426    0.017974586550 2145.000000000000   0.000
## stateclass    -0.185653563396    0.061386810974 2145.000000000000  -3.024
##             Pr(>|t|)   
## (Intercept)  1.00000   
## stateclass   0.00252 **
## ---
## Signif. codes:  0 '***' 0.001 '**' 0.01 '*' 0.05 '.' 0.1 ' ' 1
## 
## Correlation of Fixed Effects:
##            (Intr)
## stateclass 0.000
```

Effect of State-level class on facet of outsider viewpoint

```
summary(state.outsider)
```

```
## Linear mixed model fit by maximum likelihood t-tests use Satterthwaite
##   approximations to degrees of freedom [lmerMod]
## Formula: outsider ~ stateclass + (1 | State)
##    Data: mrbclean
## 
##      AIC      BIC   logLik deviance df.resid 
##   6134.4   6157.1  -3063.2   6126.4     2141 
## 
## Scaled residuals: 
##      Min       1Q   Median       3Q      Max 
## -1.78185 -0.75592  0.04919  0.78204  1.99586 
## 
## Random effects:
##  Groups   Name        Variance Std.Dev.
##  State    (Intercept) 0.000    0.000   
##  Residual             1.018    1.009   
## Number of obs: 2145, groups:  State, 49
## 
## Fixed effects:
##                    Estimate      Std. Error              df t value
## (Intercept)    0.0000000398    0.0217896044 2145.0000000000    0.00
## stateclass    -0.1838421134    0.0744158605 2145.0000000000   -2.47
##             Pr(>|t|)  
## (Intercept)   1.0000  
## stateclass    0.0136 *
## ---
## Signif. codes:  0 '***' 0.001 '**' 0.01 '*' 0.05 '.' 0.1 ' ' 1
## 
## Correlation of Fixed Effects:
##            (Intr)
## stateclass 0.000
```

=>results indicate that for each facet of wise reasoning, state-level class is associated with shifts in wise reasoning #replicate the results using regional (rather than state-specific) nesting, with random slope for each State

```
Region.wow<-lmer(wow ~ stateclass+(1+stateclass|Region), mrbclean, REML=F)
Region.wow1<-lmer(wow ~ stateclass+(1|Region), mrbclean, REML=F)
```

effects of state-class nested in regions, allowing for random intercept of state-level class

```
summary(Region.wow1)
```

```
## Linear mixed model fit by maximum likelihood t-tests use Satterthwaite
##   approximations to degrees of freedom [lmerMod]
## Formula: wow ~ stateclass + (1 | Region)
##    Data: mrbclean
##
##      AIC      BIC   logLik deviance df.resid
##   3981.0   4003.7  -1986.5   3973.0     2141
##
## Scaled residuals:
##      Min       1Q   Median       3Q      Max
## -2.71213 -0.63822  0.04342  0.68874  2.46352
##
## Random effects:
##  Groups   Name        Variance             Std.Dev.
##  Region   (Intercept) 0.000000000000000222 0.0000000149
##  Residual             0.373195271103937964 0.6108971035
## Number of obs: 2145, groups:  Region, 8
##
## Fixed effects:
##                      Estimate        Std. Error                df t value
## (Intercept)    0.000000001939    0.013190292240 2145.000000000000   0.000
## stateclass    -0.161423754101    0.045047488250 2145.000000000000  -3.583
##             Pr(>|t|)
## (Intercept) 1.000000
## stateclass  0.000347 ***
## ---
## Signif. codes:  0 '***' 0.001 '**' 0.01 '*' 0.05 '.' 0.1 ' ' 1
##
## Correlation of Fixed Effects:
##            (Intr)
## stateclass 0.000
```

effects of state-class nested in regions, allowing for random intercept+slope of state-level class

```
summary(Region.wow)
```

```
## Linear mixed model fit by maximum likelihood t-tests use Satterthwaite
##   approximations to degrees of freedom [lmerMod]
## Formula: wow ~ stateclass + (1 + stateclass | Region)
##    Data: mrbclean
##
##      AIC      BIC   logLik deviance df.resid
##   3985.0   4019.0  -1986.5   3973.0     2139
##
## Scaled residuals:
##      Min       1Q   Median       3Q      Max
## -2.71213 -0.63822  0.04342  0.68874  2.46352
##
## Random effects:
##  Groups   Name        Variance                Std.Dev.        Corr
##  Region   (Intercept) 0.000000000000000000000 0.0000000000000
##           stateclass  0.000000000000000000413 0.0000000006427  NaN
##  Residual             0.373195271103938519541 0.6108971035321
## Number of obs: 2145, groups:  Region, 8
##
## Fixed effects:
##                        Estimate        Std. Error                df t value
## (Intercept)    0.000000001939    0.013190292240 2145.000000000000   0.000
## stateclass    -0.161423754101    0.045047488250 2145.000000000000  -3.583
##             Pr(>|t|)
## (Intercept) 1.000000
## stateclass  0.000347 ***
## ---
## Signif. codes:  0 '***' 0.001 '**' 0.01 '*' 0.05 '.' 0.1 ' ' 1
##
## Correlation of Fixed Effects:
##            (Intr)
## stateclass 0.000
```

model fit comparison of random slope+ intercept vs. random intercept models

```
anova(Region.wow1,Region.wow)
```

```
## Data: mrbclean
## Models:
## object: wow ~ stateclass + (1 | Region)
## ..1: wow ~ stateclass + (1 + stateclass | Region)
##        Df  AIC    BIC  logLik deviance Chisq Chi Df Pr(>Chisq)
## object  4 3981 4003.7 -1986.5     3973
## ..1     6 3985 4019.0 -1986.5     3973     0      2          1
```

no significant increment when adding a random slope to the region-specific analyses. it appears that the negative effect of state-level status on wise reasoning remains consistent when nesting state-specific scores within regions

control for population estimates

```
summary(lmer(wow ~ stateclass+scale(Population)+(1|State), mrbclean, REML=FALSE))
```

```
## Linear mixed model fit by maximum likelihood t-tests use Satterthwaite
##   approximations to degrees of freedom [lmerMod]
## Formula: wow ~ stateclass + scale(Population) + (1 | State)
##    Data: mrbclean
##
##      AIC      BIC   logLik deviance df.resid
##   3983.0   4011.4  -1986.5   3973.0     2140
##
## Scaled residuals:
##      Min       1Q   Median       3Q      Max
## -2.71444 -0.63978  0.04257  0.68927  2.46234
##
## Random effects:
##  Groups   Name        Variance Std.Dev.
##  State    (Intercept) 0.0000   0.0000
##  Residual             0.3732   0.6109
## Number of obs: 2145, groups:  State, 49
##
## Fixed effects:
##                             Estimate        Std. Error                df
## (Intercept)          0.000000001938    0.013190271097 2145.000000000000
## stateclass          -0.161217736049    0.045115871277 2145.000000000000
## scale(Population)    0.001095724864    0.013213395783 2145.000000000000
##                   t value Pr(>|t|)
## (Intercept)         0.000  1.00000
## stateclass         -3.573  0.00036 ***
## scale(Population)   0.083  0.93392
## ---
## Signif. codes:  0 '***' 0.001 '**' 0.01 '*' 0.05 '.' 0.1 ' ' 1
##
## Correlation of Fixed Effects:
##             (Intr) sttcls
## stateclass  0.000
## scal(Ppltn) 0.000  0.055
```

control for urbanization estimates

```
summary(lmer(wow ~ stateclass+scale(urbcentr.per)+(1|State), mrbclean, REML=FALSE))
```

```
## Linear mixed model fit by maximum likelihood t-tests use Satterthwaite
##   approximations to degrees of freedom [lmerMod]
## Formula: wow ~ stateclass + scale(urbcentr.per) + (1 | State)
##    Data: mrbclean
##
##      AIC      BIC   logLik deviance df.resid
##   3982.6   4010.9  -1986.3   3972.6     2140
##
## Scaled residuals:
##      Min       1Q   Median       3Q      Max
## -2.71574 -0.63238  0.04537  0.69123  2.47221
##
## Random effects:
##  Groups   Name        Variance Std.Dev.
##  State    (Intercept) 0.0000   0.0000
##  Residual             0.3731   0.6108
## Number of obs: 2145, groups:  State, 49
##
## Fixed effects:
##                               Estimate        Std. Error                df
## (Intercept)             0.000000001936    0.013188917970 2145.000000000000
## stateclass             -0.160890479853    0.045049855891 2145.000000000000
## scale(urbcentr.per)     0.008821650991    0.013194061403 2145.000000000000
##                     t value Pr(>|t|)
## (Intercept)           0.000 1.000000
## stateclass           -3.571 0.000363 ***
## scale(urbcentr.per)   0.669 0.503818
## ---
## Signif. codes:  0 '***' 0.001 '**' 0.01 '*' 0.05 '.' 0.1 ' ' 1
##
## Correlation of Fixed Effects:
##             (Intr) sttcls
## stateclass  0.000
## scl(rbcnt.) 0.000  0.018
```

control for inequality estimates

```
summary(lmer(wow ~ stateclass+scale(gini1415)+(1|State), mrbclean, REML=FALSE))
```

```
## Linear mixed model fit by maximum likelihood t-tests use Satterthwaite
##   approximations to degrees of freedom [lmerMod]
## Formula: wow ~ stateclass + scale(gini1415) + (1 | State)
##    Data: mrbclean
##
##      AIC      BIC   logLik deviance df.resid
##   3983.0   4011.3  -1986.5   3973.0     2140
##
## Scaled residuals:
##      Min       1Q   Median       3Q      Max
## -2.71501 -0.63948  0.04331  0.69137  2.45327
##
## Random effects:
##  Groups   Name        Variance Std.Dev.
##  State    (Intercept) 0.0000   0.0000
##  Residual             0.3732   0.6109
## Number of obs: 2145, groups:  State, 49
##
## Fixed effects:
##                          Estimate        Std. Error                df
## (Intercept)        0.000000001949    0.013190135478 2145.000000000000
## stateclass        -0.163083040191    0.045642389380 2145.000000000000
## scale(gini1415)    0.003018414547    0.013367600765 2145.000000000000
##                 t value Pr(>|t|)
## (Intercept)       0.000 1.000000
## stateclass       -3.573 0.000361 ***
## scale(gini1415)   0.226 0.821378
## ---
## Signif. codes:  0 '***' 0.001 '**' 0.01 '*' 0.05 '.' 0.1 ' ' 1
##
## Correlation of Fixed Effects:
##             (Intr) sttcls
## stateclass   0.000
## scl(gn1415)  0.000 -0.161
```

no interation of state-class and inequality

```
summary(lmer(wow ~ stateclass+scale(gini1415)+stateclass*scale(gini1415)+
               (1|State), mrbclean, REML=FALSE))
```

```
## Linear mixed model fit by maximum likelihood t-tests use Satterthwaite
##   approximations to degrees of freedom [lmerMod]
## Formula:
## wow ~ stateclass + scale(gini1415) + stateclass * scale(gini1415) +
##     (1 | State)
##    Data: mrbclean
##
##      AIC      BIC   logLik deviance df.resid
##   3984.3   4018.4  -1986.2   3972.3     2139
##
## Scaled residuals:
##      Min       1Q   Median       3Q      Max
## -2.70827 -0.63567  0.04294  0.68871  2.45393
##
## Random effects:
##  Groups   Name        Variance Std.Dev.
##  State    (Intercept) 0.0000   0.0000
##  Residual             0.3731   0.6108
## Number of obs: 2145, groups:  State, 49
##
## Fixed effects:
##                              Estimate   Std. Error           df t value
## (Intercept)                -0.0012696    0.0132847 2145.0000000  -0.096
## stateclass                 -0.1680961    0.0460698 2145.0000000  -3.649
## scale(gini1415)            -0.0005948    0.0141182 2145.0000000  -0.042
## stateclass:scale(gini1415)    0.0269367    0.0339044 2145.0000000   0.794
##                            Pr(>|t|)
## (Intercept)                 0.92387
## stateclass                  0.00027 ***
## scale(gini1415)             0.96640
## stateclass:scale(gini1415)  0.42700
## ---
## Signif. codes:  0 '***' 0.001 '**' 0.01 '*' 0.05 '.' 0.1 ' ' 1
##
## Correlation of Fixed Effects:
##             (Intr) sttcls s(1415
## stateclass   0.016
## scl(gn1415)  0.039 -0.107
## sttc:(1415) -0.120 -0.137 -0.322
```

control for social desirability

```
summary(lmer(wow ~ stateclass+BIDRFull+(1|State), mrbclean, REML=FALSE))
```

```
## Linear mixed model fit by maximum likelihood t-tests use Satterthwaite
##   approximations to degrees of freedom [lmerMod]
## Formula: wow ~ stateclass + BIDRFull + (1 | State)
##    Data: mrbclean
##
##      AIC      BIC   logLik deviance df.resid
##   1099.7   1122.0   -544.9   1089.7      632
##
## Scaled residuals:
##     Min      1Q  Median      3Q     Max
## -3.0266 -0.6859  0.1214  0.6946  2.5314
##
## Random effects:
##  Groups   Name        Variance             Std.Dev.
##  State    (Intercept) 0.000000000000001305 0.00000003612
##  Residual             0.323954972160157340 0.56917042453
## Number of obs: 637, groups:  State, 48
##
## Fixed effects:
##                Estimate Std. Error         df t value Pr(>|t|)
## (Intercept)  -0.005570   0.035762 637.000000  -0.156 0.876287
## stateclass   -0.304006   0.079806 637.000000  -3.809 0.000153 ***
## BIDRFull      0.002049   0.006008 637.000000   0.341 0.733262
## ---
## Signif. codes:  0 '***' 0.001 '**' 0.01 '*' 0.05 '.' 0.1 ' ' 1
##
## Correlation of Fixed Effects:
##            (Intr) sttcls
## stateclass -0.044
## BIDRFull   -0.776  0.070
```

effect of state-status on wise reasoning holds when controlling for SAT scores in the state

```
summary(lmer(wow ~ stateclass+SAT+(1|State), mrbclean, REML=FALSE))
```

```
## Linear mixed model fit by maximum likelihood t-tests use Satterthwaite
##   approximations to degrees of freedom [lmerMod]
## Formula: wow ~ stateclass + SAT + (1 | State)
##    Data: mrbclean
##
##      AIC      BIC   logLik deviance df.resid
##   3982.9   4011.2  -1986.4   3972.9     2140
##
## Scaled residuals:
##      Min       1Q   Median       3Q      Max
## -2.72221 -0.63495  0.04026  0.69201  2.45716
##
## Random effects:
##  Groups   Name        Variance Std.Dev.
##  State    (Intercept) 0.0000   0.0000
##  Residual             0.3732   0.6109
## Number of obs: 2145, groups:  State, 49
##
## Fixed effects:
##                  Estimate    Std. Error            df t value Pr(>|t|)
## (Intercept)    0.06330329    0.16012046 2145.00000000   0.395 0.692625
## stateclass    -0.16272792    0.04516564 2145.00000000  -3.603 0.000322 ***
## SAT           -0.00004034    0.00010169 2145.00000000  -0.397 0.691631
## ---
## Signif. codes:  0 '***' 0.001 '**' 0.01 '*' 0.05 '.' 0.1 ' ' 1
##
## Correlation of Fixed Effects:
##            (Intr) sttcls
## stateclass -0.073
## SAT        -0.997  0.073
```

EXAMINE INDIVIDUAL DIFFERENCES IN SOCIAL CLASS EFFECTS - same N as in the State-/regional analyses

```
Pclass<-lmer(wow ~ personalclass+(1|State), mrbclean, REML=FALSE)
```

effect of individual class when controlling for nesting of individual scores in states,

as well as individual slopes within states First, intercept only results

```
summary(Pclass)
```

```
## Linear mixed model fit by maximum likelihood t-tests use Satterthwaite
##   approximations to degrees of freedom [lmerMod]
## Formula: wow ~ personalclass + (1 | State)
##    Data: mrbclean
##
##      AIC      BIC   logLik deviance df.resid
##   3969.8   3992.5  -1980.9   3961.8     2141
##
## Scaled residuals:
##      Min       1Q   Median       3Q      Max
## -2.79170 -0.61873  0.04228  0.70297  2.47202
##
## Random effects:
##  Groups   Name        Variance Std.Dev.
##  State    (Intercept) 0.0000   0.0000
##  Residual             0.3713   0.6093
## Number of obs: 2145, groups:  State, 49
##
## Fixed effects:
##                         Estimate        Std. Error                df
## (Intercept)      0.000000004525    0.013155991479 2145.000000000000
## personalclass   -0.219057566367    0.044615118802 2145.000000000000
##               t value   Pr(>|t|)
## (Intercept)      0.00          1
## personalclass   -4.91 0.00000098 ***
## ---
## Signif. codes:  0 '***' 0.001 '**' 0.01 '*' 0.05 '.' 0.1 ' ' 1
##
## Correlation of Fixed Effects:
##             (Intr)
## personlclss 0.000
```

```
PclassRAND<-lmer(wow ~ personalclass+(1+personalclass|State), mrbclean, REML=FALSE)
```

replicate results with a random slope of individual-level class

```
summary(PclassRAND)
```

```
## Linear mixed model fit by maximum likelihood t-tests use Satterthwaite
##   approximations to degrees of freedom [lmerMod]
## Formula: wow ~ personalclass + (1 + personalclass | State)
##    Data: mrbclean
## 
##      AIC      BIC   logLik deviance df.resid 
##   3973.8   4007.9  -1980.9   3961.8     2139 
## 
## Scaled residuals: 
##      Min       1Q   Median       3Q      Max 
## -2.79170 -0.61873  0.04228  0.70297  2.47202 
## 
## Random effects:
##  Groups   Name          Variance            Std.Dev.     Corr
##  State    (Intercept)   0.00000000000000000 0.0000000000     
##           personalclass 0.00000000000001268 0.0000001126  NaN
##  Residual               0.37125683981170809 0.6093084931     
## Number of obs: 2145, groups:  State, 49
## 
## Fixed effects:
##                        Estimate        Std. Error                df
## (Intercept)      0.000000004525    0.013155991479 2145.000000000000
## personalclass   -0.219057566367    0.044615118802 2145.000000000000
##               t value   Pr(>|t|)    
## (Intercept)      0.00          1    
## personalclass   -4.91 0.00000098 ***
## ---
## Signif. codes:  0 '***' 0.001 '**' 0.01 '*' 0.05 '.' 0.1 ' ' 1
## 
## Correlation of Fixed Effects:
##             (Intr)
## personlclss 0.000
```

no sig effects of the random slope of individual-level class above and beyond simple slope model

```
anova(Pclass,PclassRAND)
```

```
## Data: mrbclean
## Models:
## object: wow ~ personalclass + (1 | State)
## ..1: wow ~ personalclass + (1 + personalclass | State)
##        Df    AIC    BIC  logLik deviance Chisq Chi Df Pr(>Chisq)
## object  4 3969.8 3992.5 -1980.9   3961.8                        
## ..1     6 3973.8 4007.9 -1980.9   3961.8     0      2          1
```

effect of inividual-level class on recognition of change

```
summary(lmer(change ~ personalclass+(1|State), mrbclean, REML=FALSE))
```

```
## Linear mixed model fit by maximum likelihood t-tests use Satterthwaite
##   approximations to degrees of freedom [lmerMod]
## Formula: change ~ personalclass + (1 | State)
##    Data: mrbclean
## 
##      AIC      BIC   logLik deviance df.resid 
##   5216.1   5238.8  -2604.0   5208.1     2141 
## 
## Scaled residuals: 
##      Min       1Q   Median       3Q      Max 
## -2.79898 -0.63753  0.07011  0.71324  2.23807 
## 
## Random effects:
##  Groups   Name        Variance Std.Dev.
##  State    (Intercept) 0.0000   0.0000  
##  Residual             0.6637   0.8147  
## Number of obs: 2145, groups:  State, 49
## 
## Fixed effects:
##                        Estimate        Std. Error                df
## (Intercept)     -0.000000003805    0.017590742429 2145.000000000000
## personalclass   -0.312017496135    0.059654421677 2145.000000000000
##               t value    Pr(>|t|)    
## (Intercept)      0.00           1    
## personalclass   -5.23 0.000000186 ***
## ---
## Signif. codes:  0 '***' 0.001 '**' 0.01 '*' 0.05 '.' 0.1 ' ' 1
## 
## Correlation of Fixed Effects:
##             (Intr)
## personlclss 0.000
```

effect of inividual-level class on intellectual humility

```
summary(lmer(humility ~ personalclass+(1|State), mrbclean, REML=FALSE))
```

```
## Linear mixed model fit by maximum likelihood t-tests use Satterthwaite
##   approximations to degrees of freedom [lmerMod]
## Formula: humility ~ personalclass + (1 | State)
##    Data: mrbclean
## 
##      AIC      BIC   logLik deviance df.resid 
##   5055.7   5078.4  -2523.8   5047.7     2141 
## 
## Scaled residuals: 
##      Min       1Q   Median       3Q      Max 
## -2.49194 -0.64088  0.03387  0.67837  2.53951 
## 
## Random effects:
##  Groups   Name        Variance              Std.Dev.    
##  State    (Intercept) 0.0000000000000005326 0.00000002308
##  Residual             0.6159168885965332629 0.78480372616
## Number of obs: 2145, groups:  State, 49
## 
## Fixed effects:
##                        Estimate        Std. Error                 df
## (Intercept)      0.000000006852    0.016945227665 2145.000000000000
## personalclass   -0.230608665814    0.057465326470 2145.000000000000
##               t value Pr(>|t|)    
## (Intercept)     0.000        1    
## personalclass  -4.013 0.000062 ***
## ---
## Signif. codes:  0 '***' 0.001 '**' 0.01 '*' 0.05 '.' 0.1 ' ' 1
## 
## Correlation of Fixed Effects:
##             (Intr)
## personlclss 0.000
```

effect of inividual-level class on consideration of others’ perspectives

```
summary(lmer(persp ~ personalclass+(1|State), mrbclean, REML=FALSE))
```

```
## Linear mixed model fit by maximum likelihood t-tests use Satterthwaite
##   approximations to degrees of freedom [lmerMod]
## Formula: persp ~ personalclass + (1 | State)
##    Data: mrbclean
##
##      AIC      BIC   logLik deviance df.resid
##   4609.7   4632.4  -2300.8   4601.7     2141
##
## Scaled residuals:
##      Min       1Q   Median       3Q      Max
## -2.50030 -0.64883  0.04575  0.72602  2.31645
##
## Random effects:
##  Groups   Name        Variance Std.Dev.
##  State    (Intercept) 0.0000   0.0000
##  Residual             0.5003   0.7073
## Number of obs: 2145, groups:  State, 49
##
## Fixed effects:
##                       Estimate       Std. Error               df t value
## (Intercept)      0.00000000422    0.01527197688 2145.00000000000   0.000
## personalclass   -0.22664886991    0.05179093221 2145.00000000000  -4.376
##                Pr(>|t|)
## (Intercept)           1
## personalclass 0.0000127 ***
## ---
## Signif. codes:  0 '***' 0.001 '**' 0.01 '*' 0.05 '.' 0.1 ' ' 1
##
## Correlation of Fixed Effects:
##             (Intr)
## personlclss 0.000
```

effect of inividual-level class on compromise/search for resolution

```
summary(lmer(comprres ~ personalclass+(1|State), mrbclean, REML=FALSE))
```

```
## Linear mixed model fit by maximum likelihood t-tests use Satterthwaite
##   approximations to degrees of freedom [lmerMod]
## Formula: comprres ~ personalclass + (1 | State)
##    Data: mrbclean
## 
##      AIC      BIC   logLik deviance df.resid 
##   5299.4   5322.1  -2645.7   5291.4     2141 
## 
## Scaled residuals: 
##     Min      1Q  Median      3Q     Max 
## -2.6618 -0.6305  0.0732  0.7371  2.2042 
## 
## Random effects:
##  Groups   Name        Variance            Std.Dev.    
##  State    (Intercept) 0.00000000000000117 0.0000000342
##  Residual             0.69003559848631668 0.8306838138
## Number of obs: 2145, groups:  State, 49
## 
## Fixed effects:
##                         Estimate        Std. Error                df
## (Intercept)      0.000000005551    0.017935855645 2145.000000000000
## personalclass   -0.261333997919    0.060824783269 2145.000000000000
##               t value  Pr(>|t|)    
## (Intercept)     0.000         1    
## personalclass  -4.297 0.0000181 ***
## ---
## Signif. codes:  0 '***' 0.001 '**' 0.01 '*' 0.05 '.' 0.1 ' ' 1
## 
## Correlation of Fixed Effects:
##             (Intr)
## personlclss 0.000
```

effect of inividual-level class on taking an outsider viewpoint

```
summary(lmer(outsider ~ personalclass+(1|State), mrbclean, REML=FALSE))
```

```
## Linear mixed model fit by maximum likelihood t-tests use Satterthwaite
##   approximations to degrees of freedom [lmerMod]
## Formula: outsider ~ personalclass + (1 | State)
##    Data: mrbclean
## 
##      AIC      BIC   logLik deviance df.resid 
##   6132.2   6154.9  -3062.1   6124.2     2141 
## 
## Scaled residuals: 
##      Min       1Q   Median       3Q      Max 
## -1.79591 -0.74029  0.04463  0.78132  1.98882 
## 
## Random effects:
##  Groups   Name        Variance              Std.Dev.    
##  State    (Intercept) 0.0000000000000008528 0.0000000292
##  Residual             1.0174007802369304798 1.0086628675
## Number of obs: 2145, groups:  State, 49
## 
## Fixed effects:
##                       Estimate       Std. Error               df t value
## (Intercept)      0.00000004215    0.02177872168 2145.00000000000   0.000
## personalclass   -0.21218326987    0.07385686261 2145.00000000000  -2.873
##               Pr(>|t|)   
## (Intercept)    1.00000   
## personalclass  0.00411 **
## ---
## Signif. codes:  0 '***' 0.001 '**' 0.01 '*' 0.05 '.' 0.1 ' ' 1
## 
## Correlation of Fixed Effects:
##             (Intr)
## personlclss 0.000
```

examine unique contribution of state above and beyond individual-level effects of class, as well as interaction

both state-level and individual class independently contribute to wise reasoning, individual effect is stronger

no interaction between state and individual levels

```
summary(lmer(wow ~ personalclassSTATEAV*personalclassDIFFFROMSTATEAV+
               (1|State), mrbclean, REML=FALSE))
```

```
## Linear mixed model fit by maximum likelihood t-tests use Satterthwaite
##   approximations to degrees of freedom [lmerMod]
## Formula: wow ~ personalclassSTATEAV * personalclassDIFFFROMSTATEAV + (1 |
##     State)
##    Data: mrbclean
##
##      AIC      BIC   logLik deviance df.resid
##   3973.0   4007.0  -1980.5   3961.0     2139
##
## Scaled residuals:
##      Min       1Q   Median       3Q      Max
## -2.79625 -0.61162  0.03904  0.70281  2.47166
##
## Random effects:
##  Groups   Name        Variance             Std.Dev.
##  State    (Intercept) 0.0000000000000003311 0.0000000182
##  Residual             0.3711132740177283673 0.6091906713
## Number of obs: 2145, groups:  State, 49
##
## Fixed effects:
##                                                 Estimate  Std. Error
## (Intercept)                                    -0.003348    0.013659
## personalclassSTATEAV                           -0.259168    0.125385
## personalclassDIFFFROMSTATEAV                   -0.220259    0.044640
## personalclassSTATEAV:personalclassDIFFFROMSTATEAV   -0.257445    0.283093
##                                                       df t value
## (Intercept)                                  2145.000000  -0.245
## personalclassSTATEAV                         2145.000000  -2.067
## personalclassDIFFFROMSTATEAV                 2145.000000  -4.934
## personalclassSTATEAV:personalclassDIFFFROMSTATEAV 2145.000000  -0.909
##                                                 Pr(>|t|)
## (Intercept)                                       0.8064
## personalclassSTATEAV                              0.0389 *
## personalclassDIFFFROMSTATEAV                 0.000000867 ***
## personalclassSTATEAV:personalclassDIFFFROMSTATEAV      0.3632
## ---
## Signif. codes:  0 '***' 0.001 '**' 0.01 '*' 0.05 '.' 0.1 ' ' 1
##
## Correlation of Fixed Effects:
##             (Intr) prSTATEAV pDIFFF
## prsnSTATEAV 0.081
## pDIFFFROMST 0.008  0.344
## pSTATEAV:DI 0.270  0.300     0.031
```

```
##########################################################
```

attempt to replicate with separate income and education

```
##########################################################
```

alone, these variables do not predict much however, both effects appear to go in the same direction marginally sig effect for recognition of change.

```
summary(lmer(wow ~ Edu+Income+(1|State), mrbclean, REML=FALSE))
```

```
## Linear mixed model fit by maximum likelihood t-tests use Satterthwaite
##   approximations to degrees of freedom [lmerMod]
## Formula: wow ~ Edu + Income + (1 | State)
##    Data: mrbclean
## 
##      AIC      BIC   logLik deviance df.resid 
##   3248.5   3276.0  -1619.2   3238.5     1790 
## 
## Scaled residuals: 
##      Min       1Q   Median       3Q      Max 
## -2.85450 -0.61841  0.05358  0.68287  2.44493 
## 
## Random effects:
##  Groups   Name        Variance Std.Dev.
##  State    (Intercept) 0.0000   0.0000  
##  Residual             0.3557   0.5964  
## Number of obs: 1795, groups:  State, 48
## 
## Fixed effects:
##               Estimate  Std. Error          df t value Pr(>|t|)  
## (Intercept)    0.114679    0.050725 1795.000000   2.261   0.0239 *
## Edu           -0.007100    0.011827 1795.000000  -0.600   0.5484  
## Income        -0.012437    0.007834 1795.000000  -1.588   0.1125  
## ---
## Signif. codes:  0 '***' 0.001 '**' 0.01 '*' 0.05 '.' 0.1 ' ' 1
## 
## Correlation of Fixed Effects:
##        (Intr) Edu   
## Edu    -0.764       
## Income -0.393 -0.228
```

```
summary(lmer(wow ~ Edu+(1|State), mrbclean, REML=FALSE))
```

```
## Linear mixed model fit by maximum likelihood t-tests use Satterthwaite
##   approximations to degrees of freedom [lmerMod]
## Formula: wow ~ Edu + (1 | State)
##    Data: mrbclean
##
##      AIC      BIC   logLik deviance df.resid
##   3251.8   3273.8  -1621.9   3243.8     1793
##
## Scaled residuals:
##     Min      1Q  Median      3Q     Max
## -2.8291 -0.6192  0.0513  0.6827  2.4343
##
## Random effects:
##  Groups   Name        Variance Std.Dev.
##  State    (Intercept) 0.000    0.0000
##  Residual             0.356    0.5967
## Number of obs: 1797, groups:  State, 48
##
## Fixed effects:
##              Estimate Std. Error         df t value Pr(>|t|)
## (Intercept)    0.08314    0.04666 1797.00000   1.782    0.075 .
## Edu           -0.01131    0.01152 1797.00000  -0.982    0.326
## ---
## Signif. codes:  0 '***' 0.001 '**' 0.01 '*' 0.05 '.' 0.1 ' ' 1
##
## Correlation of Fixed Effects:
##     (Intr)
## Edu -0.953
```

```
summary(lmer(wow ~ Income+(1|State), mrbclean, REML=FALSE))
```

```
## Linear mixed model fit by maximum likelihood t-tests use Satterthwaite
##   approximations to degrees of freedom [lmerMod]
## Formula: wow ~ Income + (1 | State)
##    Data: mrbclean
##
##      AIC      BIC   logLik deviance df.resid
##   3250.2   3272.2  -1621.1   3242.2     1795
##
## Scaled residuals:
##      Min       1Q   Median       3Q      Max
## -2.86439 -0.61062  0.04539  0.68065  2.46190
##
## Random effects:
##  Groups   Name        Variance Std.Dev.
##  State    (Intercept) 0.000    0.0000
##  Residual             0.355    0.5958
## Number of obs: 1799, groups:  State, 48
##
## Fixed effects:
##                Estimate  Std. Error          df t value Pr(>|t|)
## (Intercept)    0.091659    0.032663 1799.000000   2.806  0.00507 **
## Income        -0.013597    0.007612 1799.000000  -1.786  0.07424 .
## ---
## Signif. codes:  0 '***' 0.001 '**' 0.01 '*' 0.05 '.' 0.1 ' ' 1
##
## Correlation of Fixed Effects:
##        (Intr)
## Income -0.903
```

```
summary(lmer(change ~ Edu+Income+(1|State), mrbclean, REML=FALSE))
```

```
## Linear mixed model fit by maximum likelihood t-tests use Satterthwaite
##   approximations to degrees of freedom [lmerMod]
## Formula: change ~ Edu + Income + (1 | State)
##    Data: mrbclean
##
##      AIC      BIC   logLik deviance df.resid
##   4280.8   4308.3  -2135.4   4270.8     1790
##
## Scaled residuals:
##      Min       1Q   Median       3Q      Max
## -2.88117 -0.65074  0.05141  0.70707  2.17028
##
## Random effects:
##  Groups   Name        Variance Std.Dev.
##  State    (Intercept) 0.0000   0.0000
##  Residual             0.6322   0.7951
## Number of obs: 1795, groups:  State, 48
##
## Fixed effects:
##               Estimate Std. Error         df t value Pr(>|t|)
## (Intercept)    0.22058    0.06762 1795.00000   3.262  0.00113 **
## Edu           -0.02467    0.01577 1795.00000  -1.565  0.11784
## Income        -0.01825    0.01044 1795.00000  -1.748  0.08071 .
## ---
## Signif. codes:  0 '***' 0.001 '**' 0.01 '*' 0.05 '.' 0.1 ' ' 1
##
## Correlation of Fixed Effects:
##        (Intr) Edu
## Edu    -0.764
## Income -0.393 -0.228
```

```
summary(lmer(persp ~ Edu+Income+(1|State), mrbclean, REML=FALSE))
```

```
## Linear mixed model fit by maximum likelihood t-tests use Satterthwaite
##   approximations to degrees of freedom [lmerMod]
## Formula: persp ~ Edu + Income + (1 | State)
##    Data: mrbclean
## 
##      AIC      BIC   logLik deviance df.resid 
##   3785.9   3813.3  -1887.9   3775.9     1790 
## 
## Scaled residuals: 
##      Min       1Q   Median       3Q      Max 
## -2.56563 -0.65443  0.03585  0.72316  2.25110 
## 
## Random effects:
##  Groups   Name        Variance Std.Dev.
##  State    (Intercept) 0.0000   0.0000  
##  Residual             0.4798   0.6927  
## Number of obs: 1795, groups:  State, 48
## 
## Fixed effects:
##                Estimate  Std. Error          df t value Pr(>|t|)  
## (Intercept)    0.117819    0.058915 1795.000000   2.000   0.0457 *
## Edu           -0.005143    0.013737 1795.000000  -0.374   0.7081  
## Income        -0.012097    0.009099 1795.000000  -1.329   0.1839  
## ---
## Signif. codes:  0 '***' 0.001 '**' 0.01 '*' 0.05 '.' 0.1 ' ' 1
## 
## Correlation of Fixed Effects:
##        (Intr) Edu   
## Edu    -0.764       
## Income -0.393 -0.228
```

```
summary(lmer(humility ~ Edu+Income+(1|State), mrbclean, REML=FALSE))
```

```
## Linear mixed model fit by maximum likelihood t-tests use Satterthwaite
##   approximations to degrees of freedom [lmerMod]
## Formula: humility ~ Edu + Income + (1 | State)
##    Data: mrbclean
##
##      AIC      BIC   logLik deviance df.resid
##   4165.6   4193.0  -2077.8   4155.6     1790
##
## Scaled residuals:
##     Min      1Q  Median      3Q     Max
## -2.5387 -0.6554  0.0192  0.6744  2.5520
##
## Random effects:
##  Groups   Name        Variance              Std.Dev.
##  State    (Intercept) 0.0000000000000006785 0.00000002605
##  Residual             0.5928641607826439230 0.76997672743
## Number of obs: 1795, groups:  State, 48
##
## Fixed effects:
##               Estimate Std. Error         df t value Pr(>|t|)
## (Intercept)    0.07543    0.06549 1795.00000   1.152    0.250
## Edu            0.00406    0.01527 1795.00000   0.266    0.790
## Income        -0.01507    0.01011 1795.00000  -1.490    0.136
##
## Correlation of Fixed Effects:
##        (Intr) Edu
## Edu    -0.764
## Income -0.393 -0.228
```

```
summary(lmer(comprres ~ Edu+Income+(1|State), mrbclean, REML=FALSE))
```

```
## Linear mixed model fit by maximum likelihood t-tests use Satterthwaite
##   approximations to degrees of freedom [lmerMod]
## Formula: comprres ~ Edu + Income + (1 | State)
##    Data: mrbclean
##
##      AIC      BIC   logLik deviance df.resid
##   4383.8   4411.2  -2186.9   4373.8     1790
##
## Scaled residuals:
##      Min       1Q   Median       3Q      Max
## -2.70274 -0.62718  0.07224  0.72593  2.13939
##
## Random effects:
##  Groups   Name        Variance Std.Dev.
##  State    (Intercept) 0.0000   0.0000
##  Residual             0.6695   0.8182
## Number of obs: 1795, groups:  State, 48
##
## Fixed effects:
##                Estimate  Std. Error          df t value Pr(>|t|)
## (Intercept)    0.132115    0.069592 1795.000000   1.898   0.0578 .
## Edu           -0.007751    0.016226 1795.000000  -0.478   0.6329
## Income        -0.012936    0.010748 1795.000000  -1.204   0.2289
## ---
## Signif. codes:  0 '***' 0.001 '**' 0.01 '*' 0.05 '.' 0.1 ' ' 1
##
## Correlation of Fixed Effects:
##        (Intr) Edu
## Edu    -0.764
## Income -0.393 -0.228
```

```
summary(lmer(outsider ~ Edu+Income+(1|State), mrbclean, REML=FALSE))
```

```
## Linear mixed model fit by maximum likelihood t-tests use Satterthwaite
##   approximations to degrees of freedom [lmerMod]
## Formula: outsider ~ Edu + Income + (1 | State)
##    Data: mrbclean
## 
##      AIC      BIC   logLik deviance df.resid 
##   5145.4   5172.8  -2567.7   5135.4     1790 
## 
## Scaled residuals: 
##     Min      1Q  Median      3Q     Max 
## -1.7582 -0.7599  0.0453  0.7835  1.9282 
## 
## Random effects:
##  Groups   Name        Variance Std.Dev.
##  State    (Intercept) 0.000    0.000   
##  Residual             1.023    1.012   
## Number of obs: 1795, groups:  State, 48
## 
## Fixed effects:
##                 Estimate  Std. Error          df t value Pr(>|t|)
## (Intercept)    0.095137    0.086038 1795.000000   1.106    0.269
## Edu           -0.014927    0.020061 1795.000000  -0.744    0.457
## Income        -0.008558    0.013288 1795.000000  -0.644    0.520
## 
## Correlation of Fixed Effects:
##        (Intr) Edu   
## Edu    -0.764       
## Income -0.393 -0.228
```

control for demographics

effect of individual-level social class holds when controlling for gender and age

```
summary(lmer(wow ~ personalclass+scale(Age)+as.factor(Gender)+(1|State), mrbclean, REML=FALSE))
```

```
## Linear mixed model fit by maximum likelihood t-tests use Satterthwaite
##   approximations to degrees of freedom [lmerMod]
## Formula:
## wow ~ personalclass + scale(Age) + as.factor(Gender) + (1 | State)
##    Data: mrbclean
##
##      AIC      BIC   logLik deviance df.resid
##   3969.7   4003.7  -1978.9   3957.7     2138
##
## Scaled residuals:
##      Min       1Q   Median       3Q      Max
## -2.82643 -0.61461  0.05291  0.70006  2.49568
##
## Random effects:
##  Groups   Name        Variance              Std.Dev.
##  State    (Intercept) 0.0000000000000008756 0.00000002959
##  Residual             0.3708602400922935693 0.60898295550
## Number of obs: 2144, groups:  State, 49
##
## Fixed effects:
##                      Estimate  Std. Error          df t value   Pr(>|t|)
## (Intercept)         -0.025378    0.020651 2144.000000  -1.229      0.219
## personalclass       -0.217693    0.045045 2144.000000  -4.833 0.00000144
## scale(Age)          -0.007187    0.013325 2144.000000  -0.539      0.590
## as.factor(Gender)2    0.042432    0.026856 2144.000000   1.580      0.114
##
## (Intercept)
## personalclass      ***
## scale(Age)
## as.factor(Gender)2
## ---
## Signif. codes:  0 '***' 0.001 '**' 0.01 '*' 0.05 '.' 0.1 ' ' 1
##
## Correlation of Fixed Effects:
##             (Intr) prsnlc scl(A)
## personlclss  0.013
## scale(Age)   0.058 -0.139
## as.fctr(G)2 -0.771 -0.016 -0.076
```

control for regional differences in income inequality

main effect of person-level class and no interaction

```
summary(lmer(wow ~ scale(personalclass,scale=F)+scale(gini1415,scale=F)+
               scale(personalclass,scale=F)*scale(gini1415,scale=F)+
               (1|State), mrbclean, REML=F))
```

```
## Linear mixed model fit by maximum likelihood t-tests use Satterthwaite
##   approximations to degrees of freedom [lmerMod]
## Formula:
## wow ~ scale(personalclass, scale = F) + scale(gini1415, scale = F) +
##     scale(personalclass, scale = F) * scale(gini1415, scale = F) +
##     (1 | State)
##    Data: mrbclean
##
##      AIC      BIC   logLik deviance df.resid
##   3973.5   4007.6  -1980.8   3961.5     2139
##
## Scaled residuals:
##      Min       1Q   Median       3Q      Max
## -2.80061 -0.61871  0.04583  0.70274  2.47199
##
## Random effects:
##  Groups   Name        Variance Std.Dev.
##  State    (Intercept) 0.0000   0.0000
##  Residual             0.3712   0.6093
## Number of obs: 2145, groups:  State, 49
##
## Fixed effects:
##                                                              Estimate
## (Intercept)                                                 0.0005981
## scale(personalclass, scale = F)                            -0.2182590
## scale(gini1415, scale = F)                                  0.1341497
## scale(personalclass, scale = F):scale(gini1415, scale = F)   -1.3050357
##                                                            Std. Error
## (Intercept)                                                 0.0132013
## scale(personalclass, scale = F)                             0.0448304
## scale(gini1415, scale = F)                                  0.7589771
## scale(personalclass, scale = F):scale(gini1415, scale = F)    2.4088725
##                                                                    df
## (Intercept)                                              2145.0000000
## scale(personalclass, scale = F)                          2145.0000000
## scale(gini1415, scale = F)                               2145.0000000
## scale(personalclass, scale = F):scale(gini1415, scale = F) 2145.0000000
##                                                          t value
## (Intercept)                                                0.045
## scale(personalclass, scale = F)                           -4.869
## scale(gini1415, scale = F)                                 0.177
## scale(personalclass, scale = F):scale(gini1415, scale = F)  -0.542
##                                                            Pr(>|t|)
## (Intercept)                                                   0.964
## scale(personalclass, scale = F)                          0.00000121 ***
## scale(gini1415, scale = F)                                    0.860
## scale(personalclass, scale = F):scale(gini1415, scale = F)      0.588
## ---
## Signif. codes:  0 '***' 0.001 '**' 0.01 '*' 0.05 '.' 0.1 ' ' 1
##
## Correlation of Fixed Effects:
##             (Intr) s(,s=F s(1s=F
## scl(pr,s=F)  0.004
```

```
## s(1415,s=F)  0.016 -0.078
## s(,s=F):s=F -0.084 -0.045 -0.188
```

control for social desirability

first, is there effect of social class on social desirability? Not really

```
summary(lmer(BIDRFull ~ personalclass+(1|State), mrbclean, REML=FALSE))
```

```
## Linear mixed model fit by maximum likelihood t-tests use Satterthwaite
##   approximations to degrees of freedom [lmerMod]
## Formula: BIDRFull ~ personalclass + (1 | State)
##    Data: mrbclean
## 
##      AIC      BIC   logLik deviance df.resid 
##   3500.8   3518.7  -1746.4   3492.8      633 
## 
## Scaled residuals: 
##     Min      1Q  Median      3Q     Max 
## -1.4010 -0.7259 -0.2525  0.5172  4.0566 
## 
## Random effects:
##  Groups   Name        Variance Std.Dev.
##  State    (Intercept)  0.3129  0.5593 
##  Residual             13.8350  3.7195 
## Number of obs: 637, groups:  State, 48
## 
## Fixed effects:
##               Estimate Std. Error       df t value            Pr(>|t|)
## (Intercept)     4.6816     0.1811  26.0000  25.847 <0.0000000000000002 ***
## personalclass   0.5508     0.4564 621.2000   1.207               0.228    
## ---
## Signif. codes:  0 '***' 0.001 '**' 0.01 '*' 0.05 '.' 0.1 ' ' 1
## 
## Correlation of Fixed Effects:
##             (Intr)
## personlclss 0.080
```

next, including social desirability as control variables does not change the relationship between social class and wise reasoning.

```
summary(lmer(wow ~ personalclass+BIDRFull+(1|State), mrbclean, REML=FALSE))
```

```
## Linear mixed model fit by maximum likelihood t-tests use Satterthwaite
##   approximations to degrees of freedom [lmerMod]
## Formula: wow ~ personalclass + BIDRFull + (1 | State)
##    Data: mrbclean
## 
##      AIC      BIC   logLik deviance df.resid 
##   1098.5   1120.8   -544.3   1088.5      632 
## 
## Scaled residuals: 
##     Min      1Q  Median      3Q     Max 
## -3.1423 -0.6574  0.0796  0.7049  2.3752 
## 
## Random effects:
##  Groups   Name        Variance Std.Dev.
##  State    (Intercept) 0.0000   0.0000  
##  Residual             0.3233   0.5686  
## Number of obs: 637, groups:  State, 48
## 
## Fixed effects:
##                 Estimate Std. Error         df t value  Pr(>|t|)    
## (Intercept)    -0.023932   0.035828 637.000000  -0.668     0.504    
## personalclass  -0.272594   0.068641 637.000000  -3.971 0.0000796 ***
## BIDRFull        0.004568   0.005992 637.000000   0.762     0.446    
## ---
## Signif. codes:  0 '***' 0.001 '**' 0.01 '*' 0.05 '.' 0.1 ' ' 1
## 
## Correlation of Fixed Effects:
##             (Intr) prsnlc
## personlclss  0.087       
## BIDRFull    -0.775 -0.039
```

control for agreeableness, openness, and orientation to others vs. self

(due to possible overlap with some aspects of wise reasoning) both social class and openness explain variance in wise reasoning

```
summary(lmer(wow ~ personalclass+Openness+(1|State), mrbclean, REML=FALSE))
```

```
## Linear mixed model fit by maximum likelihood t-tests use Satterthwaite
##   approximations to degrees of freedom [lmerMod]
## Formula: wow ~ personalclass + Openness + (1 | State)
##    Data: mrbclean
##
##      AIC      BIC   logLik deviance df.resid
##   1373.1   1396.7   -681.6   1363.1      828
##
## Scaled residuals:
##      Min       1Q   Median       3Q      Max
## -2.95935 -0.63228  0.09931  0.69044  2.75501
##
## Random effects:
##  Groups   Name        Variance             Std.Dev.
##  State    (Intercept) 0.000000000000001297 0.00000003601
##  Residual             0.300743766786798816 0.54840110028
## Number of obs: 833, groups:  State, 48
##
## Fixed effects:
##                Estimate Std. Error        df t value    Pr(>|t|)
## (Intercept)    -0.80633    0.15737 833.00000  -5.124 0.000000372 ***
## personalclass  -0.25381    0.05813 833.00000  -4.366 0.000014242 ***
## Openness        0.20552    0.03968 833.00000   5.180 0.000000278 ***
## ---
## Signif. codes:  0 '***' 0.001 '**' 0.01 '*' 0.05 '.' 0.1 ' ' 1
##
## Correlation of Fixed Effects:
##             (Intr) prsnlc
## personlclss  0.025
## Openness    -0.993 -0.019
```

both social class and agreeableness explain variance in wise reasoning

```
summary(lmer(wow ~ personalclass+Agreeableness+(1|State), mrbclean, REML=FALSE))
```

```
## Linear mixed model fit by maximum likelihood t-tests use Satterthwaite
##   approximations to degrees of freedom [lmerMod]
## Formula: wow ~ personalclass + Agreeableness + (1 | State)
##    Data: mrbclean
##
##      AIC      BIC   logLik deviance df.resid
##   1382.8   1406.4   -686.4   1372.8      828
##
## Scaled residuals:
##      Min       1Q   Median       3Q      Max
## -2.99947 -0.65909  0.06785  0.70380  2.92753
##
## Random effects:
##  Groups   Name        Variance              Std.Dev.
##  State    (Intercept) 0.0000000000000009557 0.00000003091
##  Residual             0.3042471793442670380 0.55158605797
## Number of obs: 833, groups:  State, 48
##
## Fixed effects:
##                 Estimate Std. Error        df t value  Pr(>|t|)
## (Intercept)    -0.46192    0.11456 833.00000  -4.032 0.0000604 ***
## personalclass  -0.24576    0.05846 833.00000  -4.204 0.0000291 ***
## Agreeableness   0.12961    0.03150 833.00000   4.115 0.0000426 ***
## ---
## Signif. codes:  0 '***' 0.001 '**' 0.01 '*' 0.05 '.' 0.1 ' ' 1
##
## Correlation of Fixed Effects:
##             (Intr) prsnlc
## personlclss -0.002
## Agreeablnss -0.986  0.010
```

control for individual differences in other-vs. self-focus: recognition on/focus of personal vs. other’s emotions effect of social class when controlling for focus on others’ emotions

```
summary(lmer(wow ~ personalclass+ EISelfEmotions+EIOthersEmotions
                    +(1|State), mrbclean, REML=FALSE))
```

```
## Linear mixed model fit by maximum likelihood t-tests use Satterthwaite
##   approximations to degrees of freedom [lmerMod]
## Formula: wow ~ personalclass + EISelfEmotions + EIOthersEmotions + (1 |
##     State)
##    Data: mrbclean
##
##      AIC      BIC   logLik deviance df.resid
##   1075.5   1102.3   -531.8   1063.5      631
##
## Scaled residuals:
##     Min      1Q  Median      3Q     Max
## -3.2513 -0.6522  0.1307  0.7146  3.1574
##
## Random effects:
##  Groups   Name        Variance Std.Dev.
##  State    (Intercept) 0.0000   0.0000
##  Residual             0.3109   0.5576
## Number of obs: 637, groups:  State, 48
##
## Fixed effects:
##                   Estimate Std. Error         df t value   Pr(>|t|)
## (Intercept)      -0.549196   0.120231 637.000000  -4.568 0.00000592 ***
## personalclass    -0.270731   0.067312 637.000000  -4.022 0.00006462 ***
## EISelfEmotions    0.003732   0.021162 637.000000   0.176       0.86
## EIOthersEmotions   0.100883   0.022930 637.000000   4.400 0.00001271 ***
## ---
## Signif. codes:  0 '***' 0.001 '**' 0.01 '*' 0.05 '.' 0.1 ' ' 1
##
## Correlation of Fixed Effects:
##             (Intr) prsnlc EISlfE
## personlclss  0.034
## EISelfEmtns -0.447 -0.040
## EIOthrsEmtn -0.556  0.020 -0.477
```

notably, there are no class differences in recognition of/focus on others' emotions

```
summary(lmer(EIOthersEmotions ~ personalclass+(1|State), mrbclean, REML=FALSE))
```

```
## Linear mixed model fit by maximum likelihood t-tests use Satterthwaite
##   approximations to degrees of freedom [lmerMod]
## Formula: EIOthersEmotions ~ personalclass + (1 | State)
##    Data: mrbclean
##
##      AIC      BIC   logLik deviance df.resid
##   1932.9   1950.7   -962.4   1924.9      633
##
## Scaled residuals:
##     Min      1Q  Median      3Q     Max
## -3.8508 -0.6524  0.0255  0.7071  1.6300
##
## Random effects:
##  Groups   Name        Variance Std.Dev.
##  State    (Intercept) 0.001157 0.03401
##  Residual             1.200725 1.09578
## Number of obs: 637, groups:  State, 48
##
## Fixed effects:
##                 Estimate Std. Error         df t value            Pr(>|t|)
## (Intercept)     5.222813   0.044126  13.600000 118.361 <0.0000000000000002
## personalclass  -0.002834   0.132315 577.400000  -0.021               0.983
##
## (Intercept)   ***
## personalclass
## ---
## Signif. codes:  0 '***' 0.001 '**' 0.01 '*' 0.05 '.' 0.1 ' ' 1
##
## Correlation of Fixed Effects:
##             (Intr)
## personlclss 0.090
```

WITHIN-SITUATIONAL STATUS EFFECTS

BASED ON SAMPLES REPORTING THEIR STATUS IN THE SITUATION

```
#convert status (higher score means higher status of another person)
mrbclean$sitstatus<-mrbclean$status*(-1)
mrbclean$sitstatusOR <- ordered(mrbclean$sitstatus,
                     levels = c(-5,-4,-3,-2,-1),
                     labels = c("Much Less", "Less", "Same/Similar","More","Much More"))
```

first, standardize wise reasoning scores on this sub=sample

```
mrbclean.sit<-subset(mrbclean,sitstatusOR!="NA")
mrbclean.sit$wow<-scale(mrbclean.sit$wow)
```

means and confidence interval of wise reasoning at each status level

```
apa.1way.table(sitstatusOR,wow,mrbclean.sit,show.conf.interval=T)
```

```
##
##
## Descriptive statistics for wow as a function of sitstatusOR.
##
##   sitstatusOR     M    LL    UL   SD
##     Much Less  0.11 -0.14  0.36 1.09
##          Less  0.10 -0.06  0.25 0.89
##  Same/Similar  0.02 -0.09  0.13 0.92
##          More  0.08 -0.08  0.24 0.99
##     Much More -0.43 -0.68 -0.18 1.21
##
## Note. M and SD represent mean and standard deviation, respectively.
## LL and UL indicate the lower and upper limits of the 95% confidence interval
## for the mean, respectively.
## The confidence interval is a plausible range of population means that could
## have caused a sample mean (Cumming, 2014).
```

```
status.Wclass.dv<-lm(wow ~ sitstatus, data=mrbclean.sit)
#greater status (vs. status of the other person in a situation) linked to less wise reasoning
summary(status.Wclass.dv)
```

```
##
## Call:
## lm(formula = wow ~ sitstatus, data = mrbclean.sit)
##
## Residuals:
##      Min       1Q   Median       3Q      Max
## -2.69384 -0.56689  0.05205  0.63026  2.65034
##
## Coefficients:
##             Estimate Std. Error t value Pr(>|t|)
## (Intercept) -0.29817    0.10200  -2.923  0.00357 **
## sitstatus   -0.10143    0.03236  -3.134  0.00179 **
## ---
## Signif. codes:  0 '***' 0.001 '**' 0.01 '*' 0.05 '.' 0.1 ' ' 1
##
## Residual standard error: 0.994 on 728 degrees of freedom
## Multiple R-squared:  0.01331,    Adjusted R-squared:  0.01196
## F-statistic: 9.822 on 1 and 728 DF,  p-value: 0.001794
```

```
apa.reg.table(status.Wclass.dv)
```

```
## Warning: package 'bindrcpp' was built under R version 3.3.3
```

```
##
##
## Regression results using wow as the criterion
##
##
##    Predictor       b       b_95%_CI  beta    beta_95%_CI sr2 sr2_95%_CI
##  (Intercept) -0.30** [-0.50, -0.10]
##    sitstatus -0.10** [-0.16, -0.04] -0.12 [-0.19, -0.04] .01 [.00, .03]
##
##
##
##       r             Fit
##
##  -.12**
##             R2 = .013**
##         95% CI[.00,.03]
##
##
## Note. * indicates p < .05; ** indicates p < .01.
## A significant b-weight indicates the beta-weight and semi-partial correlation are also signif
icant.
## b represents unstandardized regression weights; beta indicates the standardized regression we
ights;
## sr2 represents the semi-partial correlation squared; r represents the zero-order correlation.
## Square brackets are used to enclose the lower and upper limits of a confidence interval.
##
```

Situational status independently contributes above and beyond

state and individual difference effects of status

```
summary(lmer(wow ~ sitstatus+personalclass+(1|State), mrbclean.sit, REML=FALSE))
```

```
## Linear mixed model fit by maximum likelihood t-tests use Satterthwaite
##   approximations to degrees of freedom [lmerMod]
## Formula: wow ~ sitstatus + personalclass + (1 | State)
##    Data: mrbclean.sit
##
##      AIC      BIC   logLik deviance df.resid
##   2061.0   2083.9  -1025.5   2051.0      725
##
## Scaled residuals:
##     Min      1Q  Median      3Q     Max
## -2.6157 -0.5959  0.0607  0.6527  2.6524
##
## Random effects:
##  Groups   Name        Variance Std.Dev.
##  State    (Intercept) 0.003007 0.05483
##  Residual             0.969181 0.98447
## Number of obs: 730, groups:  State, 47
##
## Fixed effects:
##                Estimate Std. Error        df t value Pr(>|t|)
## (Intercept)    -0.31502    0.10191 417.00000  -3.091 0.002127 **
## sitstatus      -0.10917    0.03216 729.60000  -3.394 0.000726 ***
## personalclass  -0.47973    0.15181 558.30000  -3.160 0.001663 **
## ---
## Signif. codes:  0 '***' 0.001 '**' 0.01 '*' 0.05 '.' 0.1 ' ' 1
##
## Correlation of Fixed Effects:
##             (Intr) ststts
## sitstatus   0.927
## personlclss 0.034  0.063
```

independent effects of situational and individual social status, no interaction.

```
summary(lmer(wow ~ scale(sitstatus,scale=F)*scale(personalclass,scale=F)+
               (1|State), mrbclean.sit, REML=FALSE))
```

```
## Linear mixed model fit by maximum likelihood t-tests use Satterthwaite
##   approximations to degrees of freedom [lmerMod]
## Formula:
## wow ~ scale(sitstatus, scale = F) * scale(personalclass, scale = F) +
##     (1 | State)
##    Data: mrbclean.sit
##
##      AIC      BIC   logLik deviance df.resid
##   2062.5   2090.1  -1025.3   2050.5      724
##
## Scaled residuals:
##      Min       1Q   Median       3Q      Max
## -2.60416 -0.59751  0.05577  0.64310  2.66011
##
## Random effects:
##  Groups   Name        Variance Std.Dev.
##  State    (Intercept) 0.002978 0.05457
##  Residual             0.968638 0.98419
## Number of obs: 730, groups:  State, 47
##
## Fixed effects:
##                                                                 Estimate
## (Intercept)                                                   0.00001126
## scale(sitstatus, scale = F)                                  -0.10982490
## scale(personalclass, scale = F)                              -0.47900076
## scale(sitstatus, scale = F):scale(personalclass, scale = F)   0.08504498
##                                                               Std. Error
## (Intercept)                                                   0.03814722
## scale(sitstatus, scale = F)                                   0.03217074
## scale(personalclass, scale = F)                               0.15176298
## scale(sitstatus, scale = F):scale(personalclass, scale = F)   0.13006774
##                                                                       df
## (Intercept)                                                  22.90000000
## scale(sitstatus, scale = F)                                 729.50000000
## scale(personalclass, scale = F)                             558.10000000
## scale(sitstatus, scale = F):scale(personalclass, scale = F) 729.50000000
##                                                             t value
## (Intercept)                                                   0.000
## scale(sitstatus, scale = F)                                  -3.414
## scale(personalclass, scale = F)                              -3.156
## scale(sitstatus, scale = F):scale(personalclass, scale = F)   0.654
##                                                             Pr(>|t|)
## (Intercept)                                                 0.999767
## scale(sitstatus, scale = F)                                 0.000676 ***
## scale(personalclass, scale = F)                             0.001684 **
## scale(sitstatus, scale = F):scale(personalclass, scale = F) 0.513414
## ---
## Signif. codes:  0 '***' 0.001 '**' 0.01 '*' 0.05 '.' 0.1 ' ' 1
##
## Correlation of Fixed Effects:
##             (Intr) scl(s,s=F) scl(p,s=F)
## scl(st,s=F)  0.000
```

```
## scl(pr,s=F) -0.002  0.063
## s(,s=F):s=F  0.062 -0.032      0.007
```

TEST MEDIATION THROUGH CLOSENESS

(lower status associated with greater closeness to the others involved in the incident) (higher status associated with less closeness to others involved in the incident)

```
status.Wclass.meddv<-lm(wow ~ sitstatus +closebefor, data=mrbclean.sit)
```

effect of lower status and greater closeness.

```
summary(status.Wclass.meddv)
```

```
##
## Call:
## lm(formula = wow ~ sitstatus + closebefor, data = mrbclean.sit)
##
## Residuals:
##      Min       1Q   Median       3Q      Max
## -2.64557 -0.58993 -0.00291  0.61525  2.78294
##
## Coefficients:
##             Estimate Std. Error t value    Pr(>|t|)
## (Intercept) -0.64540    0.12139  -5.317 0.000000141 ***
## sitstatus   -0.08771    0.03194  -2.746     0.00618 **
## closebefor   0.22835    0.04496   5.079 0.000000483 ***
## ---
## Signif. codes:  0 '***' 0.001 '**' 0.01 '*' 0.05 '.' 0.1 ' ' 1
##
## Residual standard error: 0.9775 on 727 degrees of freedom
## Multiple R-squared:  0.04712,    Adjusted R-squared:  0.0445
## F-statistic: 17.98 on 2 and 727 DF,  p-value: 0.00000002399
```

```
status.Wclass.dv<-lm(wow ~ sitstatus , data=mrbclean.sit)
status.Wclass.med<-lm(closebefor ~ sitstatus, data=mrbclean.sit)
```

Effect of status on closeness

```
summary(status.Wclass.med)
```

```
##
## Call:
## lm(formula = closebefor ~ sitstatus, data = mrbclean.sit)
##
## Residuals:
##     Min      1Q  Median      3Q     Max
## -0.8210 -0.7009 -0.5807  0.3592  2.4193
##
## Coefficients:
##             Estimate Std. Error t value            Pr(>|t|)
## (Intercept)  1.52063    0.08269   18.39 <0.0000000000000002 ***
## sitstatus   -0.06008    0.02624   -2.29              0.0223 *
## ---
## Signif. codes:  0 '***' 0.001 '**' 0.01 '*' 0.05 '.' 0.1 ' ' 1
##
## Residual standard error: 0.8058 on 728 degrees of freedom
## Multiple R-squared:  0.007153,   Adjusted R-squared:  0.005789
## F-statistic: 5.245 on 1 and 728 DF,  p-value: 0.0223
```

```
result.mediation<-mediate(status.Wclass.med,status.Wclass.meddv, treat = "sitstatus",
mediator = "closebefor", sims = 3000)
```

sig mediation of status on wise reasoning via closeness - note, it is partial -

there is a direct effect of status as well.

```
summary.mediate(result.mediation)
```

```
##
## Causal Mediation Analysis
##
## Quasi-Bayesian Confidence Intervals
##
##                 Estimate 95% CI Lower 95% CI Upper p-value
## ACME            -0.01376     -0.02843     -0.00187    0.02
## ADE             -0.08783     -0.15017     -0.02306    0.01
## Total Effect    -0.10159     -0.16513     -0.03615    0.00
## Prop. Mediated   0.12972      0.01916      0.40170    0.02
##
## Sample Size Used: 730
##
##
## Simulations: 3000
```

```
plot.mediate(result.mediation)
```

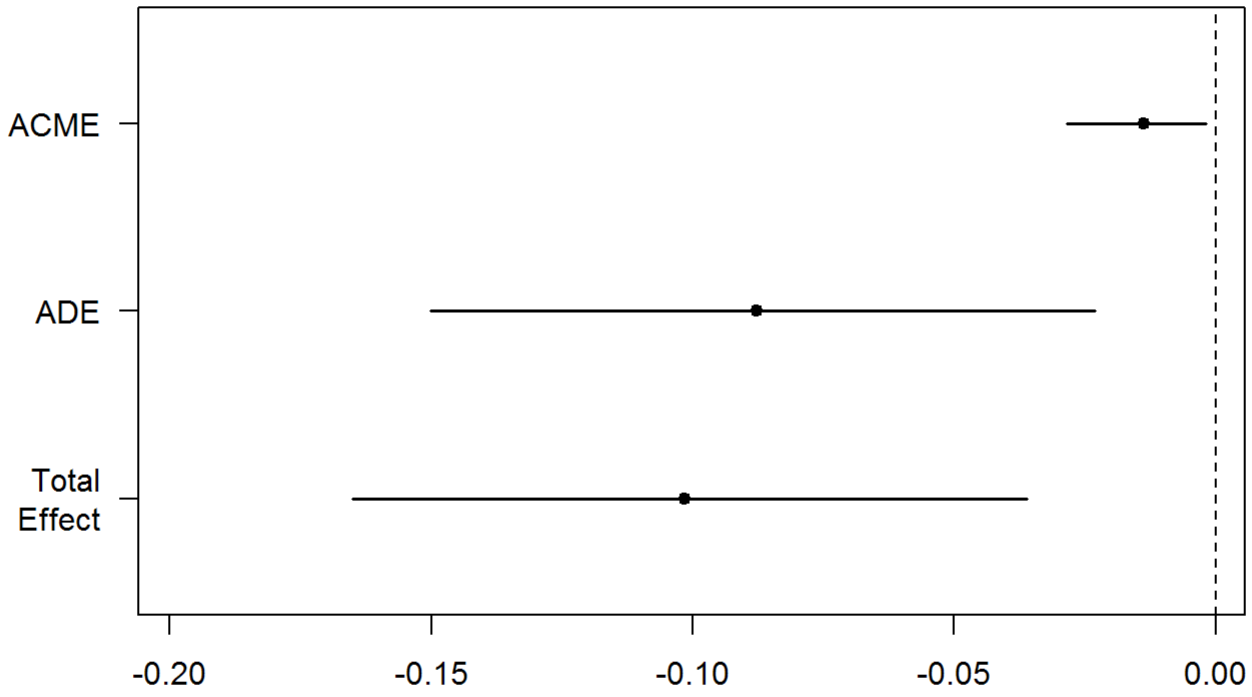

sub-dimension analyses

HUMILITY

```
status.Wclass.meddv1<-lm(humility ~ sitstatus +closebefor, data=mrbclean.sit)
```

effect of status and closeness on humility.

```
summary(status.Wclass.meddv1)
```

```
##
## Call:
## lm(formula = humility ~ sitstatus + closebefor, data = mrbclean.sit)
##
## Residuals:
##      Min       1Q   Median       3Q      Max
## -1.96575 -0.50874  0.01655  0.50168  2.05254
##
## Coefficients:
##              Estimate Std. Error t value     Pr(>|t|)
## (Intercept) -0.55622    0.09983  -5.571 0.0000000356 ***
## sitstatus   -0.06997    0.02627  -2.664       0.0079 **
## closebefor   0.17134    0.03698   4.634 0.0000042507 ***
## ---
## Signif. codes:  0 '***' 0.001 '**' 0.01 '*' 0.05 '.' 0.1 ' ' 1
##
## Residual standard error: 0.8039 on 727 degrees of freedom
## Multiple R-squared:  0.04074,    Adjusted R-squared:  0.03811
## F-statistic: 15.44 on 2 and 727 DF,  p-value: 0.0000002711
```

```
status.Wclass.dv1<-lm(humility ~ sitstatus, data=mrbclean.sit)
result.mediation1<-mediate(status.Wclass.med,status.Wclass.meddv1, treat = "sitstatus",
                           mediator = "closebefor", sims = 2000)
```

sig mediation of status on humility via closeness

note, it is partial - there is a direct effect of status as well.

```
summary.mediate(result.mediation1)
```

```
##
## Causal Mediation Analysis
##
## Quasi-Bayesian Confidence Intervals
##
##                 Estimate 95% CI Lower 95% CI Upper p-value
## ACME            -0.01031     -0.02146     -0.00103    0.02
## ADE             -0.07056     -0.12412     -0.02019    0.01
## Total Effect    -0.08087     -0.13466     -0.03041    0.00
## Prop. Mediated   0.12439      0.01376      0.38915    0.03
##
## Sample Size Used: 730
##
##
## Simulations: 2000
```

```
plot.mediate(result.mediation1)
```

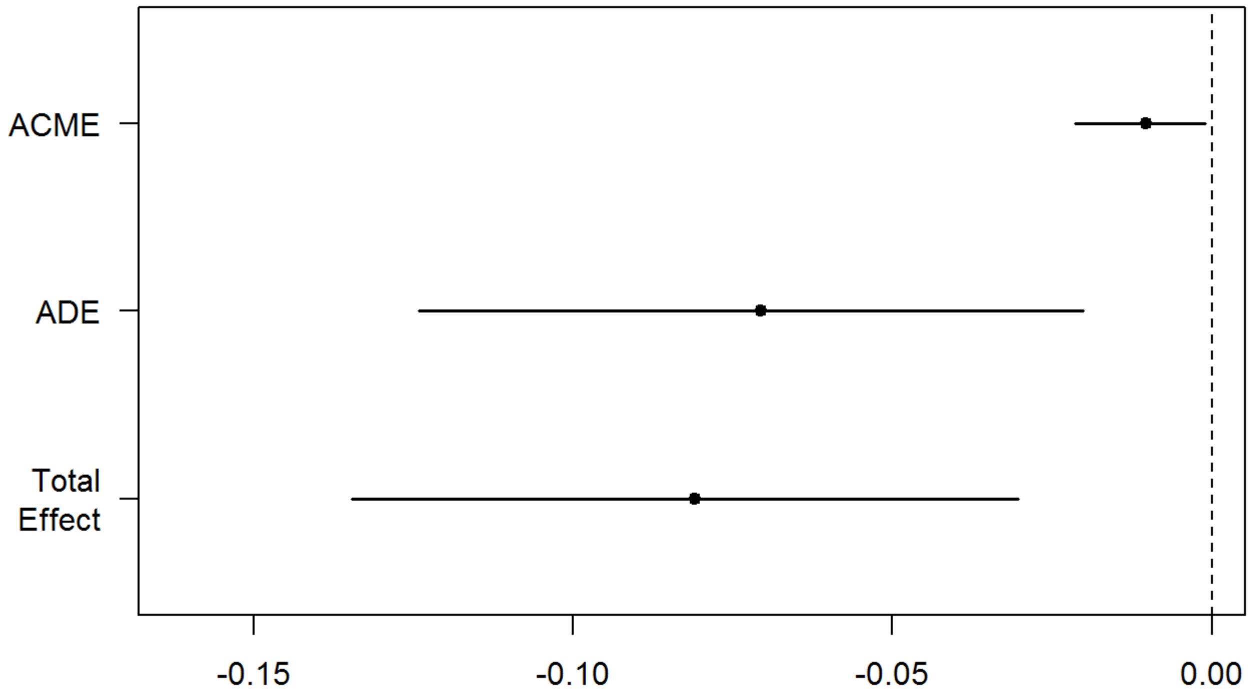

CHANGE

```
status.Wclass.meddv2<-lm(change ~ sitstatus +closebefor, data=mrbclean.sit)
```

effect of status and closeness on recognition of change.

```
summary(status.Wclass.meddv2)
```

```
##
## Call:
## lm(formula = change ~ sitstatus + closebefor, data = mrbclean.sit)
##
## Residuals:
##      Min       1Q   Median       3Q      Max
## -2.16760 -0.51569  0.03445  0.55636  2.10098
##
## Coefficients:
##             Estimate Std. Error t value      Pr(>|t|)
## (Intercept) -0.59456    0.10229  -5.813 0.00000000921 ***
## sitstatus   -0.06866    0.02691  -2.551      0.010937 *
## closebefor   0.14387    0.03788   3.798      0.000158 ***
## ---
## Signif. codes:  0 '***' 0.001 '**' 0.01 '*' 0.05 '.' 0.1 ' ' 1
##
## Residual standard error: 0.8237 on 727 degrees of freedom
## Multiple R-squared:  0.03032,    Adjusted R-squared:  0.02765
## F-statistic: 11.37 on 2 and 727 DF,  p-value: 0.00001378
```

```
status.Wclass.dv2<-lm(change ~ sitstatus , data=mrbclean.sit)
result.mediation2<-mediate(status.Wclass.med,status.Wclass.meddv2, treat = "sitstatus",
                           mediator = "closebefor", sims = 2000)
```

sig mediation of status on change via closeness

note, it is partial - there is a direct effect of status as well.

```
summary.mediate(result.mediation2)
```

```
##
## Causal Mediation Analysis
##
## Quasi-Bayesian Confidence Intervals
##
##                Estimate 95% CI Lower 95% CI Upper p-value
## ACME           -0.00871     -0.01892     -0.00129    0.02
## ADE            -0.06821     -0.12153     -0.01729    0.01
## Total Effect   -0.07692     -0.13094     -0.02642    0.00
## Prop. Mediated  0.10819      0.01541      0.38113    0.02
##
## Sample Size Used: 730
##
##
## Simulations: 2000
```

```
plot.mediate(result.mediation2)
```

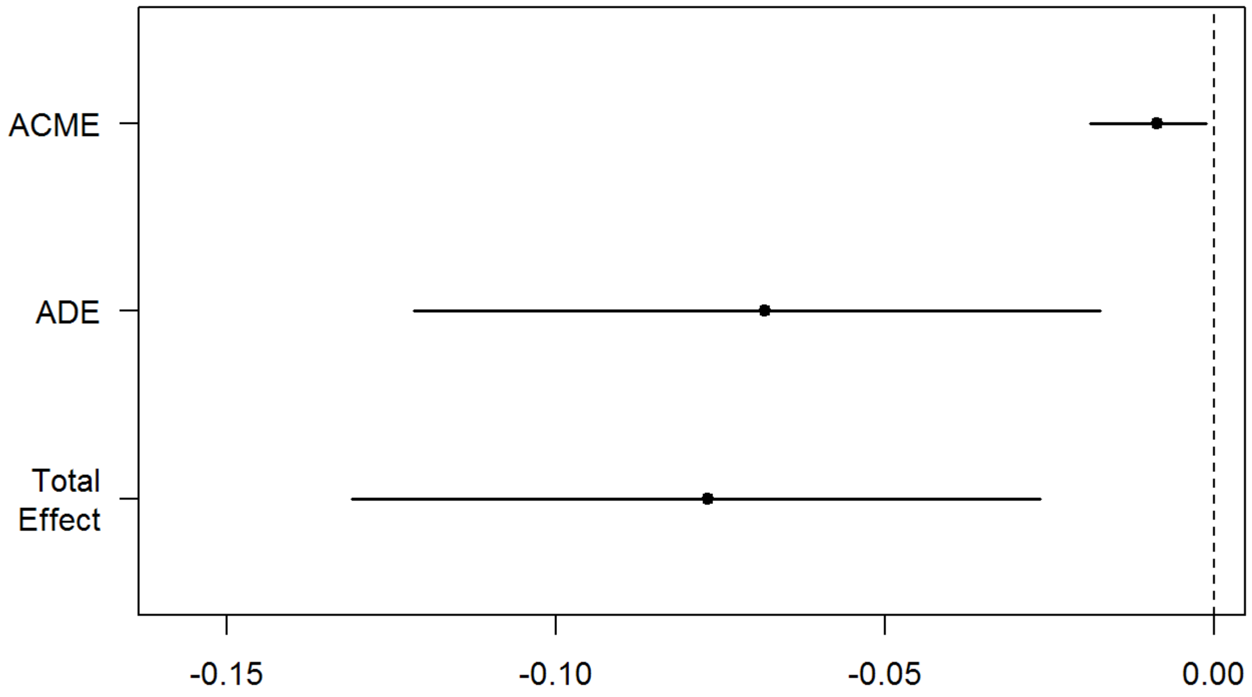

PERSP

```
status.Wclass.meddv3<-lm(persp ~ sitstatus +closebefor, data=mrbclean.sit)
```

effect of status and closeness on consideration of others' perspectives

```
summary(status.Wclass.meddv3)
```

```
##
## Call:
## lm(formula = persp ~ sitstatus + closebefor, data = mrbclean.sit)
##
## Residuals:
##      Min       1Q   Median       3Q      Max
## -1.72770 -0.43766  0.01806  0.49199  1.92984
##
## Coefficients:
##             Estimate Std. Error t value        Pr(>|t|)
## (Intercept) -0.59647    0.08653  -6.893 0.0000000000119 ***
## sitstatus   -0.05891    0.02277  -2.588         0.00986 **
## closebefor   0.17131    0.03205   5.345 0.0000001210497 ***
## ---
## Signif. codes:  0 '***' 0.001 '**' 0.01 '*' 0.05 '.' 0.1 ' ' 1
##
## Residual standard error: 0.6968 on 727 degrees of freedom
## Multiple R-squared:  0.04952,    Adjusted R-squared:  0.04691
## F-statistic: 18.94 on 2 and 727 DF,  p-value: 0.0000000096
```

```
status.Wclass.dv3<-lm(persp ~ sitstatus , data=mrbclean.sit)
result.mediation3<-mediate(status.Wclass.med,status.Wclass.meddv3, treat = "sitstatus",
                           mediator = "closebefor", sims = 2000)
```

sig mediation of status on perspective-taking via closeness

note, it is partial - there is a direct effect of status as well

```
summary.mediate(result.mediation3)
```

```
##
## Causal Mediation Analysis
##
## Quasi-Bayesian Confidence Intervals
##
##                Estimate 95% CI Lower 95% CI Upper p-value
## ACME           -0.01039     -0.02128     -0.00165    0.02
## ADE            -0.05868     -0.10325     -0.01106    0.01
## Total Effect   -0.06907     -0.11364     -0.02103    0.00
## Prop. Mediated  0.14784      0.02160      0.47946    0.02
##
## Sample Size Used: 730
##
##
## Simulations: 2000
```

```
plot.mediate(result.mediation3)
```

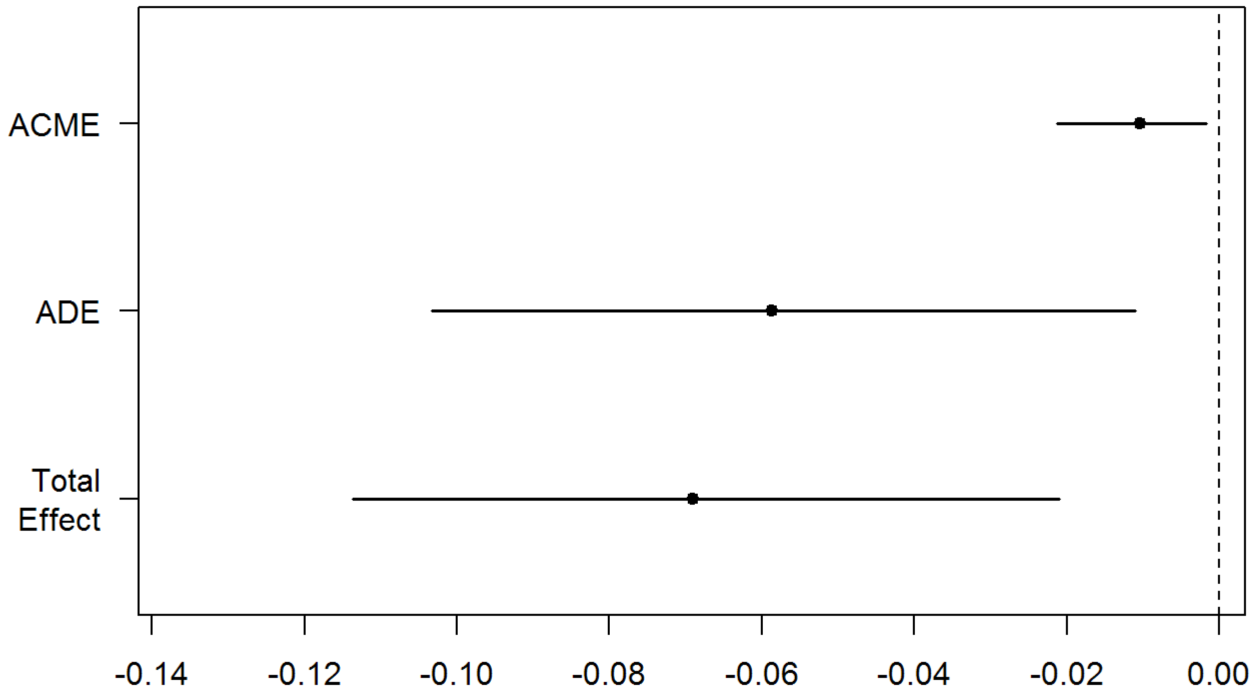

COMPR/RES

```
status.Wclass.meddv4<-lm(comprres ~ sitstatus +closebefor, data=mrbclean.sit)
```

effect of status and closeness on compromise/search for resolution

```
summary(status.Wclass.meddv4)
```

```
##
## Call:
## lm(formula = comprres ~ sitstatus + closebefor, data = mrbclean.sit)
##
## Residuals:
##      Min       1Q   Median       3Q      Max
## -2.14104 -0.48388  0.03849  0.52401  2.15720
##
## Coefficients:
##             Estimate Std. Error t value        Pr(>|t|)
## (Intercept) -0.66414    0.09970  -6.661 0.0000000000536 ***
## sitstatus   -0.06585    0.02623  -2.510          0.0123 *
## closebefor   0.18725    0.03693   5.071 0.0000005025867 ***
## ---
## Signif. codes:  0 '***' 0.001 '**' 0.01 '*' 0.05 '.' 0.1 ' ' 1
##
## Residual standard error: 0.8028 on 727 degrees of freedom
## Multiple R-squared:  0.0452, Adjusted R-squared:  0.04257
## F-statistic: 17.21 on 2 and 727 DF,  p-value: 0.00000004991
```

```
status.Wclass.dv4<-lm(comprres ~ sitstatus , data=mrbclean.sit)
result.mediation4<-mediate(status.Wclass.med,status.Wclass.meddv4, treat = "sitstatus",
                           mediator = "closebefor", sims = 2000)
```

sig mediation of status on compromise via closeness

note, it is partial - there is a direct effect of status as well.

```
summary.mediate(result.mediation4)
```

```
##
## Causal Mediation Analysis
##
## Quasi-Bayesian Confidence Intervals
##
##                Estimate 95% CI Lower 95% CI Upper p-value
## ACME           -0.01141     -0.02262     -0.00186    0.01
## ADE            -0.06548     -0.11606     -0.01680    0.01
## Total Effect   -0.07689     -0.13032     -0.02685    0.01
## Prop. Mediated  0.14613      0.02520      0.42753    0.02
##
## Sample Size Used: 730
##
##
## Simulations: 2000
```

```
plot.mediate(result.mediation4)
```

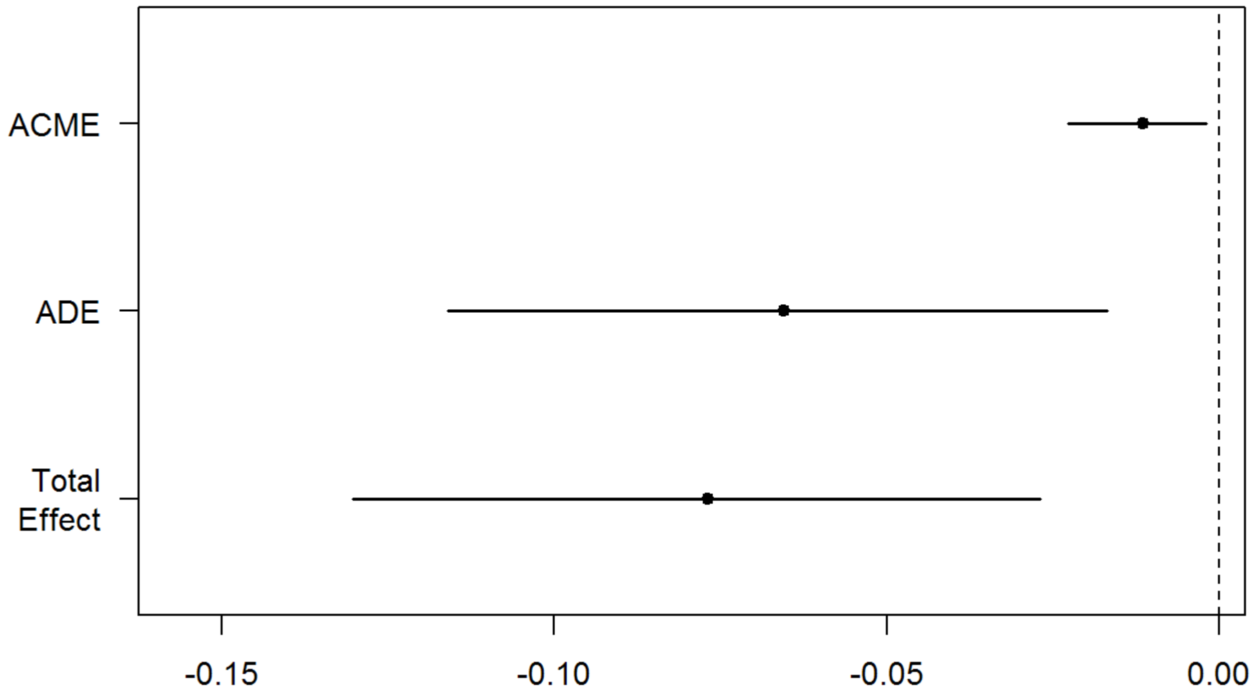

OUTSIDER

```
status.Wclass.meddv5<-lm(outsider ~ sitstatus +closebefor, data=mrbclean.sit)
```

effect of status and marg effect of closeness on outsider viewpoint

```
summary(status.Wclass.meddv5)
```

```
##
## Call:
## lm(formula = outsider ~ sitstatus + closebefor, data = mrbclean.sit)
##
## Residuals:
##      Min       1Q   Median       3Q      Max
## -1.94630 -0.62900  0.03881  0.69369  2.08937
##
## Coefficients:
##             Estimate Std. Error t value Pr(>|t|)
## (Intercept) -0.31144    0.11627  -2.679  0.00756 **
## sitstatus   -0.07319    0.03059  -2.393  0.01698 *
## closebefor   0.08026    0.04306   1.864  0.06276 .
## ---
## Signif. codes:  0 '***' 0.001 '**' 0.01 '*' 0.05 '.' 0.1 ' ' 1
##
## Residual standard error: 0.9362 on 727 degrees of freedom
## Multiple R-squared:  0.0136, Adjusted R-squared:  0.01089
## F-statistic: 5.012 on 2 and 727 DF,  p-value: 0.00689
```

```
status.Wclass.dv5<-lm(outsider ~ sitstatus , data=mrbclean.sit)
result.mediation5<-mediate(status.Wclass.med,status.Wclass.meddv5, treat = "sitstatus",
                           mediator = "closebefor", sims = 2000)
```

marginal mediation of status on outsider perspective via closeness

note, the proportion mediated is above 5%

```
summary.mediate(result.mediation5)
```

```
##
## Causal Mediation Analysis
##
## Quasi-Bayesian Confidence Intervals
##
##                 Estimate 95% CI Lower 95% CI Upper p-value
## ACME           -0.004936    -0.013193     0.000337    0.08
## ADE            -0.072415    -0.130046    -0.012994    0.02
## Total Effect   -0.077352    -0.137015    -0.018102    0.01
## Prop. Mediated  0.057283    -0.007091     0.284872    0.09
##
## Sample Size Used: 730
##
##
## Simulations: 2000
```

```
plot.mediate(result.mediation5)
```

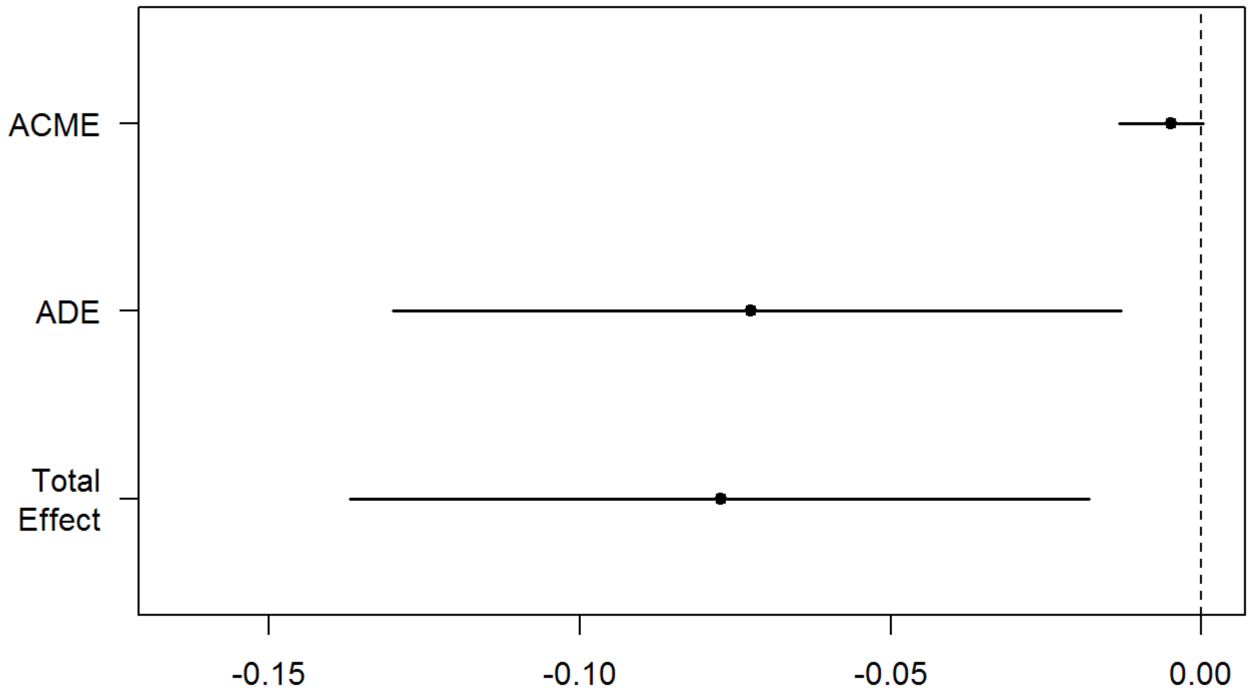

FIGURES FOR THE PAPER

State-level Status and Wise Reasoning across U.S. Regions

```
A1<-ggplot(estimatesStateclean, aes(x=stateclass, y=wow))
A2<-A1+geom_point(aes(size = n, colour=Region)) +
  geom_smooth(method=lm)+  # Add linear regression line
  scale_color_brewer(palette="Dark2")+
  theme_classic() +labs(x = "Status of the State (z-score)",
                        y = "Wise Reasoning Index (z-score)")
A2+theme(legend.position="right")+guides(colour=guide_legend(override.aes=list(size=4)))
```

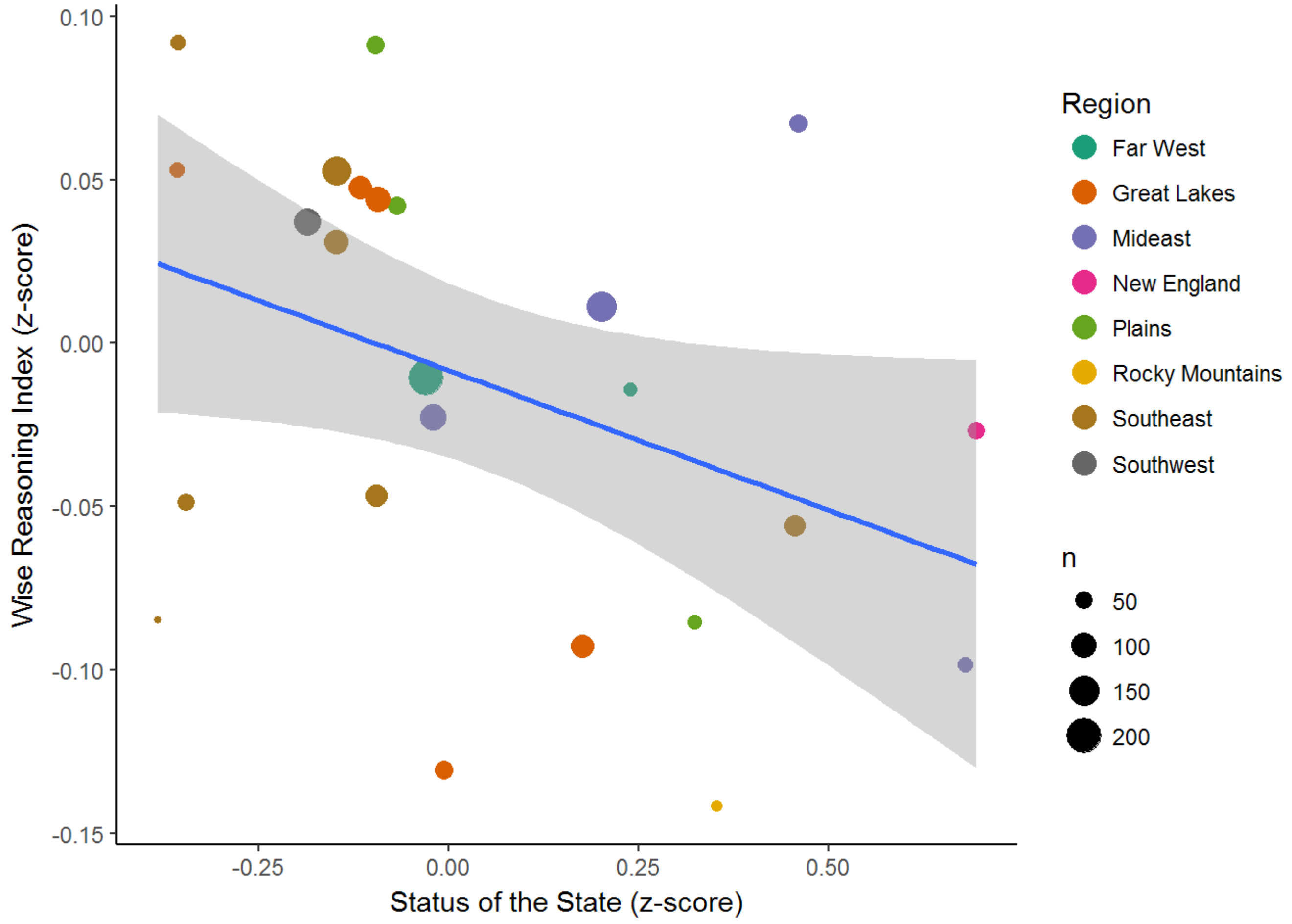

Individual-level Status and Wise Reasoning

```
ggplot(mrbclean, aes(x=personalclass, y=wow)) +
  geom_point(alpha = 1/6, size = 4) +
  geom_smooth(method=loess)+  # Add linear regression line
  theme_classic() +labs(x = "Individual Differences in Social Class (z-score)",
                        y = "Wise Reasoning Index (z-score)")
```

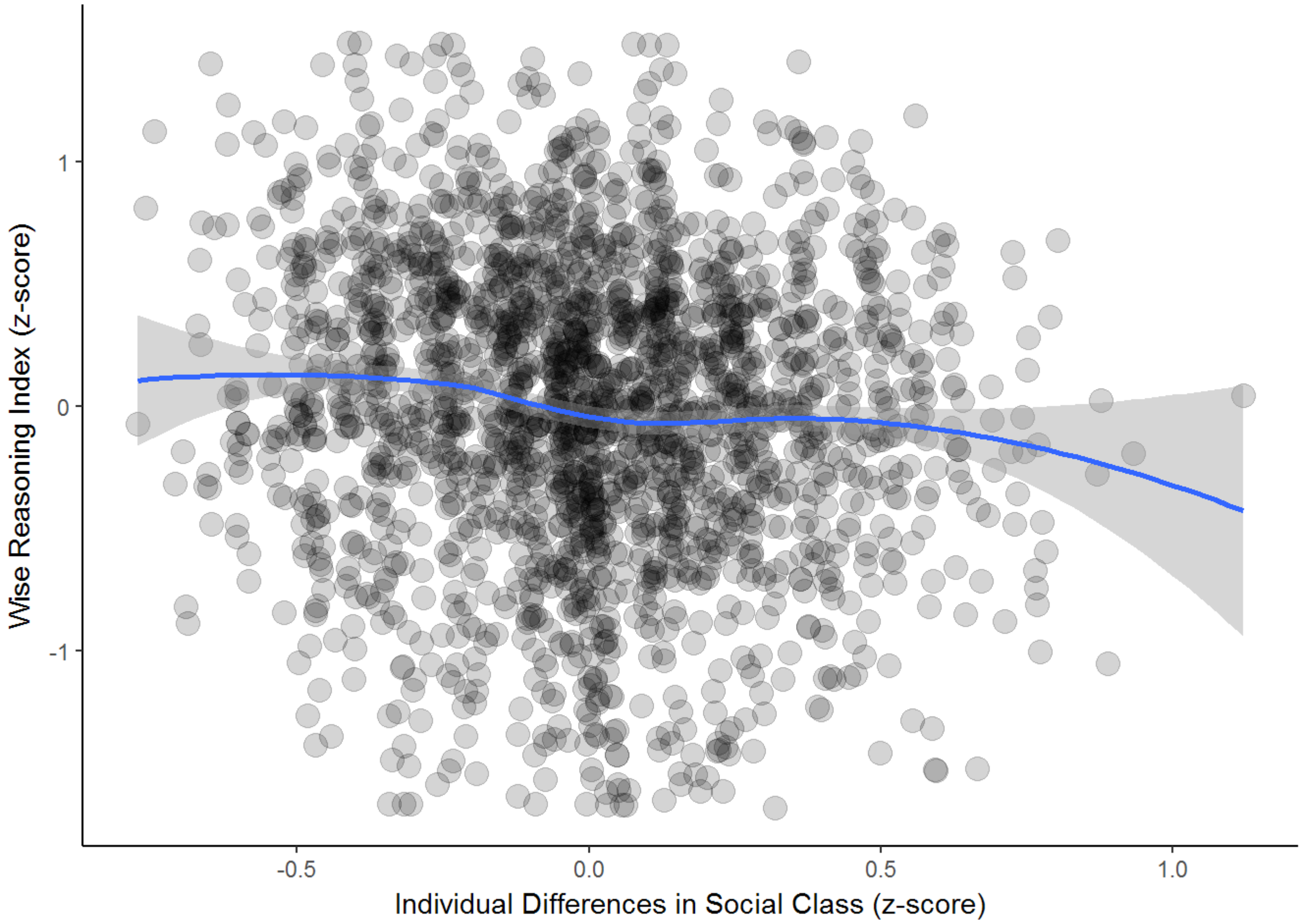

Situation-specific Status and Wise Reasoning

```
mrbcleanplot <- subset(mrbclean.sit, sitstatusOR  =="Much Less"|sitstatusOR=="Less"|sitstatusOR=
="Same/Similar"|
sitstatusOR=="More"|sitstatusOR=="Much More",
select=c(wow,sitstatusOR))
C1<-ggplot(mrbcleanplot, aes(x=sitstatusOR, y=wow, color=sitstatusOR)) +
             geom_violin(trim=FALSE,draw_quantiles = c(0.25, 0.5, 0.75),fill='gray')
C1+scale_colour_brewer(palette = "Dark2")+theme_classic()+ theme(legend.position="none")+
  scale_fill_distiller(guide = "colourbar")+geom_boxplot(width=0.1,color="black")+
  labs(x = "Personal vs. Others' Status in a Situation",
       y = "Wise Reasoning Index (z-score)")
```

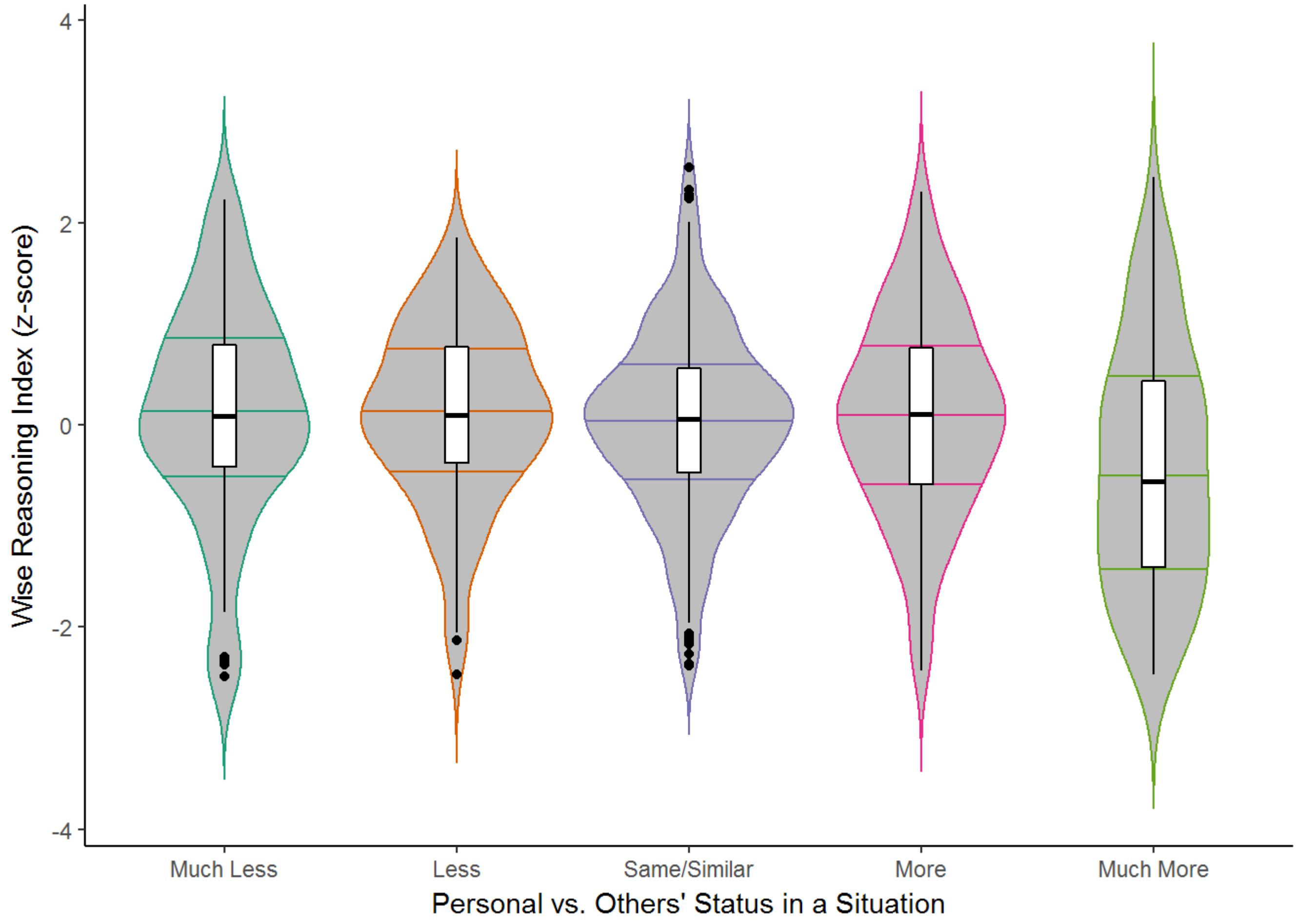

Situation-specific Status and Wise Reasoning - Means and St. Errors - ALTERNATIVE FOR SUPPLEMENT

```
mrbclean.sum <- lm(scale(Normalize(wow))~sitstatusOR,mrbclean.sit)

mrbcleanplot1<-summary(lsmeans(mrbclean.sum, ~sitstatusOR))
mrbcleanplot1<-as.data.frame(mrbcleanplot1)
ggplot(mrbcleanplot1, aes(x=sitstatusOR, y=lsmean, fill=sitstatusOR)) +
  geom_bar(stat="identity", color="black", position=position_dodge()) +
  scale_fill_brewer(palette = "Dark2")+
  geom_errorbar(aes(ymin=lsmean-SE, ymax=lsmean+SE), width=.2,
                position=position_dodge(.9))+scale_colour_brewer(palette = "Dark2")+
  theme_classic()+ theme(legend.position="none")+
  labs(x = "Personal vs. Others' Status in a Situation",
       y = "M +/- S.E. (Wise Reasoning Index: z-score)")
```

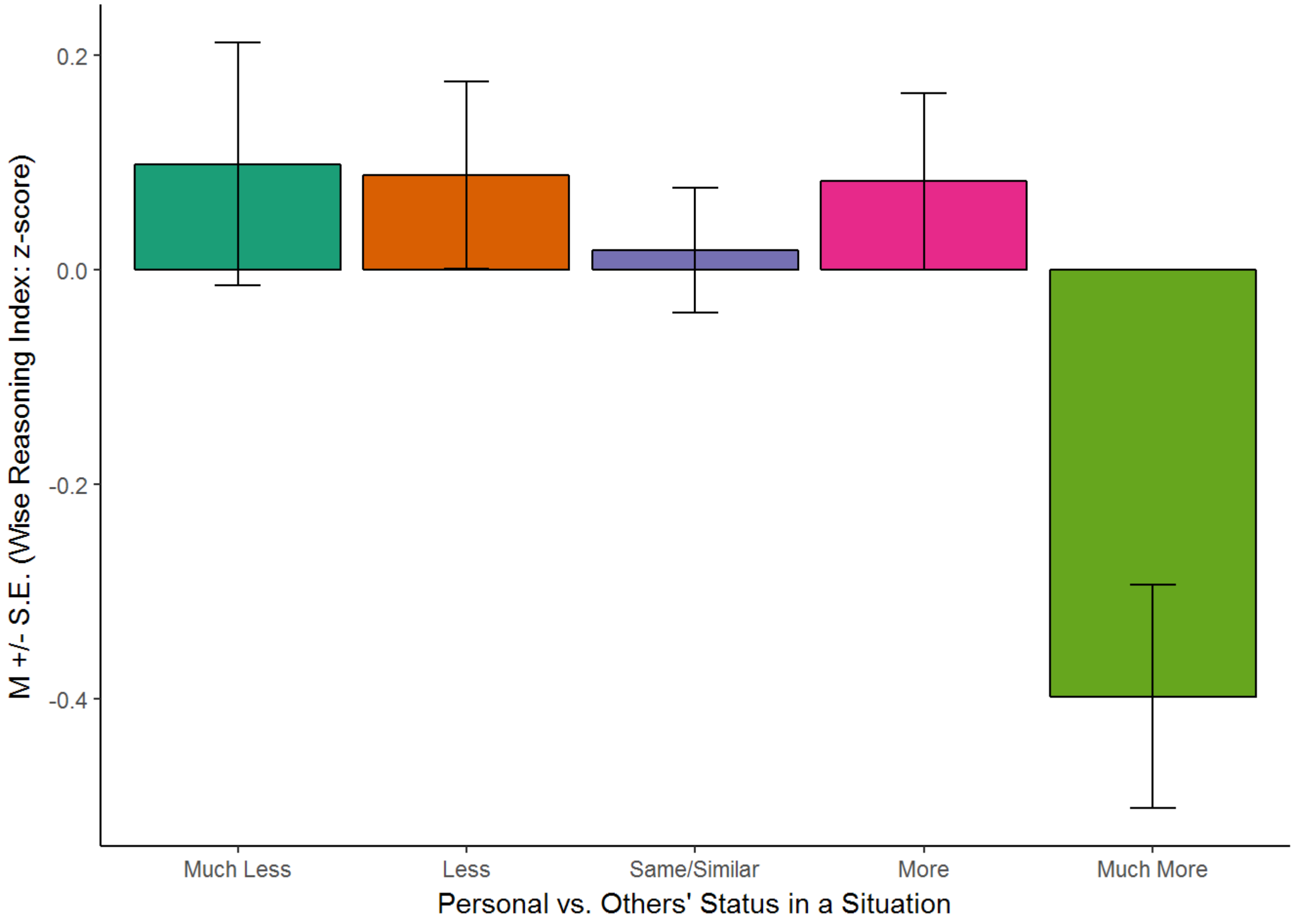

Study 2: PNAS by Grossmann et al., 2010

```
pnas<-read.spss(choose.files(), to.data.frame=TRUE, use.value.labels=T)
```

```
## Warning in read.spss(choose.files(), to.data.frame = TRUE, use.value.labels
## = T): C:\Users\igrossma\Google Drive\Drive\scales\social class\OLD wis
## PNAS data 19 Aug 2017.short.sav: Unrecognized record type 7, subtype 17
## encountered in system file
```

```
## Warning in read.spss(choose.files(), to.data.frame = TRUE, use.value.labels
## = T): C:\Users\igrossma\Google Drive\Drive\scales\social class\OLD wis
## PNAS data 19 Aug 2017.short.sav: Unrecognized record type 7, subtype 18
## encountered in system file
```

```
pnas.clean<-subset(pnas,edu_4!="NA"&age<40)

pnas.sum <- lm(scale((wisdom.interpersonal))~edu_4+WC.interpersonal.mean+ IQ.c+IQ.f+gender,pnas.
clean)

pnasplot1<-summary(lsmeans(pnas.sum, ~edu_4))
pnasplot1<-as.data.frame(pnasplot1)
ggplot(pnasplot1, aes(x=edu_4, y=lsmean, fill=edu_4)) +
  geom_bar(stat="identity", color="black", position=position_dodge()) +
  scale_fill_brewer(palette = "Dark2")+
  geom_errorbar(aes(ymin=lsmean-SE, ymax=lsmean+SE), width=.2,
                position=position_dodge(.9))+scale_colour_brewer(palette = "Dark2")+
  theme_classic()+ theme(legend.position="none")+
  labs(x = "Level of Education",
       y = "M +/- S.E. (Wise Reasoning Index: z-score)")
```

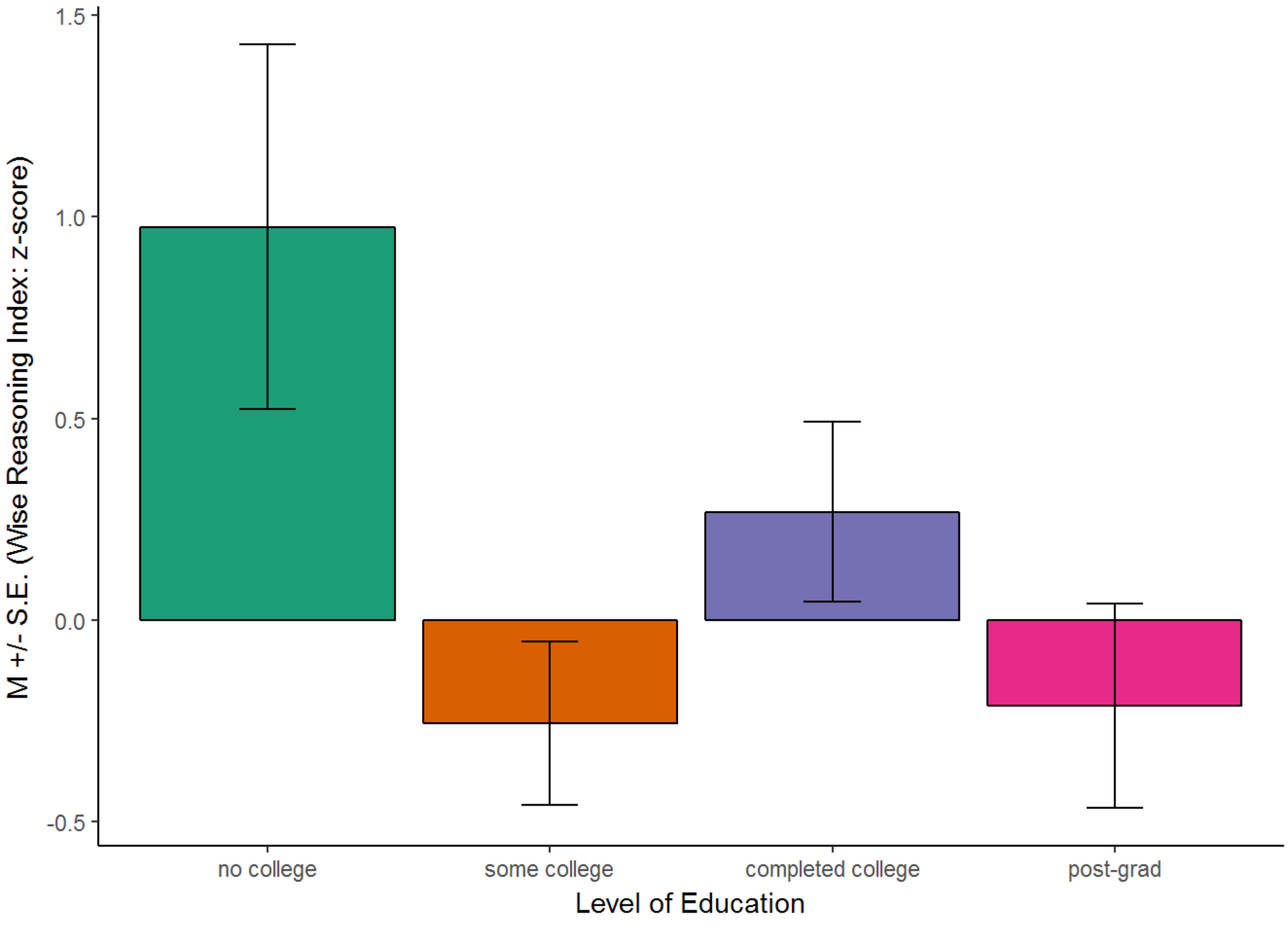

```
pnascleanplot <- subset(pnas.clean, edu_4  =="no college"|edu_4=="some college"|edu_4=="complete
d college"|
                         edu_4=="post-grad",select=c(wisdom.interpersonal,edu_4))


D1<-ggplot(pnascleanplot, aes(x=edu_4, y=scale(wisdom.interpersonal), color=edu_4)) +
  geom_violin(trim=FALSE,draw_quantiles = c(0.25, 0.5, 0.75),fill='gray')
D1+scale_colour_brewer(palette = "Dark2")+theme_classic()+ theme(legend.position="none")+
  scale_fill_distiller(guide = "colourbar")+geom_boxplot(width=0.1,color="black")+
  labs(x = "Level of Education",
       y = "Wise Reasoning Index (z-score)")
```

```
## Warning: Removed 20 rows containing non-finite values (stat_ydensity).
```

```
## Warning: Removed 20 rows containing non-finite values (stat_boxplot).
```

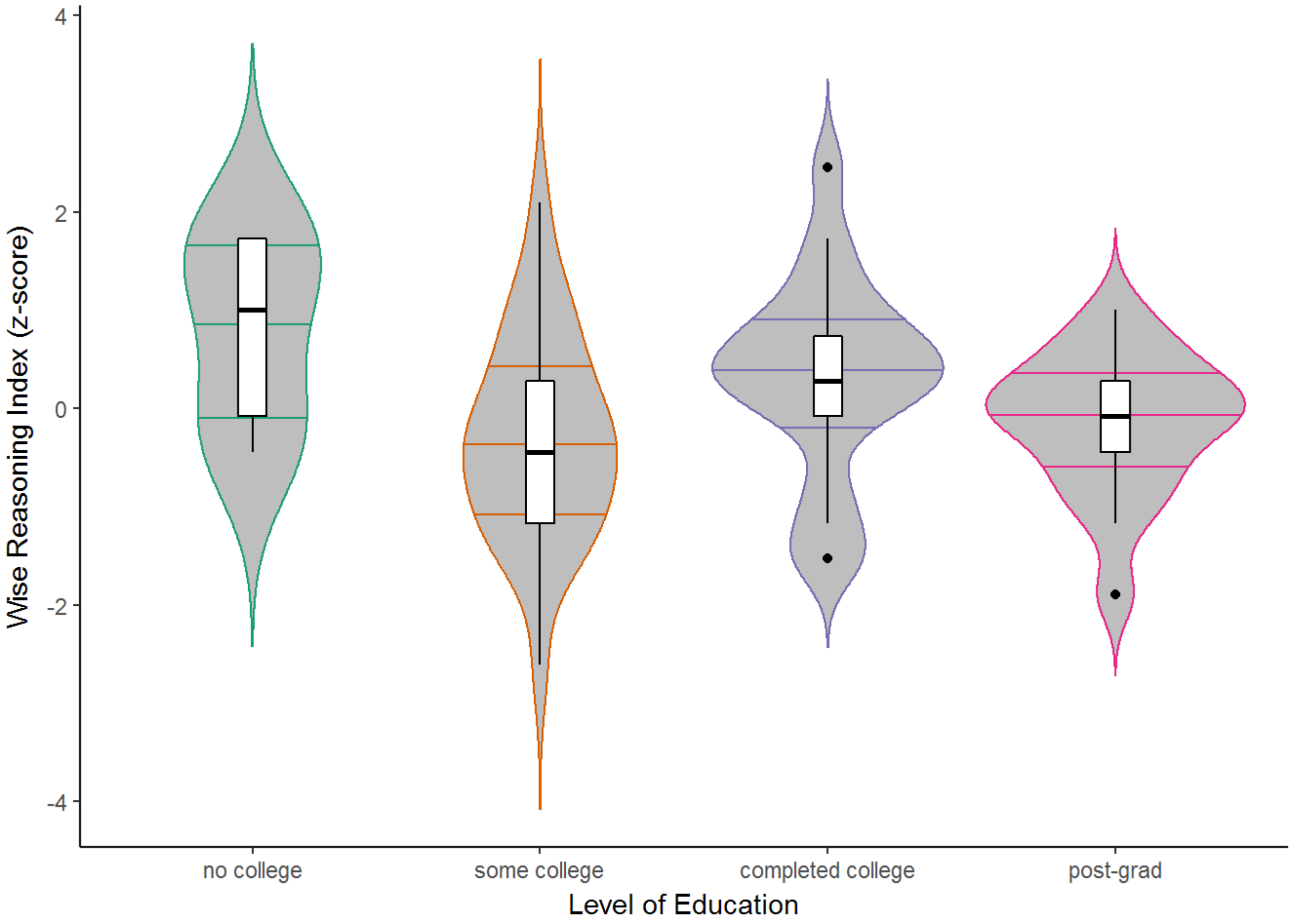

Supplement: Analysis code [file rspb20171870supp2.pdf]
